# Heterosis for Interactions between Insect Herbivores and 3-Line Hybrid Rice under Low and High Soil Nitrogen Conditions

**DOI:** 10.3390/insects15060416

**Published:** 2024-06-04

**Authors:** Finbarr G. Horgan, Carmencita C. Bernal, Angelee Fame Ramal, Maria Liberty P. Almazan, Enrique A. Mundaca, Eduardo Crisol-Martínez

**Affiliations:** 1 EcoLaVerna Integral Restoration Ecology, Bridestown, Kildinan, Co., T56 P499 Cork, Ireland; 2 School of Agronomy, Faculty of Agrarian and Forestry Sciences, Catholic University of Maule, Casilla 7-D, Curicó 3349001, Chile; 3 Centre for Pesticide Suicide Prevention, University/British Heart Foundation Centre for Cardiovascular Science, University of Edinburgh, Edinburgh EH16 4TJ, UK; 4 International Rice Research Institute, Makati 1226, Philippines; 5 School of Environmental Science and Management, University of the Philippines, Los Baños 4030, Philippines; 6 Association of Fruit and Vegetable Growers of Almeria (COEXPHAL), Carretera de Ronda 11, 04004 Almeria, Spain; 7 Department of Zoology, Ecology and Plant Science, University College Cork, Butler Building, Distillery Fields, North Mall, T23 N73K Cork, Ireland

**Keywords:** brown planthopper, fertilizer, herbivory tolerance, heterobeltiosis, heterosis, host plant resistance, plant physiology, rice herbivores, whitebacked planthopper, yellow stemborer

## Abstract

**Simple Summary:**

Hybrid rice often has higher yields than comparable inbred varieties. However, hybrids are sometimes more susceptible to insect herbivores. Outbreeding can improve herbivore resistance in hybrids compared to one (a condition called heterosis) or both (called heterobeltiosis) of their parental lines. The frequency of heterosis for resistance has not been assessed under varying soil nitrogen conditions. Nitrogen is predicted to reduce a plant’s ability to resist herbivores but increases its ability to compensate for damage, known as tolerance. We examined the resistance and tolerance of eight hybrids and their parental lines to herbivores by exposing plants to the brown planthopper, whitebacked planthopper or yellow stemborer and observing herbivore fitness responses (i.e., resistance) and herbivore-induced changes to plant biomass (i.e., tolerance). There were no consistent trends in relative resistance or tolerance to the herbivores across plant types; however, improved resistance and tolerance were frequently associated with the male parent. Nitrogen reduced resistance and generally increased tolerance to herbivores irrespective of plant type. Across the eight hybrids, relative resistance and relative tolerance were not determined by heterosis or heterobeltiosis. Our results highlight the difficulties in predicting the outcomes of crossing to achieve relatively resistant hybrids.

**Abstract:**

Hybrid rice results from crossing a male-sterile line (the A line) with a pollen doner (the restorer or R line). In 3-line hybrid breeding systems, a fertile B line is also required to maintain A line populations. Heterosis is defined as a condition of traits whereby the hybrid exceeds the average of the parental lines. Heterobeltiosis is where the hybrid exceeds both parents. Hybrid rice may display heterosis/heterobeltiosis for growth, yield and resistance to herbivores, among other traits. In a greenhouse experiment, we assessed the frequency of heterosis for resistance to the brown planthopper (*Nilaparvata lugans* (BPH)), whitebacked planthopper (*Sogatella furcifera* (WBPH)) and yellow stemborer (*Scirpophaga incertulas* (YSB)) in eight hybrids under varying soil nitrogen conditions. We also assessed plant biomass losses due to herbivore feeding as an approximation of tolerance (the plant’s capacity to compensate for damage). Nitrogen reduced resistance to all three herbivores but was also associated with tolerance to WBPH and YSB based on improved plant survival, growth and/or yields. Plant biomass losses per unit weight of WBPH also declined under high nitrogen conditions for a number of hybrids, and there were several cases of overcompensation in rice for attacks by this herbivore. There was one case of nitrogen-related tolerance to BPH (increased grain yield) for a hybrid line with relatively high resistance, likely due to quantitative traits. Heterosis and heterobeltiosis were not essential to produce relatively high herbivore resistance or tolerance across hybrids.

## 1. Introduction

Hybrid rice is grown extensively in some parts of South and Southeast Asia [1,2]. In China, the total area planted with hybrid rice rapidly increased after its initial introduction in the late 1970s, with some estimates that 50% (15 million hectares) of Chinese rice production currently relies on hybrid varieties [2,3]. More recently, hybrid rice production has spread to India, Bangladesh and Vietnam, where the rice acreage under hybrids increased dramatically in the early 2000s [1,4]. Several Asian countries (e.g., India, Myanmar, Indonesia and the Philippines) currently promote hybrid rice breeding as one of several strategies to increase national food production [1,3,4]. Hybrid varieties are produced by crossing male-sterile rice plants with fertility restorers (the pollen donors) [5,6]. The resulting hybrids produce both fertile and infertile grains that are unsuitable for seed harvesting, thereby promoting the commercialization of hybrid varieties. Hybrid varieties have generally higher (up to 15%) yields than inbred rice varieties, and seed quality is generally better, thereby avoiding seed-borne diseases and reducing seed contamination with weeds [3,7,8]. However, hybrids—particularly early-generation hybrids—have often been associated with high damage from insect herbivores and diseases [1,9,10]. The careful selection of breeding lines can reduce herbivore damage to hybrids [11]; however, susceptible hybrids are still widely available [12,13,14].

Based on a review of the literature, Horgan and Crisol (2013) [1] distinguished three main factors underlying herbivore damage to hybrids compared to inbred rice. (1) At field scales, hybrids may be more vulnerable to insect herbivores because of associated higher fertilizer applications to achieve yield potentials and higher pesticide use by farmers to protect their investments in relatively expensive rice seed. These practices increase the attractiveness of hybrids and the growth of pest populations by enhancing host plant quality (i.e., fertilizers and some resurgence pesticides) and reducing the diversity and abundance of natural enemies (i.e., resurgence pesticides) [1,15,16]. (2) Some hybrid varieties have relatively low anti-herbivore resistance (the ability to defend against herbivore oviposition (antixenosis) and growth and development (antibiosis)) because of relatively fast hybrid growth rates (e.g., planthoppers and leafhoppers), efficient assimilation of nitrogen by the plants (e.g., planthoppers, leafhoppers and stemborers), thick stems (e.g., stemborers) and high leaf and shoot biomass (e.g., leaffolders, stemborers and other caterpillars) [1,11,17,18]. There is also evidence that some hybrids may be ‘hyper-susceptible’ to planthopper damage, possibly associated with the female cytoplasm (i.e., from the male-sterile parent) [10,19,20]. (3) However, hybrids can also have a higher tolerance (the ability to compensate for damage and avoid yield losses) to herbivores because of their high growth rates and biomass accumulation, particularly under high resource (light, water, space and soil nutrients) availability [1]. For example, recent studies have shown that hybrids are more tolerant to herbivore damage compared to parental lines [18] and other inbred lines [21]. As such, the interactions between hybrid physiology and resource availability determine the nature of hybrid responses to insect herbivores relative to inbred varieties.

Hybrid physiology is largely governed by heterosis for growth, maintenance, reproduction and defense traits. Heterosis is defined as a condition of traits where the hybrid’s performance exceeds that of the average performance of its parental lines as a consequence of outbreeding. Hybrid performance exceeding that of both parents is known as heterobeltiosis [5,6]. Heterosis can be enhanced by increasing the genetic distance between parental lines [22,23]; this will also limit the movement of certain pests, such as planthoppers, between fields with different hybrid rice varieties [1,24]. Breeders can further avoid herbivore-susceptible parental lines and promote specific combinations of parental lines that achieve heterobeltiosis for herbivore resistance [11,25,26]. However, there are limitations on the choice of parents for hybrid breeding programs, as not all lines can restore fertility [5,6]. To overcome these limitations, breeders have used marker-assisted breeding to transfer genes for planthopper and gall midge resistance from traditional rice varieties and landraces (mainly from South Asia) or wild rice species to parental lines (either the male-sterile or restorer lines) [27,28,29,30,31,32]. However, varietal resistance is compromised under conditions of high fertilizer rates and by certain resurgence pesticides and is vulnerable to pest adaptation to the genes (known as virulence adaptation) [16]. For example, planthoppers have been noted to adapt to deployed resistance in less than 15 generations (about two–three rice seasons in tropical climates) [33], but durability may increase where the varieties have further background quantitative resistance or tolerance [33,34,35]. Tolerance places no selection pressures on herbivores and there is consequently no herbivore adaptation. Furthermore, tolerance to certain pests and diseases can be enhanced under high fertilizer conditions [18]. Despite its importance, only a few studies have examined heterosis for resistance to rice herbivores in hybrid rice [11,25,26]. This is partly because parental lines are generally restricted as intellectual property for commercial use. Furthermore, we know of no study that assesses aspects of herbivory tolerance across any range of hybrids and their parental lines or under varying fertilizer rates.

In this study, we examine aspects of heterosis for resistance under varying soil nitrogen conditions across a range of hybrids and their associated parental lines from the 3-line hybrid rice breeding program at the International Rice Research Institute (IRRI) in the Philippines. We also quantified damage to the rice plants as lost biomass and related this to herbivore pressures as an indicator of plant tolerance. We used three herbivores, the brown planthopper (*Nilaparvata lugens* Stål: BPH), whitebacked planthopper (*Sogatella furcifera* (Horvath): WBPH) and yellow stemborer (*Scirpophaga incertulas* (Walker): YSB) in our greenhouse experiments. We hypothesized that resistance would decline under high soil nitrogen conditions but that tolerance would increase. Previous studies have indicated that high nitrogen conditions will only enhance tolerance to BPH in lines with at least moderate resistance [21]; therefore, we predicted that only tolerance to WBPH and YSB would be enhanced under high nitrogen conditions, with a greater number of plants displaying aspects of tolerance to these two species as the nitrogen amount increased. We also predicted that, compared to their parental lines, the hybrids would show a greater enhancement of tolerance in response to soil nitrogen because of associated heterosis for growth rates and biomass accumulation (i.e., better nitrogen assimilation). We predicted that heterosis for increased resistance and tolerance would be independent in most cases, with improved resistance arising due to heterosis for defense responses, whereas faster growth rates and better nitrogen assimilation underlie both tolerance and susceptibility (i.e., heterosis for resistance and heterosis for tolerance would not necessarily co-occur across hybrids). Based on our results, we make recommendations for future research into hybrid rice–herbivore interactions and for the field management of hybrid rice.

## 2. Materials and Methods

### 2.1. Plant Materials

We used a total of 30 rice lines grown in a greenhouse at IRRI (the Philippines) to represent eight hybrid breeding groups (hybrids and parental lines). The 3-line hybrid breeding system involves a cytoplasmic male-sterile line or CMS line (also called the A line), a maintainer line (also called the B line) and a restorer line (the R line). Hybrid seed is produced by crossing the A line and R line. The B line is required to maintain A line seed. The A and B lines share the same nuclear genome but have distinct cytoplasmic genomes that bestow sterility to the A line [5,25]. We arbitrarily selected the eight hybrids (Table S1) and their associated parental lines from the IRRI breeding program, with no a priori considerations except that a sufficient number of seeds should be available for the experiments. Among the hybrids that we used, three (IR82391H, IR84714H and IR85471H) shared the same R line (IR 60819-34-2R). Therefore, in total, we used 30 separate rice lines (genotypes) in the experiments. These same varieties have been used in previous studies, including as part of a wider study on the frequency of heterosis for resistance within the IRRI hybrid breeding program [11] and in field trials to assess plant development and yields and the field occurrence of planthoppers and stemborers [11,18]. The main results from these studies with respect to each of the eight hybrids are summarized in Table S1.

The experiments were conducted using potted plants under greenhouse conditions (greenhouse and cage conditions, i.e., light, temperature and humidity are detailed in a related paper [36]). Greenhouse experiments are particularly useful for understanding planthopper–rice interactions because they avoid the normally high predation rates that occur in field plots that would otherwise obscure the results [11]. Rice seed was initially sown to saturated, homogenized paddy soil in plastic basins (25 cm × 30 cm × 50 cm, H × W × L) and, after 10 days, the seedlings were individually transplanted to number 6 pots (15 cm × 15 cm, H × D) with saturated paddy soil. The pots were placed in flooded trays to avoid heat stress and prevent interference from ants. The pots were watered daily and received no pesticide treatments. Half of the pots received fertilizer equivalent to 150 Kg N ha^−1^ by treating the soil with 0.124 g of ammonium sulphate one day before transplanting and a further 0.062 g at 3 days before infestation. This amount was based on the estimated weight of topsoil per hectare (2000 tonnes), the weight of soil in each pot (1.3 Kg pot^−1^) and the percentage of nitrogen contained in ammonium sulphate (21%).

### 2.2. Insect Herbivores

We used BPH, WBPH and YSB in our experiments. The two planthoppers are regarded among the most damaging pests of rice throughout Asia [16]. Both species have become prevalent in hybrid rice and are associated with excessive fertilizer and pesticide use. Outbreaks of WBPH in particular have increased in recent years and are often associated with hybrid rice [1,10]. Both planthopper species normally attack early-stage rice plants (tillering stage) where they feed and lay eggs. The nymphs pass through five instars and, under favorable conditions, can have several generations per crop, with brachypterous adults more common during early generations and macropterous adults emerging as the rice nears maturity or on low-quality hosts [37]. Heavy infestations produce patches (known as hopperburn) of dead plants in rice fields [16]. 

We used planthoppers from colonies initiated five years prior to conducting the experiments, each with >500 individuals collected using sweep nets in rice paddies from Laguna, the Philippines. We made periodic introgressions of wild-caught planthoppers, collected from the same site, over the five years. The colonies were maintained in wire-mesh cages of 120 cm × 60 cm × 60 cm (H × W × L) in a shaded greenhouse. The planthoppers were continuously fed on a highly susceptible rice variety (TN1) of >30 days after sowing (DAS), with feeding plants changed every two weeks. Details of virulence adaptations among Laguna BPH and WBPH have been presented by Horgan et al. (2017) [38].

YSB is the main stemborer pest of rice in tropical Asia [39]. YSB predominantly attacks rice at the seedling or tillering stages. The adults deposit egg masses on the rice foliage. After emergence, the neonates tunnel into the tillers where they feed and develop [39]. Because YSB is difficult to maintain in laboratory colonies, we collected adults using sweep nets at dusk from rice fields in Laguna Province, 3–5 days before they were required for the experiments. The adults were placed in plastic cages (100 cm × 50 cm × 50 cm: H × W × L) with >30 DAS TN1 and allowed to mate and lay eggs. The egg masses were collected from the cages and monitored in Eppendorf tubes until the neonates emerged. The neonates were used in experiments within 1 h of egg hatch. 

### 2.3. Greenhouse Experiments

At 25 DAS, plants in number 6 pots were each covered with an acetate cage (123 × 12 cm, H × D) with a nylon mesh window (23 cm × 15 cm: H × W) and nylon top. The pots were arranged as a randomized replicated block design with six replicated blocks. Each block was located in a separate greenhouse compartment (separated by screened partitions). Each block consisted of 240 pots (30 genotypes [lines] × 4 treatments [BPH, WBPH, YSB, and non-infested controls] × 2 nitrogen levels [N0 = zero added nitrogen, N1 = 150 Kg N ha^−1^]). The pots were randomly positioned in each compartment (i.e., within replicated blocks). The entire experiment consisted of 1440 pots. The herbivore-treated plants were either infested at 25 DAS with six neonate YSB (<1 h) or at 30 DAS with either two recently emerged, gravid female BPH or two recently emerged, gravid female WBPH. The herbivores were inserted into each cage through a slit in the acetate. 

At 20 days after infestation (DAI), all planthoppers (BPH and WBPH) were removed from the plants using a vacuum sampler passed through the top of the cage (i.e., removing the top mesh). The cages were examined again after 7 and 10 days to collect any second-generation nymphs that developed from eggs already in the plants. These were added to the corresponding samples collected from each cage at 20 DAI. No further nymphs were observed after 30 DAI. The stemborers were allowed to develop until adult moths were noted in the cages. The adults were then removed daily, recording the date of emergence, until no new adults emerged. All collected insects were placed in glass test tubes. After removing all insects, the plants were allowed to continue developing until harvest (i.e., when 85% of the grain was mature). The plants were then destructively sampled by carefully saturating the soil and then removing soil from the roots under running water. The plants were each separated into roots, aboveground shoots and leaves and panicles. The panicles were further separated into filled and unfilled grains. The plant parts were placed individually in paper bags. 

After collection, the insects and harvested rice plants were immediately dried in forced draught ovens at 60 °C for one week (insects) or until a constant weight (plants). After drying, the insects were weighed (total weight per plant) and the developmental stages (planthoppers) and sex of adults (planthoppers and stemborers) were recorded. For planthoppers, we also noted the number of brachypterous adults and the proportion of adults that were female, and for stemborers, we recorded the dry weights of each adult moth. The dried plant parts were individually weighed, and the number of tillers and grains were counted. Results related to the same hybrid groups under low nitrogen conditions and based on a subset of the replicated blocks are presented as part of an experiment with a greater number of hybrids and their parental lines in a related paper [11].

### 2.4. Data Analyses

This study addresses the frequency of heterosis and heterobeltiosis for resistance and tolerance to BPH, WBPH and YSB by comparing insect and plant responses between hybrids and their respective parental lines. To convey the main results from these analyses and avoid extensive reporting, we have included the full results as a series of Supplementary Tables and summarize the main results related to each hybrid variety in the body of the text.

We used univariate general linear models (GLM) to compare the biomass of grain, shoots and roots across plant types within each hybrid breeding group. We also compared the differences in biomass of each plant component under low and high nitrogen conditions to examine relative efficiency in nitrogen assimilation during the experiments. To compare biomass responses in all three herbivores, we conducted initial analyses using three-way GLMs with insect species, fertilizer and plant type as the main factors and removing the effects of blocks. Pairwise comparisons were made using Tukey’s LSD tests. All biomass data were log(x ± 1) transformed before analyses, and residuals were plotted after analyses to test whether the conditions for normality and homogeneity were met. Because of large differences in the final biomass attained by the species (see below), as well as several cases with significant three-way interactions, we subsequently divided our results and analyses to represent distinct experiments for each herbivore. This facilitated the interpretation of the results. The separate analyses for each species are described in the following paragraphs.

Fitness parameters (i.e., the proportions of adults that were female, the total number of individuals per plant and the total dry weight of insects at the end of the experiment) for BPH, WBPH and YSB and plant condition at the end of the experiments (i.e., number of tillers, grain, aboveground and root weights) were analyzed using univariate GLMs with accession and nitrogen levels as the main factors. Data on the numbers of YSB that were female and the relative development times and weights of male and female YSB were analyzed using univariate GLMs with three main factors (accession, nitrogen and sex). Planthopper development (relative proportions at each development stage and proportions of males and females that were brachypterous) were analyzed using multivariate GLMs. For WBPH, only the proportions of brachypterous females were analyzed (using univariate GLMs) because there were very few brachypterous males. Block (1–6) was initially included in the analyses but was removed because there were no significant effects. The same analyses (univariate or multivariate GLMs as above) were conducted with the hybrids alone to determine whether heterosis was related to relative resistance across hybrids. We identified cases of heterosis for resistance based on insect numbers and/or insect biomass (i.e., where herbivore numbers/biomass was significantly lower on hybrids compared to one parental line), heterobeltiosis for resistance (i.e., where numbers/biomass were significantly lower on hybrids compared to both parental lines—including either the A line or B line) and heterobeltiosis for susceptibility (i.e., where numbers/biomass were significantly higher on hybrids compared to both parental lines—including either the A line or B line) using univariate GLMs. Differences in the responses of herbivores on A and B lines highlight possible influences of cytoplasmic genes (A lines) and nuclear genes. Proportional data were arcsine-transformed, and insect numbers and biomass were log(x ± 1)-transformed before analyses. Pairwise comparisons were made using Tukey’s LSD tests. 

A number of authors have applied a range of different metrics to indicate comparative levels of tolerance across rice plants. These include relative changes in plant color or chlorophyll content, changes to tillering responses and plant weight (particularly losses in grain weight), plant mortality or the appearance of herbivore damage (particularly for stemborers) under a gradient of herbivore pressures [39,40,41,42,43]. However, ensuring standard pressure from herbivores across test plants is difficult to achieve because plants also express different levels of resistance and because intraspecific competition rapidly increases as herbivore densities increase, thereby confounding the relative impacts of herbivore densities on the plants. For these reasons, we used plant biomass loss and the loss of biomass per unit biomass of insects as indicators of relative herbivore-induced impacts on the test plants (that include aspects of tolerance) but without achieving the necessary standardized herbivore feeding pressures to properly estimate relative plant tolerances. Furthermore, our measure includes plant recovery as a component of tolerance, because the plants were allowed to continue developing after the herbivores were removed. This was carried out to determine the effects of herbivory on the redistribution of resources among plant tissues and has been reported for hybrids in general in a related paper [18]. In the Results and Discussion, we sometimes refer to these ‘aspects of tolerance’ as ‘tolerance’.

We calculated absolute and proportional plant weight losses due to insect herbivory by comparing the corresponding control (i.e., non-infested) and infested plants in each replicated block. Because A lines produce no filled grain, we standardized plant weights across all plant types by estimating the costs in aboveground biomass to produce 1 g of seed by subtracting the aboveground biomass of the B lines from their corresponding A lines and dividing by the biomass of the B line grain. A single biomass equivalent was determined for each infested and control plant by adding the corresponding biomass of shoots and roots. We focused on herbivore biomass-induced changes in plant weights as our main measure of impacts by calculating the absolute biomass loss in plants (units = g) per unit weight (=1 mg) of insect biomass. Absolute and proportional biomass losses and biomass loss per unit weight of herbivores were analyzed using univariate GLMs with accession and nitrogen as the main factors and the control plant weight equivalents as covariates. The covariates were removed where they had no significant effects. The analyses were repeated using only the hybrid lines. We identified cases of heterosis for herbivore-induced impacts, heterobeltiosis for reduced herbivore-induced impacts (approximating relative tolerance) and heterobeltiosis for increased herbivore-induced impacts (approximating relative non-tolerance) using univariate GLMs. Pairwise comparisons were made using Tukey’s LSD tests. Residuals were plotted after each analysis to check that they were normal and homogenous.

## 3. Results

### 3.1. Nitrogen Effects on Plant Biomass

Nitrogen consistently increased the total biomass of rice plants and the aboveground shoot biomass. For most breeding groups, nitrogen also increased yield and root biomass (Table S2). A lines produced panicles and grain, but the grain was sterile. The lack of grain-filling resulted in larger A line shoot biomass in five of the eight breeding groups (Table S2). With the exception of the IR80637H group, hybrid lines tended to have significantly larger shoots than the B or R lines (Table S2). B lines were generally smaller than the other plant types, including smaller roots. Plant type affected nitrogen conversion to grain biomass, with hybrid lines and B lines increasing their grain yields more than the R lines after the addition of nitrogen (Table S3). Changes in shoot biomass due to nitrogen were always greater than for grain biomass but were more consistent between plant types (i.e., there were no or low significant nitrogen effects on shoot biomass across plant types: Table S3).

### 3.2. Comparative Effects of Nitrogen on Herbivore Biomass

**Figure 1:**
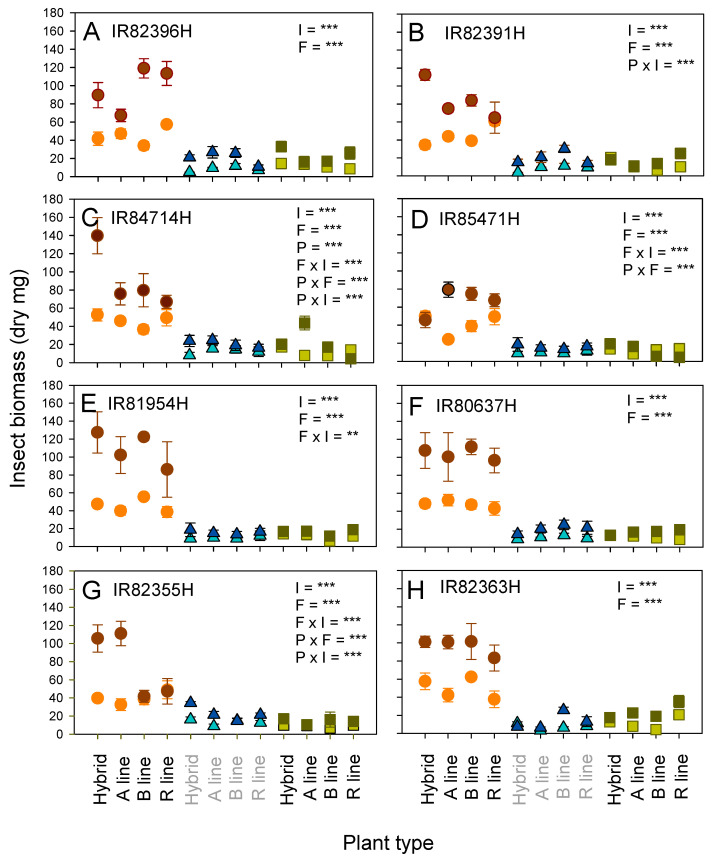
Biomass of brown planthopper (brown circles), whitebacked planthopper (blue triangles), and yellow stemborer (green squares) on eight hybrid lines and their associated parental lines under low (light colored symbol) and high (dark colored symbols) nitrogen conditions. The results for the (**A**) IR82396H, (**B**) IR82391H, (**C**) IR84714H, (**D**) IR85471H, (**E**) IR81954H, (**F**) IR80637H, (**G**) IR82385H and (**H**) IR82363H hybrids and their parental lines are shown. Means and standard errors are indicated (N = 6). The results of univariate GLMs are indicated for main factors (I = insect species, F = fertilizer level, P = plant type) and their significant two-way interactions are indicated together with significance levels denoted as ** = *p* ≤ 0.01 and *** = *p* ≤ 0.005. The full results of GLMs are presented in Table S4. Separate results for each insect species are presented in Section 3.3.

BPH attained a greater total herbivore biomass than WBPH and YSB for all eight hybrid breeding groups (Figure 1). Herbivore biomass was also greatest on plants that received added nitrogen (Figure 1). Plant type affected final herbivore biomass only in the IR84714H group (Figure 1C), with significantly more biomass on the hybrid than on the B and R lines. However, there were significant three-way interactions for several of the hybrid breeding groups (IR82391H, IR84714H, IR85471H and IR82385H), and IR82391H, IR84714H, IR85471H, IR81954H and IR82385H had significant interactions between plant type and/or fertilizer level and insect species. Interactions between fertilizer level and insect species were largely due to the higher biomass of BPH on some plants that received nitrogenous fertilizer compared to WBPH and YSB on plants under both nitrogen regimes (Figure 1).

### 3.3. Herbivore–Rice Interactions under Low and High Nitrogen Conditions

#### 3.3.1. Brown Planthopper

**Figure 2:**
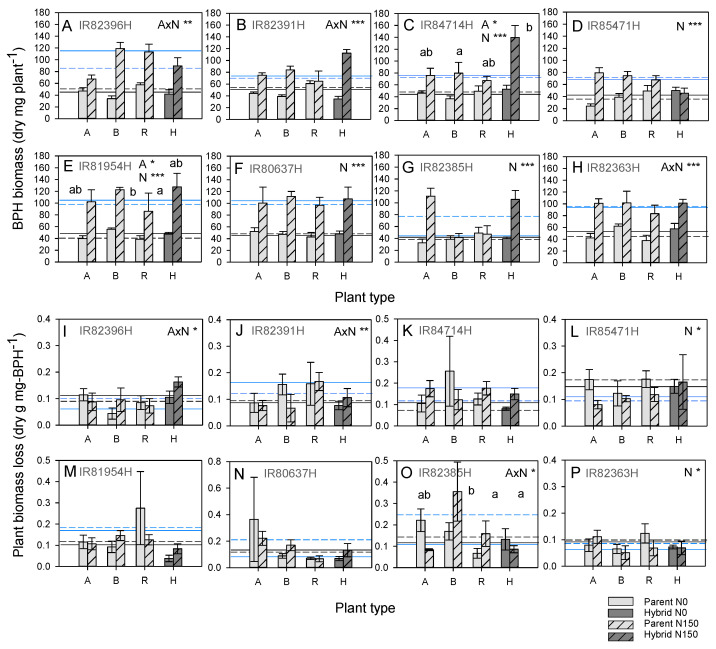
(**A**–**H**) Total dry weight (biomass) of brown planthopper and (**I**–**P**) plant biomass loss per unit planthopper weight on eight hybrids (H on *x*-axes) and their associated parental lines (A line, B line and R line, A, B and R on the *x*-axes, respectively) in a greenhouse experiment. The results are presented for the (**A**,**I**) IR82396H group, (**B**,**J**) IR82391H group, (**C**,**K**) IR84714H group, (**D**,**L**) IR85471H group, (**E**,**M**) IR81954H group, (**F**,**N**) IR80637H group, (**G**,**O**) IR82385H group and (**H**,**P**) IR82363H group. The results of univariate GLMs are indicated with each graph as A = accession, N = nitrogen and A × N = accession × nitrogen interaction with * = *p* ≤ 0.05, ** = *p* ≤ 0.01 and *** = *p* ≤ 0.005. Lowercase letters indicate homogenous plant types (Tukey *p* > 0.05). Means and standard errors (N = 6) are shown. Average parameter values for A lines (dashed lines) and B lines (solid lines) are indicated for plants grown under low (black) and high (blue) nitrogen conditions. Cases of heterosis are indicated in Table 1. See Tables S5 and S6 for details of other BPH fitness parameters and plant responses.

Nitrogen increased aspects of BPH fitness on all lines: nymph development rates increased under high nitrogen conditions as indicated by lower proportions of early instars (IR82396H, IR85471H and IR82385H) and higher proportions of adults (IR84714H and IR85471H) (Table S5). Nitrogen increased total BPH biomass (all groups). There were significant interactions between nitrogen and plant type for BPH biomass in IR82396H, IR82391H, IR82385H and IR82363H; however, in all of these cases, BPH biomass increased significantly on hybrids whereas it declined or remained the same on one or other parental line (Figure 2). BPH population sizes were larger under high nitrogen conditions with statistically significant increases in IR82396H, IR82391H, IR80637H and IR82385H; significant interactions between nitrogen and plant type in IR82391H and IR82385H were due to increased BPH numbers on hybrids compared to one or other parental line (Table S5). The proportions of brachypterous adults were often higher on hybrid lines (IR84714H, IR85471H and IR80637H) (Table S5).

Across the hybrids, IR85471H was the most resistant, with IR84714H, IR81954H and IR82363H most susceptible to BPH (Table 1). The relative resistance of IR85471H was associated with relatively resistant A, B and R lines without heterosis or heterobeltiosis. Susceptibility in IR81954H was associated with heterobeltiosis for higher BPH numbers based on the A line and heterosis with the B line, despite a relatively resistant R line. Heterosis was apparent for biomass and planthopper numbers in five cases (three with A lines and four with B lines; see Table S7 for the other response parameters).

Nitrogen increased aboveground and root biomass in IR85471H, IR81954H and IR80637H despite BPH infestation; however, grain biomass increased under high nitrogen conditions only in IR85471H (Table S6). Furthermore, the absolute and percentage weight lost due to BPH declined under nitrogen in IR85471H but not in the associated A, B and R lines (Table S6). At the end of the experiment, hybrid lines were often the largest plants (IR82391H, IR84714H, IR85471H, IR81954H and IR80637H) and maintainers were the smallest (IR82396H, IR82391H, IR84714H, IR85471H, IR81954H, IR80637H and IR82385H) (Table S6). Plant weight loss was lowest in hybrids in only two cases (IR81954H and IR80637H) (Table S6). Nitrogen was associated with increased plant biomass loss in four cases but depended on plant type in three of these cases (IR82396H, IR82391H and IR82385H) (Figure 2I–P); nitrogen increased damage in IR82396H and IR82391H per mg BPH but reduced damage to IR82385H (heterosis with B line) (Figure 2). 

Across the hybrids, IR85471H had the highest per plant yields (Table S8); this was associated with the lowest weight losses—although differences between hybrids were not significant (Table 1). There were five cases of heterosis (four with A lines, four with B lines), four of which were associated with relatively low herbivore-induced losses in the R lines (Table 1 and see Table S8 for other response parameters).

#### 3.3.2. Whitebacked Planthopper

**Figure 3:**
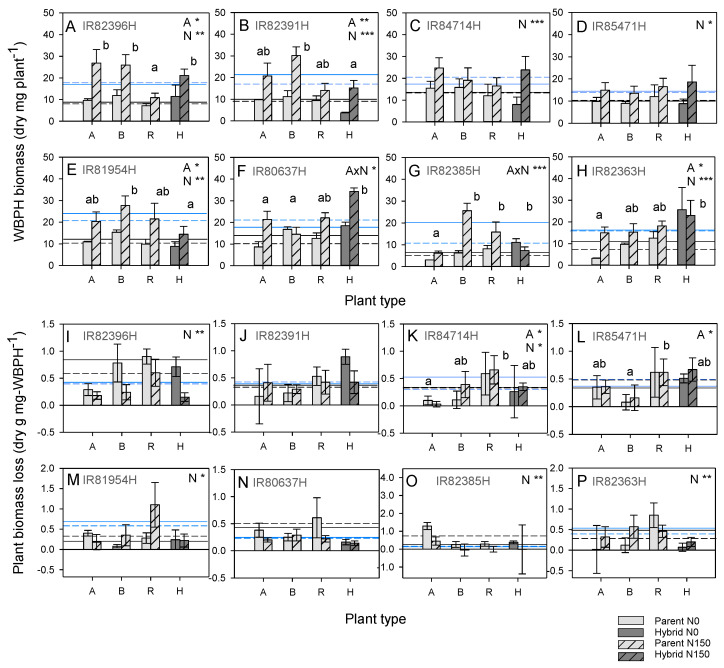
(**A**–**H**) Total dry weight (biomass) of whitebacked planthopper and (**I**–**P**) plant biomass loss per unit planthopper weight on eight hybrids (H on *x*-axes) and their associated parental lines (A line, B line and R line, A, B and R on the *x*-axes, respectively) in a greenhouse experiment. The results are presented for the (**A**,**I**) IR82396H group, (**B**,**J**) IR82391H group, (**C**,**K**) IR84714H group, (**D**,**L**) IR85471H group, (**E**,**M**) IR81954H group, (**F**,**N**) IR80637H group, (**G**,**O**) IR82385H group and (**H**,**P**) IR82363H group. The results of univariate GLMs are indicated with each graph as A = accession, N = nitrogen and A × N = accession × nitrogen interaction with * = *p* ≤ 0.05, ** = *p* ≤ 0.01 and *** = *p* ≤ 0.005. Lowercase letters indicate homogenous plant types (Tukey *p* > 0.05). Means and standard errors (N = 6) are shown. Average parameter values of A lines (dashed lines) and B lines (solid lines) are indicated for plants grown under low (black) and high (blue) nitrogen conditions. Cases of heterosis are indicated in Table 2. See Tables S9 and S10 for details of other WBPH fitness parameters and plant responses.

Nitrogen increased aspects of WBPH fitness on all lines. The proportions of adults were significantly higher under high nitrogen conditions in six of eight cases (IR82396H, IR82391H, IR84714H, IR81954H, IR82385H and IR82363H); significant nitrogen interactions with plant type for IR82391H and IR84714H were due to lower numbers of adults on B lines and hybrids, respectively (Table S9). Population sizes increased under high nitrogen conditions in four cases (IR82396H, IR82391H, IR80637H and IR82385H) with a significant interaction for IR82396H because of lower numbers on the hybrid under higher nitrogen conditions (Table S9). Nitrogen increased total WBPH biomass in all cases, with a significant interaction only in IR80637H and IR82385H because of no changes in WBPH biomass on the B lines and lower weights on the hybrids under high nitrogen conditions, respectively (Figure 3). Hybrids were often the most resistant plants, as indicated by low proportions of adults (IR82396H and IR80637H) (Table S9), low proportions of brachypterous females (IR82396H) (Table S9) and low WBPH biomass (IR82391H and IR81954H) at the end of the experiment (Table S9, Figure 3).

Across the hybrids, IR82391H, IR84714H, IR85471H, IR81954H and IR82385H were significantly more resistant than IR80637H (based on WBPH biomass) (Table 2). There were five cases of heterosis for biomass (three for A lines; four for B lines) (Table 2). Heterosis did not determine relative resistance across the hybrids. One hybrid, IR82396H, was associated with a relatively resistant R line (Table 2).

Nitrogen increased shoot and/or root weights in all cases despite WBPH infestations. Grain weights also increased under high nitrogen conditions in IR85471H, IR82385H and IR82363H. There was only one significant interaction: for IR82391H, nitrogen had no effect on shoot biomass on A and B lines and reduced root biomass in the same lines (Table S10). A line plants had the highest root and shoot biomass whereas B and R lines generally had the lowest biomass (Table S10). Nitrogen was associated with higher absolute and/or proportional weight loss in IR82391H, IR84714H, IR85471H, IR81954H, IR80637H and IR82363H but lower weight loss in IR82385H. Weight loss was greatest in the restorer lines on IR82396H, IR84714H and IR85471H. There were no cases of heterosis or heterobeltiosis for biomass loss (absolute or proportional) (Table S10). Nitrogen was associated with higher plant biomass losses per mg WBPH in IR84714H and IR81954H but lower losses in IR82396H and IR82385H. There was no evidence of heterosis for plant biomass loss per mg WBPH for any hybrids.

Across the hybrids, nitrogen increased plant survival and all plant growth and biomass loss parameters (Table 2). Absolute biomass loss was lower in IR82363H than IR85471H, and proportional losses were higher in IR82363H than IR82396H, without any associated heterosis or heterobeltiosis for resistance (Table 2). 

#### 3.3.3. Yellow Stemborer

Nitrogen increased the number of emerging moths and/or total moth biomass in all cases, with significant effects in IR82396H, IR84714H, IR85471H, IR81954H, IR82385H and IR82363H and some exceptions in IR84714H (R line), IR85471H (B and R line) and IR81954H (A line and hybrid), resulting in significant nitrogen by plant type interactions (Figure 3, Table S13).

Across the hybrids, nitrogen reduced female development times, female weights, male weights and the total biomass of moths on the plants (Table S15). But there were no significant accession effects on moth fitness parameters despite cases of heterosis and heterobeltiosis for susceptibility (based on comparisons with the B line) in two of the breeding groups (Table 3 and Table S13).

**Figure 4:**
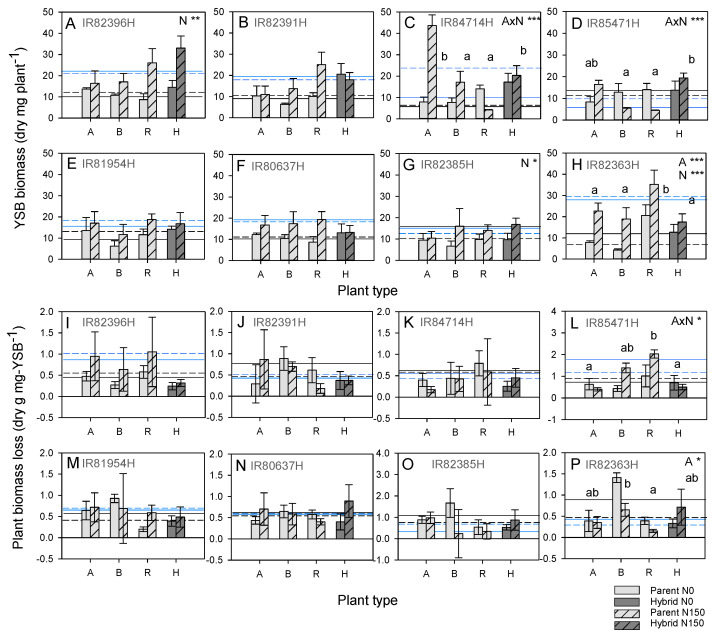
(**A**–**H**) Total dry weight (biomass) of yellow stemborer and (**I**–**P**) plant biomass loss per unit planthopper weight on eight hybrids (H on the *x*-axes) and their associated parental lines (A line, B line and R line, A, B and R on the *x*-axes, respectively) in a greenhouse experiment. The results are presented for the (**A**,**I**) IR82396H group, (**B**,**J**) IR82391H group, (**C**,**K**) IR84714H group, (**D**,**L**) IR85471H group, (**E**,**M**) IR81954H group, (**F**,**N**) IR80637H group, (**G**,**O**) IR82385H group and (**H**,**P**) IR82363H group. The results of univariate GLMs are indicated with each graph as A = accession, N = nitrogen and A × N = accession × nitrogen interaction with * = *p* ≤ 0.05, ** = *p* ≤ 0.01 and *** = *p* ≤ 0.005. Lowercase letters indicate homogenous plant types (Tukey *p* > 0.05). Means and standard errors (N = 2–6, see Table S13) are shown. Average parameter values of A lines (dashed lines) and B lines (solid lines) are indicated for plants grown under low (black) and high (blue) nitrogen conditions. Cases of heterosis are indicated in Table 3. See Tables S13 and S14 for details of other WBPH fitness parameters and plant responses.

Nitrogen increased root (IR84714H and IR82363H), grain (IR84714H, IR85471H, IR81954H and IR82363H) and/or shoot biomass (significant in IR82396H, IR84714H, IR85471H, IR81954H, IR80637H and IR82363H) and increased tiller numbers in IR82396H, IR84714H and IR82363H despite stemborer infestations (Table S14). Significant plant-type effects were due to larger roots or shoots in A lines (IR82391H, IR85471H, IR81954H, IR82385H and IR82363H) (Table S14). Nitrogen increased the estimated proportional and/or absolute biomass loss in all cases (not significant in IR82391H) (Figure 4, Table S14). Hybrids had the greatest losses in biomass of all plant types in IR84714H under high nitrogen conditions (heterobeltiosis for susceptibility with the B line) and had high biomass losses under low nitrogen conditions (heterosis with B line) (Table S14). Nitrogen had no significant effects on plant biomass loss per mg of YSB (Figure 4I–P).

Across the hybrids, nitrogen increased all plant growth parameters, as well as proportional and absolute biomass losses (Table 3, Table S14). Furthermore, all plants survived until harvest under high nitrogen conditions (Table S14). IR81954H had less biomass loss than IR85471H and consequently higher grain yields (Table S14), without associated heterosis or heterobeltiosis for stemborer resistance but with apparent heterosis for biomass losses per mg YSB (Table 3).

## 4. Discussion

### 4.1. Nitrogen Effects on Rice Biomass

Nitrogen increases rice plant biomass and yields [44,45,46,47]. In our experiments, biomass conversion to grain under added nitrogen was generally more efficient in B lines and hybrids than in the associated R lines. Biomass conversion to grain for hybrids may have been underestimated in our experiments because hybrids are affected by restricted soil volumes and acetate cages to a greater extent than B lines [36]; therefore, conversion is expected to be even greater for hybrids compared to the inbred lines under field conditions. In a related field study using the same eight hybrid breeding groups, the hybrids often had higher yields than B and R lines under similar high fertilizer levels (e.g., combined averages: hybrids = 7.6 t ha^−1^; R lines = 5.7 t ha^−1^; B lines = 5.3 t ha^−1^ [18]), with three hybrids showing heterobeltiosis for higher yields and a further three showing heterosis [11] (Table S1). Furthermore, during much of the growing phase, the hybrids had generally faster growth rates in the field compared with inbred parents, and, until about maturity, this was mainly associated with the production of larger tillers [18]. In our experiments, we confined our herbivore infestations to the early tillering stages of the plants when growth rates likely differed across plant types; in particular, the B lines attained a generally smaller size by the end of the experiment (Table S3) and even before maximum tillering (i.e., based on similar early tillering greenhouse plants reported by Horgan et al. (2024)) [11].

### 4.2. Nitrogen Effects on Rice Resistance to Herbivores

A large number of studies have shown how nitrogenous fertilizers reduce the resistance of rice plants by accelerating the development and growth of insect herbivores. Furthermore, greenhouse choice bioassays and field studies have shown that herbivores, including planthoppers and stemborers, are more attracted to plants and rice plots under high fertilizer regimes [18,33,44,45,46,48,49,50,51]. At larger scales, the higher use of fertilizers on hybrid rice compared to inbred varieties has been associated with relatively high levels of visually apparent damage from stemborers known as whitehead [1]. Our results further indicate the effects of nitrogen in accelerating development, reducing the production of macropterous adults, increasing population size and the proportion of surviving females, and increasing the overall biomass of BPH and WBPH on infested rice plants (Figure 1, Figure 2 and Figure 3). Nitrogen also increased the survival of YSB larvae and the final biomass of YSB on infested plants (Figure 4). Our results also clearly indicate the relatively greater potential of BPH populations to respond to nitrogenous fertilizers compared to WBPH and YSB (Figure 1). BPH in particular has been noted in previous studies to respond strongly to nitrogenous fertilizers [52,53,54]. In our experiment, BPH biomass was often two to six times higher on equivalent plants compared to WBPH and YSB under the high nitrogen regime but was more similar across the three species under low nitrogen conditions in a number of breeding groups (Figure 1). This greater potential of BPH can partly be explained by continued egg laying (and possibly feeding) activity during the nighttime compared to WBPH and an apparent ability to preempt nutrient resources before they are converted to plant tissue [18,55]. This has been associated with a strong attraction to the amino acid asparagine, which is more prevalent in chemically fertilized rice plants (compared to plants subjected to organic fertilizers) [56,57] and efficient nitrogen metabolism linked to gut-dwelling endosymbionts [58,59]. These factors may explain why BPH-susceptible rice plants fail to compensate for BPH damage under increased nitrogenous fertilizer levels [21]. In comparison with BPH, the other two herbivores had relatively moderate biomass increases under high nitrogen conditions, and there were several cases of rice overcompensating for WBPH or YSB damage under both fertilizer regimes (indicated by negative plant biomass loss values in Figure 3 and Figure 4) but no cases with BPH (Figure 2).

### 4.3. Heterosis and the Relative Resistance of Hybrid Accessions

In agreement with previous studies [11,25,26] we observed a relatively low frequency of heterosis for resistance to herbivores among 3-line hybrids from the IRRI breeding program. Only one of the R lines (IR46) is recognized as possessing a major BPH resistance gene; however, this *Bph1* gene is known to be ineffective against BPH throughout Asia [35,38]. Nevertheless, despite the lack of resistance genes, there were significant differences in the total biomass of both BPH and WBPH across hybrids: IR85471H was more resistant than IR84714H, IR81954H and IR82363H to BPH (Table 1), and IR82391H, IR84714H, IR85471H, IR81954H and IR82385H were more resistant than IR80637H to WBPH (Table 2). By comparing planthopper responses to plant types within the breeding groups, the relatively higher resistance to BPH in IR85471H can be associated with relatively high resistance across all of its parental lines (i.e., there was no evidence of heterosis or heterobeltiosis). Meanwhile, the relatively susceptible lines were associated with heterosis, and IR81954H showed heterobeltiosis for higher numbers of BPH (Table 1), with susceptibility often greatest among their A and B lines (i.e., the female parent). In a previous study that compared BPH responses to several hybrids with the same female parents, susceptibility in IR81954H was attributed to a highly susceptible IR703069A line [11]. The result was confirmed in the present study under high nitrogen conditions. In IR81954H and IR82363H, susceptibility was likely associated with the nuclear genome of the female parent (i.e., the A and B lines), with the male parent (R line) associated with an increase in hybrid resistance relative to the female lines in each case (Table 1). There was no evidence that A lines were consistently more susceptible to any herbivores than the B lines, thereby indicating that susceptibility was not generally associated with the A line cytoplasmic genome.

In the case of WBPH, only IR82396H showed heterobeltiosis for resistance based on brachypterous adults and the proportions of females emerging (Table S11); heterosis for increased resistance based on the number and biomass of WBPH was attributed to the R line. However, this hybrid was no more resistant to WBPH than any of the other seven hybrids (Table 2). Furthermore, there was no association between heterosis and the relative resistance to WBPH across hybrids (Table 2). Similarly, for YSB, two cases of heterobeltiosis for susceptibility were not reflected in significantly more susceptible hybrids compared to cases without heterobeltiosis (Table 3). In a previous study, we noted that susceptibility to YSB and other Lepidoptera was associated with heterosis for plant biomass accumulation and crop duration [11]. Because we limited our exposures to relatively young plants in the present study, we suggest that YSB survival and biomass were more directly related to relative susceptibilities based on the defense traits of the test plants. However, although we did identify some differences between YSB fitness on different plant types within hybrid groups, there were no consistent trends across the groups (Figure 4A–H). Our results with all three herbivores therefore suggest that heterosis or heterobeltiosis for resistance or susceptibility does not determine the relative resistance of hybrids from the same breeding program: crossing and resultant heterosis with a relatively resistant R line was beneficial but not essential to produce the most resistant hybrids in our study.

### 4.4. Nitrogen Effects on Herbivore-Induced Changes in Rice Biomass

We predicted that nitrogen would increase the tolerance of rice to WBPH and YSB, particularly for hybrid lines. Because none of the lines have recognized, effective BPH resistance genes, we expected that tolerance would decline under high nitrogen conditions for plants infested with BPH [11,18]. Nevertheless, we found that one of the hybrids, IR85471H, was more resistant to BPH because of comparatively good resistance among its parental lines. When infested with BPH, IR85471H was the only hybrid to increase grain yields (by ca 100%) under added nitrogen, and IR85471H hybrids had lower biomass losses (24%) compared to the losses of biomass (>45%) for the associated A, B and R lines (see significant nitrogen × plant type interaction for biomass loss in IR85471H: Table S6), indicating that the hybrid was relatively tolerant. These results therefore corroborate the recent findings associating moderate resistance with nitrogen-induced increases in plant tolerance to BPH [21]; however, unlike previous studies, in this case the resistance was likely associated with quantitative traits and not with any major resistance genes. Tolerance to BPH has been associated with nutrient translocation from the primary tiller to the main shoot in infested rice [60]. Our results with BPH-infested plants showed only one other case of improved tolerance in hybrids under high nitrogen conditions compared to the associated parental lines (i.e., the IR82385H group); however, the very high numbers of BPH on the hybrid under high nitrogen conditions suggest that this may be an artifact due to intense crowding and a large number of early instar nymphs. In four of the five cases of heterosis for induced biomass losses, we found that relative tolerance to BPH was associated with the R lines. This may be attributed to the smaller size of the B lines. Tolerance is strongly affected by plant size, and dwarf and semi-dwarf varieties have been implicated in the normally low tolerance of modern rice varieties to BPH [21]. 

Compared to BPH-infested plants where nitrogen improved yields in only one case, for WBPH, nitrogen increased yields in three cases. Furthermore, nitrogen increased the biomass accumulation of infested plants in all eight cases, compared to only three cases with BPH (Tables S8 and S12). Across hybrids, added nitrogen tended to increase absolute plant weight losses but decreased proportional losses (Table 2), indicating that the plants could grow larger under added nitrogen despite the higher biomass of WBPH on the same plants. This supports the hypothesis that BPH more effectively preempts nutrients than the host plant, whereas added nutrients continue to support plant growth under WBPH infestations [18]. High nitrogen plants also had higher survival (100%) compared to low nitrogen plants (83% in some cases) under WBPH infestation (Table S12), thereby supporting the hypothesis that nitrogen improves tolerance [46,61,62]; however, this was not observed to any greater extent among the hybrids, as indicated by the lack of heterosis related to biomass losses (Table 2). In the case of YSB, nitrogen improved yields in four cases and biomass accumulation in six cases, despite also improving the herbivore’s fitness and increasing absolute and proportional biomass losses in all cases (Table S16). Furthermore, as with WBPH, adding nitrogen increased plant survival when infested with stemborers (100% versus < 83% in some cases with high and low nitrogen, respectively: Table S16), but this was not more prominent on hybrids (Table S14). Rice compensation for stemborer damage is well documented [50,61,62] and has been attributed to increased tillering, greater production of reproductive tillers, larger grain and increased photosynthesis in damaged plants, as well as the transport of assimilates from damaged to healthy tillers [61,62,63,64,65]. Overall, our results with these two species indicate that the relative resistance (WBPH) and tolerance (WBPH and YSB) of hybrids was not necessarily associated with heterosis. Further detailed studies on hybrid tolerance are warranted.

### 4.5. Recommendations

We detected a number of cases of heterosis for resistance and tolerance (based on biomass losses) in our study. We also identified specific combinations of parents that resulted in heterobeltiosis for resistance or tolerance. Of specific note is one case of relatively good resistance to BPH despite an apparent absence of major resistance genes among the parental lines. In many cases of heterosis, improved resistance and tolerance were attributed to the R lines, indicating a continuing issue with susceptibility to herbivores among the male-sterile parents [10,19,20]. This has been linked to the A line cytoplasm in previous studies [20]; however, we suggest that the condition is likely associated with the nuclear genome and possibly a consequence of dwarfism as suggested by the relatively small size of B line plants. Despite these issues, we found no association between heterosis or heterobeltiosis and improved resistance traits of hybrids across accessions. To examine heterosis, we reported a large number of different herbivore and plant responses. These details were useful in indicating possible resistance sources; however, because heterosis had little bearing on the relative resistance or tolerance of hybrids, then such detailed studies are not practical or useful for directing breeding programs. It is also difficult to predict the outcome of crossings based on parental traits alone. 

In contrast to their use in breeding studies, detailed studies have been useful to test hypotheses concerning relative herbivore–rice interactions under varying nitrogen conditions and depending on plant type, particularly since IRRI allows access to parental lines. However, our study was affected by trade-offs between the number of plants (breeding groups, accessions and replicates) in the experiments and the optimal conditions for plant growth (i.e., pot size). Furthermore, the requirements to adequately assess relative tolerance levels [43] are difficult to achieve for such large experiments. We recommend that future studies would limit the time of plant exposure to herbivores and thereby avoid intraspecific crowding and that infestation rates would not result in extensive plant damage or a need to allow for plant recovery, as was carried out in our experiments, such that plants could be harvested at the same time that herbivore pressures (numbers and biomass) are recorded. Furthermore, we noted that varying numbers of nymphs after multiple generations of infestations can confound certain metrics associated with plant tolerance; therefore, development stages and population sizes should be kept similar across test plants.

Finally, our results indicate that the susceptibility of rice to BPH must be avoided to improve rice tolerance, and that high nitrogen conditions, despite improving herbivore fitness, also increase tolerance to herbivores such as WBPH, in agreement with observations for other rice herbivores [46,61,62]. Our results support evidence that in well-managed fields of moderately resistant rice with healthy numbers of natural enemies, high nitrogen conditions alone will not induce herbivore outbreaks.

## 5. Conclusions

Our results support the hypotheses that hybrid resistance to insect herbivores declines and that tolerance increases under high soil nitrogen conditions. For rice plants infested with BPH, increasing tolerance is conditional on at least moderate resistance as demonstrated with IR85471H. In the case of plants infested with WBPH, tolerance manifested as increasing yields and plant biomass, as well as improved plant survival under added nitrogen, and declining biomass losses per mg of insect. For plants infested with YSB, yields, plant biomass and plant survival increased under added nitrogen, but biomass losses per mg of insect also increased. Hybrids were not generally more resistant or tolerant than their associated parental lines in our experiments, with infestations confined to relatively young plants, although several cases of positive heterobeltiosis were detected. Furthermore, heterosis did not determine relative resistance or tolerance across hybrids. Our results do not support the general hypothesis that hybrids respond better than their parental lines to added nitrogen through increased tolerance; however, we suggest that root competition may have been more severe for the potted hybrids than for other plant types. Across herbivore species, there was no indication that heterosis for resistance and heterosis for tolerance necessarily co-occurred in the same plants, thereby suggesting that these traits occur independently. 

## Figures and Tables

**Table 1:** Summary of results for brown planthopper fitness and plant responses on hybrid lines (see Tables S7 and S8 for full details).

Accession	Added Nitrogen (Kg ha^−1^) ^1^	Total Number of BPH per Plant ^1^	Dry Weight of BPH per Plant (mg) ^1^	Plant Biomass Loss (Dry g) ^1^	Plant Biomass Loss (Proportion) ^1^	Plant Biomass Loss per mg of BPH (g mg^−1^) ^1^
IR82396H	0	509.17 ± 100.73 [ht B]	41.77 ± 7.32 ab [ht A]	2.58 ± 0.78 [ht A/B]	0.29 ± 0.08	0.07 ± 0.02
	150	609.00 ± 128.57	89.57 ± 13.81	7.88 ± 0.55	0.48 ± 0.03	0.11 ± 0.02
IR82391H	0	294.17 ± 49.28	34.51 ± 4.62 ab	5.32 ± 0.65	0.56 ± 0.06	0.16 ± 0.02
	150	867.17 ± 77.53	112.39 ± 6.03	8.58 ± 1.80	0.58 ± 0.11	0.08 ± 0.01
IR84714H	0	535.17 ± 101.06	52.62 ± 6.57 b [ht A]	5.55 ± 1.82	0.52 ± 0.15	0.11 ± 0.02
	150	928.00 ± 81.58	139.81 ± 19.96	10.69 ± 1.13	0.58 ± 0.05	0.08 ± 0.01
IR85471H	0	477.83 ± 108.79	50.08 ± 5.49 a	7.00 ± 0.78	0.62 ± 0.06	0.15 ± 0.00
	150	392.17 ± 88.12	45.62 ± 8.39	4.15 ± 1.59	0.24 ± 0.10	0.17 ± 0.01
IR81954H	0	720.00 ± 60.99 [hb^—^ A/B] †	47.28 ± 3.16 b [ht B] †	4.28 ± 1.22 [ht A/B] †	0.43 ± 0.09 [ht B] †	0.10 ± 0.01
	150	818.50 ± 123.40	127.47 ± 22.98	5.63 ± 2.17	0.32 ± 0.12	0.04 ± 0.01
IR80637H	0	502.67 ± 104.74	48.27 ± 4.54 ab	3.98 ± 1.23 [ht B] †	0.43 ± 0.12 [ht B] †	0.08 ± 0.01
	150	577.67 ± 152.17	107.59 ± 19.84	6.09 ± 0.89	0.52 ± 0.06	0.07 ± 0.01
IR82385H	0	523.00 ± 154.97 [ht A/B] †	39.59 ± 2.78 ab [ht A]	5.76 ± 2.17 [ht A/B] †	0.49 ± 0.17 [ht A/B] †	0.13 ± 0.00 [ht B]
	150	793.17 ± 166.86	105.62 ± 15.04	8.17 ± 0.81	0.50 ± 0.05	0.09 ± 0.01
IR82363H	0	312.67 ± 65.04 [ht B] †	57.52 ± 9.33 b	3.98 ± 0.59	0.49 ± 0.06 [ht A] †	0.07 ± 0.01
	150	890.83 ± 118.06	101.19 ± 6.36	7.16 ± 2.64	0.48 ± 0.15	0.07 ± 0.01
F-Accession (A) ^2^		1.741	3.041 **	1.325	1.225	1.276
F-Nitrogen (F) ^2^		14.054 ***	94.520 ***	3.273	3.997 *	13.770 ***
F-A × F ^2^		2.318 *	3.648 ***	1.518	1.280	0.854
F-Covariate				26.352 ***	5.284 *	12.083 ***
DF Error		80	80	79	79	79

^1^: Numbers are means ± standard errors (N = 6); lowercase letters indicate homogenous hybrid line groups based on Tukey’s LSD tests (*p* > 0.05); ht indicates heterosis based on a significantly different parameter value for the hybrid compared to one of its associated parental lines and based on A or B lines as indicated in square brackets; hb^—^ indicates heterobeltiosis for susceptibility based on a significantly higher parameter value for the hybrid compared to its associated parental lines (as indicated); † indicates resistance associated with the R line; for statistical results, see Tables S5 and S6. ^2^: Degrees of freedom: accession = 7, nitrogen = 1, interaction = 7 and error DF are indicated in the table; numbers are F-values, * = *p* ≤ 0.05, ** = *p* ≤ 0.01 and *** = *p* ≤ 0.005.

**Table 2:** Summary of results for whitebacked planthopper fitness and plant responses on hybrid lines (see Tables S11 and S12 for full details).

Accession	Added Nitrogen (Kg ha^−1^) ^1^	Total Number of WBPH per Plant ^1^	Dry Weight of WBPH per Plant (mg) ^1^	Plant Biomass Loss (Dry g) ^1^	Plant Biomass Loss (Proportion) ^1^	Plant Biomass Loss per mg of WBPH (g mg^−1^) ^1^
IR82396H	0	82.00 ± 19.35 [ht A/B] †	11.49 ± 5.25 ab [ht A/B] †	4.59 ± 0.46 ab	0.55 ± 0.04 b	0.71 ± 0.18
	150	33.83 ± 24.23	21.13 ± 2.96	3.13 ± 150.39	0.19 ± 0.08	0.15 ± 0.08
IR82391H	0	17.50 ± 6.95	3.71 ± 0.31 a [ht B]	3.19 ± 0.46 ab	0.34 ± 0.05 ab	0.89 ± 0.14
	150	47.00 ± 15.52	15.26 ± 3.45	3.79 ± 150.63	0.26 ± 0.12	0.42 ± 0.21
IR84714H	0	30.83 ± 14.80	8.04 ± 3.40 a	0.97 ± 0.58 ab	−0.01 ± 0.20 ab	0.26 ± 0.48
	150	92.67 ± 25.56	23.90 ± 6.12	6.91 ± 150.24	0.36 ± 0.05	0.35 ± 0.07
IR85471H	0	135.50 ± 73.95	8.85 ± 2.04 a	3.91 ± 0.67 b	0.34 ± 0.04 ab	0.51 ± 0.08
	150	54.50 ± 23.44	18.64 ± 7.53	6.26 ± 150.01	0.37 ± 0.05	0.67 ± 0.21
IR81954H	0	42.50 ± 17.18	8.84 ± 2.12 a [ht B]	2.37 ± 0.52 ab	0.18 ± 0.18 ab	0.24 ± 0.24
	150	51.50 ± 22.27	14.44 ± 3.60	2.77 ± 150.20	0.14 ± 0.11	0.22 ± 0.16
IR80637H	0	61.17 ± 28.24	18.48 ± 1.63 b [ht A/B]	2.94 ± 0.02 ab	0.33 ± 0.12 ab	0.16 ± 0.05
	150	99.17 ± 13.58	34.30 ± 1.65	4.52 ± 150.37	0.39 ± 0.12	0.14 ± 0.04
IR82385H	0	49.17 ± 13.72	11.15 ± 1.64 a [ht A]	4.36 ± 0.97 ab	0.44 ± 0.10 ab	0.38 ± 0.08
	150	23.17 ± 10.23	7.35 ± 1.69	2.65 ± 150.52	0.01 ± 0.21	−0.01 ± 1.36
IR82363H	0	48.67 ± 27.89	25.66 ± 10.21 ab	1.57 ± 0.95 a	0.14 ± 0.11 a	0.07 ± 0.10
	150	59.50 ± 5.73	22.99 ± 6.96	2.49 ± 150.82	0.12 ± 0.12	0.20 ± 0.11
F-Accession (A) ^2^		1.197	4.259 ***	2.659 *	2.628 *	1.359
F-Nitrogen (F) ^2^		0.247	17.544 ***	36.309 ***	20.938 ***	8.988 ***
F-A × F ^2^		4.167 ***	2.049	1.625	1.666	1.073
F-Covariate ^2^				46.711 ***	57.837 ***	24.305 ***
DF Error		80	80	79	79	79

^1^: Numbers are means ± standard errors (N = 6); lowercase letters indicate homogenous hybrid line groups based on Tukey’s LSD tests (*p* > 0.05); ht indicates heterosis based on a significantly different parameter value compared to parental lines, based on A or B lines as indicated in square brackets; † indicates that resistance is associated with the R line; for statistical results, see Tables S9 and S10. ^2^: Degrees of freedom: accession = 7, nitrogen = 1, interaction = 7, covariate = 1 and error DF are indicated in the table; numbers are F-values, * = *p* ≤ 0.05 and *** = *p* ≤ 0.005.

**Table 3:** Summary of results for yellow stemborer effects and plant responses on hybrid lines (see Tables S15 and S16 for full details).

Accession	Added Nitrogen (Kg ha^−1^) ^1^	Total Number of Emerged Adults ^1^	Biomass of Emerged Adults (Dry mg) ^1^	Plant Biomass Loss (Dry g) ^1^	Plant Biomass Loss (Proportion) ^1^	Plant Biomass Loss per mg of YSB (g mg^−1^) ^1^
IR82396H	0.00	2.00 ± 0.41	14.58 ± 3.20	2.93 ± 1.06 ab	0.31 ± 0.08	0.24 ± 0.09
	150.00	4.17 ± 0.48	33.02 ± 5.74	8.38 ± 0.72	0.51 ± 0.04	0.32 ± 0.08
IR82391H	0.00	3.33 ± 0.80	20.57 ± 5.02	3.66 ± 0.81 ab	0.36 ± 0.06	0.37 ± 0.21
	150.00	2.83 ± 0.70	17.93 ± 3.42	6.64 ± 1.83	0.50 ± 0.11	0.37 ± 0.10
IR84714H	0.00	2.80 ± 0.58	17.24 ± 4.14 [hb− B; ht A] †	3.58 ± 1.43 ab [ht B] ^3^	0.31 ± 0.09 [hb− B/ht B] ^3^	0.25 ± 0.12
	150.00	3.00 ± 0.77	20.33 ± 4.58	7.42 ± 2.44	0.39 ± 0.12	0.45 ± 0.22
IR85471H	0.00	2.00 ± 0.41	13.75 ± 4.20 [hb− B; ht A] †	7.13 ± 1.89 b	0.58 ± 0.09	0.70 ± 0.34
	150.00	2.17 ± 0.31	19.42 ± 2.27	8.41 ± 1.36	0.50 ± 0.07	0.50 ± 0.12
IR81954H	0.00	2.40 ± 0.40	14.16 ± 1.52	4.92 ± 1.40 a	0.48 ± 0.08	0.39 ± 0.12
	150.00	1.40 ± 0.24	16.80 ± 5.21	3.99 ± 2.78	0.16 ± 0.17	0.49 ± 0.24
IR80637H	0.00	1.80 ± 0.58	13.08 ± 4.15	4.48 ± 0.47 ab	0.51 ± 0.07	0.40 ± 0.19
	150.00	1.50 ± 0.34	13.27 ± 3.23	6.29 ± 0.78	0.53 ± 0.05	0.89 ± 0.39
IR82385H	0.00	2.00 ± 0.63	9.75 ± 2.97	3.91 ± 1.37 ab	0.39 ± 0.10	0.53 ± 0.13
	150.00	2.67 ± 0.33	16.88 ± 2.91	9.94 ± 3.42	0.36 ± 0.08	0.87 ± 0.48
IR82363H	0.00	2.00 ± 0.52 [ht A/B]	12.65 ± 3.74 [ht A/B]	3.91 ± 0.78 ab	0.45 ± 0.07	0.33 ± 0.10
	150.00	2.00 ± 0.32	17.50 ± 3.85	6.22 ± 1.95	0.36 ± 0.09	0.71 ± 0.42
F-Accession (A) ^2^		1.979	1.275	2.477 *	1.488	1.038
F-Nitrogen (F) ^2^		0.346	5.411 *	38.792 ***	26.163 ***	6.309 *
F-A × F ^2^		1.380	0.758	2.054	1.614	0.674
F-Covariate ^2^				28.720 ***	64.451 ***	5.190 *
DF Error		64	64	64	64	64

^1^: Numbers are means ± standard errors (N = 2–6; see Table S13); lowercase letters indicate homogenous groups based on Tukey’s LSD tests (*p* > 0.05); ht indicates heterosis based on comparisons with A or B lines as indicated; hb^—^ indicates heterobeltiosis for susceptibility in comparison with B lines; † indicates that resistance is associated with the R line; for statistical results, see Tables S13 and S14. ^2^: Degrees of freedom: accession = 7, nitrogen = 1, interaction = 7, covariate (plant biomass equivalent) = 1 and error DF are indicated in the table; numbers are F-values, * = *p* ≤ 0.05 and *** = *p* ≤ 0.005. ^3^: Heterobeltiosis for susceptibility under high nitrogen conditions but heterosis under low nitrogen conditions.

## Data Availability

The data presented in this study are available from the corresponding author upon reasonable request.

## Supplementary Materials

The following supporting information can be downloaded at: https://www.mdpi.com/article/10.3390/insects15060416/s1. Table S1: Accumulated information for the eight hybrid lines used in the experiments; Table S2: Biomass of grain, shoots, and roots of eight hybrid lines and their associated parental lines; Table S3: Changes in plant biomass (g dry weight) due to the addition of fertilizer for eight hybrids and their associated parental lines; Table S4: Results of three-way GLMs for each hybrid breeding group; Table S5: Fitness parameters of BPH on eight hybrid lines and their associated parental lines; Table S6: Plant biomass and estimated weight losses for eight hybrid lines and their associated parental lines after infestation with BPH; Table S7: Summary of results for BPH fitness on hybrid lines; Table S8: Summary of results for BPH effects on hybrid lines; Table S9: Fitness parameters of WBPH on eight hybrid lines and their associated parental lines; Table S10: Plant biomass and estimated weight losses for eight hybrid lines and their associated parental lines after infestation with WBPH; Table S11: Summary of results for WBPH fitness on hybrid lines; Table S12: Summary of results for WBPH effects on hybrid lines; Table S13: Fitness parameters of YSB on eight hybrid lines and their associated parental lines; Table S14: Plant biomass and estimated weight losses for eight hybrid lines and their associated parental lines after infestation with YSB; Table S15: Summary of results for YSB fitness on hybrid lines; Table S16: Summary of results for YSB effects on hybrid lines.
