# Supplementary material for: Heterosis for Interactions between Insect Herbivores and 3-Line Hybrid Rice under Low and High Soil Nitrogen Conditions
Source: Insects. 2024 Jun 4;15(6):416. doi: 10.3390/insects15060416 (PMC11204250; doi:10.3390/insects15060416)
Supplement: Supplementary file 1 [file insects-15-00416-s001.zip › insects-3025663-supplementary.pdf]

Supplementary Information

Table S1. Information from field plots for the eight hybrid lines used in the experiments as reported by Horgan et al (2024)[1]

Hybrid	Plant development[2]	Plot Yield (as Tonnes ha^{-1})[2,3]	Field herbivores[2,4]
IR82396H	Ht[B] for plant biomass	Ht[B] 6.43±0.12	Ht [B] for virus vectors and leaffolders
IR82391H	Ht[B] for SPAD values (chlorophyll)	Ht[B] 5.73±0.30	Ht [B] for WH, YSB and leaffolders; Resistant/tolerant to leaffolder and stemborer damage
IR84714H	Ht[B] for SPAD values and 1000-grain weight	Hb+ 7.02±0.10	Ht [B] for WH; Resistant/tolerant to stemborer damage
IR85471H		Hb± 6.24±0.18	Ht [B] for WH and leaffolders; Resistant/tolerant to stemborer damage (WH)
IR81954H	Ht[B] for 1000-grain weight	Hb± 5.68±0.20	Ht [B] for virus vectors; Hb- for green hairy caterpillar
IR80637H	Hb+ for grain number and grain weight	5.20±0.33	
IR82385H	Hb+ for plant biomass	Ht[B] 6.38±0.10	Hb- for WH and YSB
IR82363H	Hb+ for grain weight	6.30±0.22	Ht [B] for WH and YSB; Hb- for green hairy caterpillar; Resistant/tolerant to stemborer damage

1: Horgan, F.G.; Bernal, C.C.; Ramal, A.F.; Almazan, M.L.P.; Mundaca, E.A.; Crisol-Martínez, E. Heterosis for resistance to insect herbivores in a 3-line hybrid rice system. *Insects* **2024**, *15*, 164. (reference number 11 in the main text)
2: Ht [B] = Heterosis [compared to respective B-lines]; Hb- = heterobeltiosis for susceptibility, Hb+ = heterobeltiosis for improved plant trait
3: yields are presented as means + standard errors
4: WH = whitehead, for further information on herbivores see original paper

Table S2. Biomass of grain, shoots and roots of eight hybrid lines and their associated parental lines in a greenhouse experiment with low and high soil nitrogen; total plant biomass is also presented

Accession	Biomass of Grain (Dry g)[1]		Biomass of Shoots (Dry g)[1]		Biomass of Roots (Dry g)[1]		Total Plant Biomass (Dry g)[1,2]	
	No Added Nitrogen	Added Nitrogen (150 Kg ha^{-1})[1]	No Added Nitrogen	Added Nitrogen (150 Kg ha^{-1})[1]	No Added Nitrogen	Added Nitrogen (150 Kg ha^{-1})[1]	No Added Nitrogen	Added Nitrogen (150 Kg ha^{-1})[1]
IR 80156 A	0.00±0.00	0.00±0.00a	7.24±0.84	12.68±1.25c	0.53±0.06	0.84±0.07b	7.78±0.90	13.51±1.32b
IR 80156 B	1.70±0.20	2.96±0.40c	3.37±0.35	4.64±0.59a	0.31±0.05	0.35±0.07a	5.38±0.43	7.94±1.05a
IR 82396 H	1.23±0.16	1.95±0.24b	4.60±0.41	9.47±0.64b	0.51±0.05	0.89±0.15b	6.33±0.46	12.31±0.48b
IR46 R	1.61±0.13	2.17±0.37bc	5.21±0.25	9.48±0.45b	0.52±0.10	0.85±0.05b	7.35±0.32	12.51±0.57b
F-Accession[3]	119.684***		31.047***		11.755***		10.230***	
F-Nitrogen[3]	17.878***		72.559***		21.043***		283.192***	
IR 79156 A	0.00±0.00	0.00±0.00a	8.16±0.98	9.36±1.39b	0.50±0.09	0.51±0.10	8.66±1.06	9.86±1.48
IR 79156 B	1.64±0.23	3.45±0.50c	4.09±0.37	7.56±1.06a	0.37±0.06	0.70±0.10	6.09±0.61	11.71±1.61
IR 82391 H	1.08±0.12	1.90±0.56b	6.44±0.39	10.36±1.05b	0.49±0.06	0.82±0.11	8.00±0.49	13.08±0.96
IR60819-34-2 R	1.79±0.20	2.72±0.35c	4.91±0.57	7.95±1.22ab	0.51±0.12	0.70±0.12	7.21±0.83	11.36±1.47
F-Accession[3]	54.346***		4.534**		0.822ns		0.656ns	
F-Nitrogen[3]	11.148***		16.129***		8.258**		20.544***	
IR 80559 A	0.00±0.00	0.00±0.00a	5.91±0.63	9.35±1.33	0.45±0.03	0.59±0.12	6.36±0.65	9.93±1.42a
IR 80559 B	0.00±0.00	0.00±0.00a	5.04±0.73	10.68±1.19	0.36±0.07	0.59±0.13	5.39±0.79	11.27±1.30a
IR 84714 H	1.22±0.28	2.53±0.44b	5.91±0.42	11.42±0.66	0.45±0.06	0.75±0.13	7.58±0.70	14.70±0.86b
IR60819-34-2 R	1.97±0.29	3.04±0.40b	6.40±0.44	8.78±0.27	0.52±0.07	0.60±0.08	8.89±0.63	12.42±0.40ab
F-Accession[3]	104.498***		0.663ns		0.770ns		4.950**	
F-Nitrogen[3]	9.317***		43.067***		6.858**		52.049***	
IR 80564 A	0.00±0.00	0.00±0.00a	7.90±0.87	11.75±0.50b	0.46±0.06	0.66±0.05b	8.36±0.92	12.41±0.55b
IR 80564 B	1.60±0.14	2.69±0.51b	3.38±0.49	6.88±0.74a	0.34±0.10	0.37±0.05a	5.33±0.70	9.95±0.73a
IR 85471 H	1.38±0.33	2.55±0.55b	6.60±0.76	10.04±0.79b	0.47±0.04	0.75±0.09b	8.45±0.62	13.34±0.37b
IR60819-34-2 R	1.97±0.29	3.04±0.40b	6.40±0.44	8.78±0.27b	0.52±0.07	0.60±0.08b	8.89±0.63	12.42±0.40b
F-Accession[3]	45.454***		15.718***		5.290***		9.290***	
F-Nitrogen[3]	8.477**		40.071***		8.359**		73.628***	
IR 70369 A	0.00±0.00	0.00±0.00a	8.97±0.73	12.84±1.30b	0.56±0.07	1.12±0.11b	9.53±0.80	13.96±1.39b
IR 70369 B	1.72±0.15	2.29±0.54b	4.75±0.27	7.19±1.30a	0.53±0.06	0.53±0.13a	7.00±0.43	10.01±1.78a
IR 81954 H	1.75±0.38	2.50±0.50b	4.46±0.59	8.34±1.40a	0.43±0.08	0.50±0.10a	6.64±0.80	11.34±1.55ab
IR72889-46-3-2-1 R	1.78±0.19	3.21±0.70b	3.94±0.37	6.80±0.71a	0.51±0.12	0.49±0.13a	6.23±0.60	10.51±1.46ab
F-Accession[3]	28.117***		11.523***		4.683**		3.169*	

F-Nitrogen[3]	2.452ns		19.876***		2.636ns		21.267***	
IR 73328 A	0.03±0.02	0.00±0.00a	8.55±0.71	11.50±0.83a	0.52±0.06	0.55±0.10a	9.11±0.75	12.05±0.92
IR 73328 B	1.97±0.16	2.66±0.36b	5.67±0.35	8.59±1.03b	0.56±0.03	0.58±0.09b	8.21±0.48	11.84±1.06
IR 80637 H	1.91±0.20	3.82±0.26b	5.33±0.35	7.02±0.60b	0.41±0.03	0.51±0.06b	7.65±0.44	11.35±0.57
IR73013-95-1-3-2 R	2.41±0.20	3.53±0.34b	5.74±0.18	8.40±0.37b	0.63±0.06	0.71±0.03b	8.77±0.34	12.64±0.55
F-Accession[3]	28.117***		13.803***		4.683**		1.222ns	
F-Nitrogen[3]	2.452ns		34.972***		2.636ns		49.776***	
IR 79125 A	0.00±0.00	0.00±0.00a	10.33±0.68	13.48±0.68b	0.72±0.08	0.77±0.12b	11.05±0.76	14.24±0.77c
IR 79125 B	1.62±0.15	2.66±0.51d	5.42±0.27	5.56±1.05a	0.53±0.07	0.47±0.09a	7.58±0.33	8.70±1.61a
IR 82385 H	0.78±0.14	1.24±0.30b	7.35±0.75	12.32±0.69b	0.63±0.07	0.75±0.06a	8.75±0.88	14.31±0.86bc
IR73717-46-1-3-3 R	1.40±0.04	1.66±0.19c	5.86±0.65	7.47±1.53a	0.63±0.09	0.82±0.19a	7.89±0.73	9.95±1.78ab
F-Accession[3]	51.248***		16.394***		4.683**		7.872***	
F-Nitrogen[3]	4.128*		5.871*		2.636ns		15.500***	
IR 68897 A	0.00±0.00	0.00±0.00a	7.46±0.81	12.50±1.46c	0.45±0.09	0.68±0.10b	7.91±0.90	13.17±1.55c
IR 68897 B	1.89±0.26	3.47±0.50b	3.90±0.35	4.60±0.43a	0.39±0.05	0.38±0.09a	6.18±0.55	8.45±0.91a
IR 82363 H	1.48±0.26	2.45±0.42b	4.33±0.46	7.95±0.83b	0.38±0.03	0.74±0.16a	6.19±0.55	11.13±1.07ab
SRT 3 R	2.11±0.17	2.79±0.40b	5.58±0.27	7.15±0.44b	0.49±0.06	0.54±0.09a	8.19±0.30	10.48±0.74ab
F-Accession[3]	83.365***		18.656***		4.683**		3.837*	
F-Nitrogen[3]	12.681***		24.683***		2.636ns		28.936***	

1: Numbers are means ± standard errors (N = 6); lowercase letters indicated homogenous parameter groups based on Tukey tests ($P > 0.05$).

2: Total plant biomass is presented as the sum of grain, shoot and root biomass for each accession

3: Degrees of freedom: accession = 3, nitrogen = 1, interactions (not shown) = 3, error = 40; * = $P < 0.05$, ** = $P < 0.01$, *** = $P < 0.001$; interactions were not significant.

Table S3. Changes in plant biomass (g dry weight) due to the addition of fertilizer for eight hybrids and their associated parental lines (see also Table S3)

Accession	Δ Grain (Dry g)[1]	Δ Shoot (Dry g)[1]	Δ Root (Dry g)[1]	Δ Plant (Dry g)[1]
IR80156A	0.00±0.00[a]	5.43±0.84[b]	0.30±0.03	5.74±0.86
IR 80156B	1.25±0.41[c]	1.27±0.83[a]	0.04±0.10	2.56±1.27
IR82396H	0.72±0.20[bc]	4.87±0.50[ab]	0.39±0.16	5.98±0.48
IR46R	0.56±0.26[b]	4.26±0.64[ab]	0.33±0.09	5.16±0.61
F-plant type[1]	18.986***	3.871*	2.160	1.989
IR79156A	0.00±0.00[a]	1.20±2.17	0.00±0.18	1.21±2.34
IR79156B	1.81±0.44[b]	3.48±1.07	0.33±0.12	5.62±1.57
IR82391H	0.82±0.60[ab]	3.92±1.04	0.33±0.09	5.07±0.87
IR60819-34-2R	0.93±0.33[ab]	3.04±1.30	0.19±0.18	4.15±1.52
F-plant type[1]	4.559**	0.828	0.503	1.804
IR80559A	0.00±0.00[a]	3.43±1.34	0.14±0.12	3.57±1.43[a]
IR80559B	0.00±0.00[a]	5.64±1.69	0.23±0.15	5.87±1.83[ab]
IR84714H	1.31±0.24[b]	5.51±0.60	0.30±0.15	7.13±0.53[b]
IR60819-34-2R	1.06±0.40[b]	2.38±0.62	0.08±0.10	3.53±0.97[a]
F-plant type[1]	13.021***	2.765	0.418	3.309*
IR80564A	0.00±0.00[a]	3.85±0.74	0.20±0.05	4.05±0.78
IR80564B	1.09±0.50[b]	3.50±1.13	0.03±0.13	4.62±1.22
IR85471H	1.17±0.56[b]	3.44±1.06	0.29±0.12	4.89±0.67
IR60819-34-2R	1.06±0.40[b]	2.38±0.62	0.08±0.10	3.53±0.97
F-plant type[1]	8.018***	0.363	0.357	0.149
IR70369A	0.00±0.00	3.88±1.36	0.55±0.13	4.43±1.47
IR70369B	0.57±0.48	2.44±1.17	0.00±0.12	3.01±1.55
IR81954H	0.76±0.78	3.88±1.52	0.07±0.14	4.70±1.74
IR72889-46-3-2-1R	1.43±0.88	2.86±0.98	-0.02±0.22	4.27±1.98
F-plant type[1]	0.904	0.327	2.617	0.215
IR73328A	-0.03±0.02[a]	2.95±0.72	0.02±0.09	2.94±0.78
IR73328B	0.69±0.33[b]	2.92±1.11	0.02±0.10	3.62±1.17
IR80637H	1.91±0.18[c]	1.69±0.67	0.10±0.07	3.70±0.60
IR73013-95-1-3-2R	1.13±0.39[b]	2.66±0.32	0.08±0.07	3.87±0.53
F-plant type[1]	22.699***	0.717	0.245	0.764
IR82385H	0.46±0.37[ab]	4.97±1.41	0.12±0.11	5.56±1.66
IR79125A	0.00±0.00[a]	3.15±0.55	0.05±0.10	3.20±0.62
IR79125B	1.04±0.47[b]	0.14±0.94	-0.06±0.09	1.12±1.42
IR82385H	0.46±0.37[ab]	4.97±1.41	0.12±0.11	5.56±1.66
IR73717-46-1-3-3R	0.26±0.19[ab]	1.62±1.76	0.19±0.21	2.07±2.05
F-plant type[1]	4.096*	2.769	0.561	1.240
IR68897A	0.00±0.00[a]	5.03±1.93	0.23±0.13	5.27±2.05
IR68897B	1.57±0.39[c]	0.70±0.66	-0.01±0.08	2.26±0.95
IR82363H	0.97±0.34[bc]	3.61±1.10	0.36±0.17	4.94±1.15
SRT 3R	0.68±0.28[b]	1.56±0.48	0.05±0.14	2.29±0.84
F-plant type[1]	15.940***	1.488	1.507	1.293

1: Δ = fertilizer treated – untreated; numbers are means ± standard errors (N = 6), lowercase numbers indicate homogenous accession groups (Tukey $P > 0.05$)

2: Degrees of freedom: accession = 3, error = 15; * = $P < 0.05$, ** = $P < 0.01$, *** = $P < 0.001$

Table S4. Results of three-way GLM for the effects of plant type and nitrogenous fertilizer on biomass of BPH, WBPH and YSB (see Figure 1)

Factor	Degrees of Freedom	Hybrid Breeding Group[1]							
		IR 82396 H	IR 82391 H	IR 84714 H	IR 85471 H	IR 81954 H	IR 80637 H	IR 82385 H	IR 82363 H
F-Insect (I)	2	14.098	72.536***	12.352***	7.496***	47.670***	23.433***	58.439***	32.316***
F-Plant type (P)	3	2.785*	1.762	0.838	1.249	0.475	2.888*	1.138	0.71
F-Nitrogen (N)	1	65.690***	15.426***	0.007	1.726	0.011	0.071	6.621**	3.497
F-Block	5	2.276	1.886	0.965	1.295	3.423**	0.314	2.670*	0.741
F-I×P	6	0.581	3.246**	0.819	1.387	0.535	2.168	2.154	0.707
F-I×N	2	2.925	1.813	0.69	2.173	0.093	0.22	4.848**	3.253*
F-P×F	3	0.802	1.71	0.711	1.689	0.857	0.858	0.77	0.833
F-I×P×F	6	0.797	3.343***	0.584	1.481	0.74	0.756	1.131	0.831
Error	115								

1: * = P < 0.05, ** = P < 0.01, *** = P < 0.001 (N = 6)

Table S5. Fitness parameters of BPH on eight hybrid lines and their associated parental lines

Accession	Added Nitrogen (Kg ha-1)[1]	Development Stages (Proportion)[1]						Proportion of Adults that were Female[1]	Proportion of Brachypterous Adults[1]		Total number of BPH per Plant/Cage[1]	Dry Weight of BPH per Plant/Cage (mg)[1]
		1st Instar	2nd Instar	3rd Instar	4th Instar	5th Instar	Adults		Females	Males		
IR 80156 A	0	0.39±0.04b	0.15±0.02ab	0.15±0.02	0.12±0.02ab	0.11±0.02	0.07±0.04	0.18±0.07	1.00±0.00	0.93±0.07	332.00±40.53ab	47.22±5.40
	150	0.23±0.05	0.17±0.02	0.23±0.04	0.18±0.02	0.16±0.02	0.03±0.01	0.49±0.12	1.00±0.00	0.95±0.03	797.33±111.58	67.36±6.80
IR 80156 B	0	0.36±0.08ab	0.07±0.01a	0.28±0.04	0.16±0.02ab	0.10±0.02	0.02±0.01	0.23±0.16	1.00±0.00	1.00±0.00	182.83±18.82a	34.12±4.50
	150	0.23±0.03	0.18±0.03	0.24±0.03	0.18±0.01	0.12±0.03	0.04±0.01	0.11±0.05	1.00±0.00	0.80±0.06	767.17±82.87	119.02±10.55
IR 82396 H	0	0.25±0.07ab	0.21±0.06b	0.16±0.04	0.15±0.06a	0.11±0.07	0.12±0.08	0.41±0.16	0.97±0.03	0.81±0.15	509.17±100.73ab	41.77±7.32
	150	0.26±0.04	0.23±0.01	0.20±0.02	0.13±0.01	0.16±0.01	0.03±0.01	0.21±0.08	0.58±0.21	0.94±0.03	609.00±128.57	89.57±13.81
IR46 R	0	0.21±0.05a	0.21±0.02ab	0.24±0.02	0.19±0.03b	0.11±0.03	0.04±0.03	0.20±0.08	1.00±0.00	0.90±0.07	707.67±146.35b	57.45±3.57
	150	0.13±0.04	0.18±0.04	0.28±0.05	0.24±0.02	0.15±0.05	0.04±0.01	0.38±0.08	0.81±0.16	0.80±0.09	649.50±98.29	113.35±13.18
F-Accession (A)[2]		3.011*	3.035*	2.704	3.239*	0.285	0.583	0.452	2.379	0.380	2.952*	3.175*
F-Nitrogen (F)[2]		7.026**	1.465	1.313	1.933	2.714	1.436	0.003	3.080	3.411	23.743***	57.991***
F-A×F[2]		1.080	1.935	1.020	0.889	0.049	0.895	2.235	1.193	1.280	6.286***	4.219**
DF Error		40	40	40	40	40	40	38	23	23	40	40
IR 79156 A	0	0.23±0.02	0.25±0.04	0.29±0.01b	0.13±0.03	0.08±0.02a	0.02±0.01	0.32±0.10	1.00±0.00	0.94±0.04	513.33±75.55	44.04±2.45
	150	0.20±0.05	0.22±0.03	0.29±0.03	0.18±0.04	0.10±0.03	0.01±0.00	0.05±0.03	1.00±0.00	1.00±0.00	788.50±36.02	74.93±4.07
IR 79156 B	0	0.26±0.07	0.19±0.04	0.16±0.01a	0.12±0.02	0.14±0.04ab	0.12±0.09	0.09±0.07	1.00±0.00	0.87±0.10	491.67±101.92	39.00±2.75
	150	0.22±0.04	0.19±0.02	0.18±0.05	0.15±0.04	0.19±0.03	0.07±0.02	0.66±0.07	0.98±0.02	0.78±0.07	535.50±66.68	84.04±6.34
IR 82391 H	0	0.29±0.08	0.24±0.04	0.17±0.02ab	0.13±0.03	0.15±0.07ab	0.02±0.01	0.14±0.06	1.00±0.00	0.82±0.16	294.17±49.28	34.51±4.62
	150	0.11±0.03	0.20±0.03	0.30±0.02	0.18±0.03	0.16±0.01	0.05±0.02	0.34±0.10	0.82±0.16	0.84±0.08	867.17±77.53	112.39±6.03
IR60819-34-2 R	0	0.13±0.03	0.22±0.05	0.14±0.05ab	0.18±0.03	0.19±0.02b	0.13±0.09	0.28±0.08	1.00±0.00	1.00±0.00	487.67±94.25	60.79±4.64
	150	0.17±0.03	0.12±0.01	0.27±0.03	0.14±0.02	0.21±0.02	0.08±0.04	0.40±0.13	1.00±0.00	0.95±0.04	383.83±77.60	64.80±17.33
F-Accession (A)[2]		1.192	1.478	4.572**	0.309	3.849*	1.689	1.780	0.429	0.724	2.624	0.145
F-Nitrogen (F)[2]		2.889	3.656	9.430***	0.759	1.299	0.255	6.037*	0.516	3.087	10.165***	53.987***
F-A×F[2]		1.889	0.831	2.245	1.053	0.081	3.130	5.653	0.429	1.527	5.153***	10.684***
DF Error		40	40	40	40	40	40	40	24	24	40	40
IR 80559 A	0	0.11±0.05	0.17±0.06	0.27±0.03	0.25±0.05	0.19±0.07	0.02±0.01	0.08±0.03ab	1.00±0.00b	0.99±0.01b	409.00±83.85	46.03±4.03ab
	150	0.20±0.05	0.16±0.02	0.24±0.02	0.18±0.04	0.17±0.03	0.05±0.02	0.34±0.14	0.97±0.03	0.99±0.01	734.00±158.92	75.79±12.18
IR 80559 B	0	0.23±0.05	0.25±0.02	0.31±0.02	0.14±0.03	0.08±0.01	0.00±0.00	a	a	a	755.67±32.37	36.59±4.88a
	150	0.22±0.03	0.25±0.01	0.22±0.02	0.13±0.01	0.17±0.03	0.00±0.00	0.09±0.04	0.33±0.33	0.69±0.19	709.00±163.24	79.70±18.24
IR 84714 H	0	0.28±0.07	0.21±0.02	0.19±0.02	0.15±0.03	0.13±0.03	0.05±0.02	0.30±0.09ab	1.00±0.00b	0.98±0.02b	535.17±101.06	52.62±6.57b

	150	0.18±0.04	0.20±0.03	0.23±0.03	0.18±0.02	0.10±0.02	0.11±0.08	0.25±0.09	0.99±0.01	0.93±0.03	928.00±81.58	139.81±19.96
IR60819-34-2 R	0	0.19±0.04	0.18±0.01	0.21±0.05	0.18±0.02	0.20±0.03	0.04±0.02	0.63±0.15b	0.90±0.06b	0.79±0.10b	432.17±177.14	49.40±8.97ab
	150	0.13±0.05	0.19±0.06	0.17±0.02	0.11±0.02	0.18±0.04	0.22±0.08	0.58±0.09	1.00±0.00	0.99±0.01	393.50±88.06	67.02±7.17
F-Accession (A)[2]		1.358	2.693	2.630	2.672	2.136	3.601*	4.938**	9.511***	4.582*	2.583	3.221*
F-Nitrogen (F)[2]		0.230	0.009	1.981	1.911	0.006	5.773*	0.096	0.035	0.984	1.451	20.127***
F-A×F[2]		1.426	0.037	1.756	1.293	1.140	1.772	1.198	0.036	1.480	1.563	0.930
DF Error		40	40	40	40	40	40	29	19	19	40	40
IR 80564 A	0	0.22±0.05b	0.17±0.01	0.24±0.00ab	0.21±0.03ab	0.16±0.03ab	0.00±0.00a	0.00±0.00a		b	316.67±37.16	24.24±3.67
	150	0.26±0.03	0.23±0.02	0.23±0.01	0.17±0.03	0.10±0.01	0.02±0.01	0.15±0.08	1.00±0.00	1.00±0.00	744.00±102.31	79.33±8.45
IR 80564 B	0	0.15±0.05b	0.21±0.04	0.25±0.03ab	0.22±0.04ab	0.15±0.02a	0.02±0.01a	0.07±0.05a	1.00±0.00	0.83±0.17a	352.83±41.24	38.96±6.04
	150	0.33±0.04	0.22±0.02	0.22±0.05	0.12±0.02	0.09±0.02	0.02±0.01	0.12±0.07	0.80±0.00	0.91±0.05	571.67±47.55	74.66±7.09
IR 85471 H	0	0.09±0.04a	0.22±0.04	0.28±0.04b	0.25±0.03b	0.14±0.02ab	0.03±0.01a	0.12±0.06a	0.67±0.33	0.98±0.02b	477.83±108.79	50.08±5.49
	150	0.13±0.02	0.16±0.01	0.31±0.03	0.23±0.02	0.13±0.01	0.04±0.02	0.26±0.09	1.00±0.00	0.99±0.01	392.17±88.12	45.62±8.39
IR60819-34-2 R	0	0.18±0.04ab	0.17±0.01	0.21±0.05a	0.19±0.02a	0.20±0.03b	0.05±0.02b	0.51±0.12b	0.96±0.04	0.81±0.09ab	392.50±145.99	49.40±8.97
	150	0.14±0.04	0.15±0.05	0.17±0.02	0.13±0.02	0.17±0.03	0.25±0.09	0.54±0.10	1.00±0.00	0.96±0.02	391.67±151.30	67.52±7.16
F-Accession (A)[2]		5.085***	1.642	3.316*	3.268*	3.365*	7.806***	7.074***	1.014	3.686*	1.875	0.938
F-Nitrogen (F)[2]		3.489	0.037	0.227	7.609**	5.081*	5.645*	0.619	0.223	0.276	2.484	20.496***
F-A×F[2]		2.678	1.614	0.453	0.832	0.571	4.374**	0.109	2.582	7.428***	1.962	6.077
DF Error		40	40	40	40	40	40	30	17	17	40	40
IR 70369 A	0	0.16±0.11	0.07±0.02	0.27±1.07	0.18±0.05	0.19±0.07	0.11±0.06	0.07±0.07		1.00±0.00	323.17±60.69ab	39.80±4.84ab
	150	0.23±0.07	0.16±0.04	0.19±0.04	0.10±0.02	0.15±0.05	0.18±0.15	0.16±0.08	1.00±0.00	0.81±0.16	617.50±141.43	102.19±20.61
IR 70369 B	0	0.14±0.04	0.18±0.04	0.29±0.01	0.19±0.03	0.18±0.03	0.03±0.01	0.36±0.09	1.00±0.00	0.93±0.04	657.00±42.02bc	55.42±2.58b
	150	0.12±0.03	0.16±0.04	0.29±0.01	0.19±0.03	0.15±0.04	0.09±0.07	0.16±0.10	1.00±0.00	1.00±0.00	660.00±125.47	122.45±4.24
IR 81954 H	0	0.19±0.04	0.22±0.03	0.33±0.06	0.15±0.02	0.10±0.03	0.00±0.00	0.33±0.33		0.96±0.04	720.00±60.99c	47.28±3.16ab
	150	0.10±0.04	0.11±0.02	0.26±0.04	0.23±0.03	0.20±0.05	0.10±0.04	0.43±0.14	1.00±0.00	0.80±0.20	818.50±123.40	127.47±22.98
IR72889-46-3-2-1 R	0	0.22±0.03	0.20±0.02	0.19±0.02	0.15±0.02	0.19±0.04	0.04±0.01	0.18±0.08	1.00±0.00	1.00±0.00	296.67±34.46a	38.53±6.15a
	150	0.11±0.04	0.20±0.07	0.18±0.05	0.17±0.03	0.17±0.04	0.16±0.08	0.27±0.09	0.97±0.03	0.79±0.16	355.17±92.97	85.98±31.07
F-Accession (A)[2]		0.620	1.974	2.488	1.290	0.156	0.831	2.225	0.368	0.720	9.693***	3.147*
F-Nitrogen (F)[2]		1.039	0.135	1.980	0.061	0.007	2.908	0.001	0.287	0.885	1.158	26.622***
F-A×F[2]		0.904	2.322	0.484	2.473	1.088	0.044	0.439	0.370	0.640	1.009	0.386
DF Error		40	40	40	40	40	40	36	22	22	40	40
IR 73328 A	0	0.14±0.05	0.18±0.05	0.27±0.03	0.18±0.02	0.16±0.06	0.06±0.03	0.29±0.05	0.76±0.19ab	0.96±0.04	406.00±26.31	52.23±6.38
	150	0.08±0.03	0.11±0.03	0.23±0.05	0.22±0.04	0.22±0.03	0.14±0.10	0.22±0.05	0.90±0.10	0.84±0.13	484.50±125.66	100.46±27.06
IR 73328 B	0	0.23±0.11	0.12±0.04	0.18±1.05	0.19±0.05	0.15±0.03	0.12±0.09	0.18±0.07	1.00±0.00b	0.99±0.01	351.83±54.23	47.19±4.89
	150	0.13±0.04	0.16±0.03	0.19±0.03	0.24±0.02	0.22±0.04	0.06±0.03	0.37±0.14	1.00±0.00	0.99±0.01	746.83±105.15	111.58±8.68

IR 80637 H	0	0.13±0.05	0.21±0.04	0.28±0.05	0.19±0.04	0.16±0.06	0.02±0.00	0.22±0.16	1.00±0.00b	0.93±0.05	502.67±104.74	48.27±4.54
	150	0.13±0.05	0.13±0.04	0.17±0.03	0.19±0.02	0.17±0.03	0.21±0.09	0.33±0.07	1.00±0.00	0.95±0.05	577.67±152.17	107.59±19.84
IR73013-95-1-3-2 R	0	0.17±0.08	0.13±0.05	0.26±0.02	0.23±0.06	0.19±0.07	0.02±0.01	0.10±0.04	0.83±0.17a	0.94±0.06	393.00±81.36	43.00±7.58
	150	0.12±0.07	0.16±0.06	0.31±0.05	0.21±0.05	0.19±0.04	0.02±0.00	0.03±0.03		0.97±0.03	776.50±126.20	96.55±13.69
F-Accession (A)[2]		0.495	0.189	2.444	0.256	0.122	1.056	1.537	5.760***	0.924	0.424	0.327
F-Nitrogen (F)[2]		1.376	0.522	0.634	0.285	0.918	1.498	0.156	3.279	1.096	4.281*	19.591***
F-A×F[2]		0.232	1.173	1.447	0.458	0.241	1.707	0.776	3.664	0.406	1.773	0.675
DF Error		40	40	40	40	40	40	37	22	22	40	40
IR 79125 A	0	0.44±0.13b	0.10±0.03	0.11±1.03	0.11±0.03a	0.21±0.13	0.02±0.01	0.17±0.09	1.00±0.00	0.98±0.02	372.33±82.69ab	32.54±6.49
	150	0.12±0.05	0.18±0.05	0.27±0.04	0.18±0.05	0.11±0.02	0.14±0.13	0.23±0.08	1.00±0.00	1.00±0.00	764.67±120.94	111.02±13.51
IR 79125 B	0	0.12±0.04a	0.17±0.03	0.23±0.04	0.18±0.03b	0.19±0.03	0.11±0.03	0.33±0.04	0.77±0.17	0.84±0.08	196.00±33.24ab	38.48±6.07
	150	0.08±0.03	0.12±0.03	0.31±0.04	0.27±0.02	0.17±0.04	0.05±0.03	0.39±0.14	1.00±0.00	0.95±0.05	355.50±41.24	41.74±6.65
IR 82385 H	0	0.09±0.03ab	0.12±0.02	0.31±0.05	0.21±0.02ab	0.19±0.04	0.07±0.01	0.20±0.08	1.00±0.00	1.00±0.00	523.00±154.97b	39.59±2.78
	150	0.15±0.05	0.17±0.03	0.20±0.02	0.18±0.02	0.21±0.05	0.09±0.05	0.25±0.09	1.00±0.00	0.60±0.13	793.17±166.86	105.62±15.04
IR73717-46-1-3-3 R	0	0.17±0.07ab	0.15±0.05	0.18±0.05	0.16±0.02a	0.23±0.07	0.11±0.06	0.29±0.13	1.00±0.00	0.80±0.20	281.50±50.41a	48.92±9.90
	150	0.13±0.05	0.13±0.05	0.22±0.08	0.13±0.04	0.16±0.04	0.22±0.14	0.23±0.11	0.93±0.07	1.00±0.00	243.83±65.97	47.26±14.10
F-Accession (A)[2]		3.647*	0.016	1.240	3.061*	0.062	0.960	1.166	0.813	0.535	4.212*	3.139*
F-Nitrogen (F)[2]		4.132*	0.206	1.497	1.773	0.978	1.018	0.350	0.180	0.237	4.102*	12.606***
F-A×F[2]		3.488*	1.362	2.749	2.141	0.408	0.663	0.284	1.330	2.748	1.663	6.124***
DF Error		40	40	40	40	40	40	38	24	24	40	40
IR 68897 A	0	0.17±0.04	0.23±0.07	0.20±0.03b	0.14±0.03	0.15±0.02	0.12±0.11	0.35±0.09	1.00±0.00	1.00±0.00	281.00±70.88a	42.31±7.56
	150	0.09±0.02	0.16±0.01	0.28±0.04	0.24±0.02	0.21±0.04	0.02±0.01	0.16±0.04	0.75±0.25	0.98±0.02	390.83±119.06	100.98±7.59
IR 68897 B	0	0.33±0.04	0.26±0.03	0.18±0.02a	0.16±0.03	0.06±0.01	0.01±0.00	0.10±0.04	1.00±0.00	0.89±0.07	997.00±31.47b	62.29±3.52
	150	0.14±0.04	0.12±0.03	0.19±0.05	0.18±0.03	0.24±0.05	0.12±0.09	0.26±0.08	1.00±0.00	0.93±0.07	492.67±143.51	101.57±19.89
IR 82363 H	0	0.13±0.04	0.17±0.05	0.26±0.05b	0.20±0.03	0.17±0.04	0.07±0.05	0.39±0.09	1.00±0.00	0.88±0.13	312.67±65.04ab	57.52±9.33
	150	0.18±0.04	0.21±0.04	0.25±0.01	0.17±0.02	0.08±0.03	0.10±0.08	0.51±0.17	1.00±0.00	0.72±0.20	890.83±118.06	101.19±6.36
SRT 3 R	0	0.16±0.08	0.16±0.04	0.10±0.04a	0.14±0.03	0.22±0.11	0.22±0.13	0.46±0.18	1.00±0.00	0.73±0.19	264.50±126.37a	37.64±9.16
	150	0.20±0.08	0.19±0.06	0.18±0.03	0.15±0.03	0.12±0.03	0.15±0.08	0.33±0.08	1.00±0.00	0.79±0.08	556.17±153.57	83.35±14.34
F-Accession (A)[2]		1.491	0.070	4.335**	0.813	0.454	0.998	2.103	0.941	1.803	4.335**	2.139
F-Nitrogen (F)[2]		1.220	0.901	2.520	1.370	0.072	0.028	0.001	0.906	0.351	3.087	33.610***
F-A×F[2]		2.272	1.681	0.901	1.365	3.534	0.700	1.272	0.941	0.840	5.762***	1.139
DF Error		40	40	40	40	40	40	35	23	23	40	40

1: Numbers are means ± standard errors (N = 6); lowercase letters indicate homogenous groups based on Tukey tests (P > 0.05)

2: Degrees of freedom: accession = 7, nitrogen = 1, interaction = 7, error DF are indicated in the table; numbers are F-values, * = P ≤ 0.05, ** = P ≤ 0.01, *** = P ≤ 0.001

Table S6. Plant biomass and estimated weight losses for eight hybrid lines and their associated parental lines after infestation with BPH (see Table 1)

Accession	Added Nitrogen (Kg ha-1)[1]	Plant Condition (SES 1-9)[1,2]	Grain Biomass (Dry g)[1]	Shoot Biomass (Dry g)[1]	Root Biomass (Dry g)[1]	Plant Biomass Loss (Dry g)[1,3]	Plant Biomass Loss (Proportion)[1,3]	Plant Biomass Loss per mg of BPH (g mg-BPH^{-1})[1,3]
IR 80156 A	0	6.67±1.09		3.53±0.49b	0.37±0.10	3.88±1.04a	0.45±0.11	0.09±0.03
	150	7.33±0.80		5.57±0.53b	0.75±0.14	7.17±1.36	0.51±0.05	0.11±0.02
IR 80156 B	0	8.00±0.68	0.48±0.12	2.01±0.23a	0.39±0.01	4.54±0.61ab	0.56±0.06	0.14±0.03
	150	6.67±1.20	1.13±0.60	4.22±0.49a	0.74±0.08	5.62±2.78	0.37±0.17	0.04±0.02
IR 82396 H	0	5.00±1.03	0.98±0.17	2.82±0.28ab	0.36±0.07	2.58±0.78ab	0.29±0.08	0.07±0.02
	150	5.67±0.42	1.05±0.16	4.70±0.50ab	0.56±0.08	7.88±0.55	0.48±0.03	0.11±0.02
IR46 R	0	6.17±1.22	0.98±0.27	2.41±0.23a	0.48±0.06	4.54±1.26b	0.43±0.12	0.08±0.02
	150	5.67±1.23	1.21±0.42	3.80±0.48a	0.64±0.07	8.83±2.69	0.48±0.13	0.08±0.01
F-Accession (A)[4]		1.739	0.970	4.784**	0.850	2.874*	1.165	3.236*
F-Nitrogen (F)[4]		0.031	0.445>	36.695***	21.830***	1.135	4.163*	21.329***
F-A×F[4]		0.477	0.123	0.469	0.782	0.439	0.752	4.111*
F-Covariate[4]						29.188***	8.513**	27.252***
DF Error		40	30	40	40	37	39	39
IR 79156 A	0	6.33±0.99		4.29±0.74b	0.67±0.13b	3.69±1.49	0.33±0.17	0.09±0.02
	150	7.00±1.03		3.33±0.77	0.66±0.17	5.87±2.24	0.49±0.15	0.09±0.02
IR 79156 B	0	7.00±1.03	1.02±0.23	2.07±0.32a	0.40±0.06a	3.54±1.71	0.32±0.22	0.10±0.02
	150	9.00±0.00	0.00±0.00	1.62±0.19	0.30±0.00	12.25±2.22	0.85±0.03	0.16±0.02
IR 82391 H	0	7.00±0.89	0.26±0.12	3.18±0.44b	0.48±0.07ab	5.32±0.65	0.56±0.06	0.16±0.02
	150	7.00±1.03	0.71±0.34	3.91±0.84	0.72±0.11	8.58±1.80	0.58±0.11	0.08±0.01
IR60819-34-2	0	7.00±1.26	0.83±0.32	2.28±0.30ab	0.77±0.08b	4.76±1.94	0.38±0.19	0.07±0.01
	150	7.00±1.03	1.71±0.64	3.30±0.65	0.64±0.11	6.43±1.89	0.45±0.15	0.16±0.01
F-Accession (A)[4]		0.698	2.829	5.340***	5.420***	0.987	0.917	0.372
F-Nitrogen (F)[4]		0.930	0.124	0.006	0.008	2.986	0.001	8.441**
F-A×F[4]		0.465	5.265*	1.449	1.439	0.363	1.805	4.601**
F-Covariate[4]						24.483***	18.160***	45.418***
DF Error		40	30	40	40	36	39	39
IR 80559 A	0	7.67±0.84		2.53±0.52ab	0.51±0.07	3.32±0.77	0.50±0.08	0.08±0.01
	150	7.83±0.98		3.00±1.21	0.93±0.29	6.00±1.81	0.58±0.11	0.10±0.02
IR 80559 B	0	8.00±1.00	0.00±0.00a	2.50±0.81a	0.95±0.27	1.94±1.56	0.19±0.28	0.07±0.02
	150	8.67±0.21	0.00±0.00	1.45±0.11	0.55±0.15	9.27±1.42	0.80±0.05	0.26±0.02
IR 84714 H	0	8.00±1.00	0.25±0.13a	2.78±0.58b	0.44±0.06	5.55±1.82	0.52±0.15	0.11±0.02

	150	7.67±0.67	0.50±0.17	5.36±0.86	1.19±0.20	10.69±1.13	0.58±0.05	0.08±0.01
IR60819-34-2 R	0	7.00±0.73	1.01±0.23b	2.02±0.30ab	0.41±0.07	6.90±0.91	0.58±0.06	0.17±0.01
	150	6.67±1.20	1.80±0.43	3.81±0.62	0.74±0.13	7.92±1.35	0.48±0.09	0.13±0.01
F-Accession (A)[4]		1.018	23.162***	4.022*	0.653	0.972	1.851	1.846
F-Nitrogen (F)[4]		0.005	2.484>	3.004	5.282*	0.371	0.327	2.190
F-A×F[4]		0.149	0.739	2.847*	3.973*	2.075	2.396	2.138
F-Covariate[4]						22.297***	*	7.507***
DF Error		40	30	40	40	38	38	39
IR 80564 A	0	7.33±1.09		4.07±0.56b	0.27±0.07	4.03±0.95	0.47±0.07	0.17±0.02
	150	6.67±0.80		5.77±0.43	0.67±0.18	5.97±0.94	0.47±0.06	0.08±0.02
IR 80564 B	0	5.83±1.28	0.59±0.17	1.92±0.17a	0.25±0.03	4.69±1.54	0.47±0.13	0.12±0.01
	150	7.33±0.61	0.86±0.28	3.36±0.26	0.37±0.03	7.59±1.14	0.57±0.05	0.10±0.03
IR 85471 H	0	7.33±0.61	0.41±0.17	2.64±0.44b	0.42±0.10	7.00±0.78	0.62±0.06	0.15±0.00
	150	6.00±1.13	2.56±0.46	5.92±0.49	0.69±0.10	4.15±1.59	0.24±0.10	0.17±0.01
IR60819-34-2 R	0	6.67±0.80	1.01±0.23	2.02±0.30a	0.41±0.07	7.46±1.03	0.57±0.06	0.18±0.01
	150	6.67±1.20	1.80±0.43	3.89±0.63	0.74±0.13	7.49±1.25	0.48±0.09	0.12±0.01
F-Accession (A)[4]		0.072	2.549	11.381***	3.061*	0.464	0.850	0.202
F-Nitrogen (F)[4]		0.033	12.852***>	45.968***	16.403***	3.446	4.977*	6.034*
F-A×F[4]		0.778	3.900*	1.236	0.528	3.582*	3.114*	0.357
F-Covariate[4]						18.177***	2.177	4.076*
DF Error		40	30	40	40	38	39	39
IR 70369 A	0	6.67±1.09		3.25±0.78ab	0.54±0.04	5.74±1.49ab	0.56±0.12ab	0.15±0.01
	150	8.00±1.00		3.40±1.01	1.11±0.31	9.45±1.98	0.66±0.10	0.12±0.02
IR 70369 B	0	8.33±0.67	0.37±0.12a	1.50±0.28a	0.54±0.06	6.52±0.75b	0.69±0.05b	0.12±0.01
	150	8.67±0.33	0.40±0.18	2.39±0.25	0.46±0.01	10.94±2.96	0.66±0.08	0.09±0.01
IR 81954 H	0	8.67±0.33	0.85±0.16b	2.53±0.29b	0.26±0.03	4.28±1.22a	0.43±0.09a	0.10±0.01
	150	5.33±1.09	1.68±0.50	5.09±0.78	0.74±0.11	5.63±2.17	0.32±0.12	0.04±0.01
IR72889-46-3-2-1 R	0	6.33±1.23	0.89±0.30ab	2.59±0.44ab	0.39±0.08	3.61±1.47ab	0.37±0.20a	0.08±0.02
	150	7.17±1.05	1.24±0.68	3.49±0.93	0.50±0.12	9.63±3.26	0.54±0.15	0.27±0.01
F-Accession (A)[4]		1.446	3.284*	3.103*	2.792	3.439*	3.123*	1.269
F-Nitrogen (F)[4]		0.105	0.569>	5.584*	7.538**	2.375	0.538	2.855
F-A×F[4]		2.720	0.385	0.964	2.260	0.888	0.505	1.504
F-Covariate[4]						24.269***	3.695	9.578***
DF Error		40	30	40	40	38	39	39
IR 73328 A	0	6.67±1.20ab		3.33±0.72b	0.36±0.06	5.44±1.38a	0.56±0.11ab	0.11±0.01

	150	5.67±1.12		5.43±1.04	0.67±0.19	5.94±1.34	0.49±0.10	0.36±0.00
IR 73328 B	0	8.33±0.67b	0.48±0.19	1.58±0.22a	0.40±0.04	6.61±0.86b	0.70±0.06b	0.15±0.02
	150	8.33±0.67	0.29±0.24	2.01±0.50	0.51±0.11	9.25±1.40	0.76±0.07	0.09±0.01
IR 80637 H	0	4.83±0.91a	1.26±0.33	2.40±0.41b	0.38±0.07	3.98±1.23a	0.43±0.12a	0.08±0.01
	150	6.83±0.83	1.21±0.41	3.56±0.43	0.75±0.13	6.09±0.89	0.52±0.06	0.07±0.01
IR73013-95-1-3-2 R	0	5.67±0.84ab	1.39±0.31	2.74±0.20b	0.47±0.05	4.77±0.89a	0.46±0.07a	0.13±0.01
	150	7.00±0.89	1.69±0.38	4.12±0.44	0.62±0.12	6.39±0.51	0.50±0.05	0.07±0.01
F-Accession (A)[4]		3.093*	8.140**	8.335***	0.367	3.771*	4.980***	0.884
F-Nitrogen (F)[4]		0.823	0.081	9.858***	9.008***	0.696	1.168	1.861
F-A×F[4]		1.092	0.277	0.433	0.676	0.598	0.411	0.826
F-Covariate[4]						23.771***	4.556*	4.745*
DF Error		40	30	40	40	39	39	39
IR 79125 A	0	7.00±0.52b		4.73±0.30b	0.54±0.09b	5.78±0.72b	0.51±0.04b	0.22±0.04ab
	150	8.00±0.68		4.21±1.04	0.73±0.17	9.31±1.16	0.66±0.07	0.08±0.01
IR 79125 B	0	5.67±0.84b	0.84±0.25	2.43±0.37a	0.49±0.10a	5.41±0.78b	0.51±0.08b	0.17±0.01b
	150	7.67±0.67	0.73±0.25	2.05±0.29	0.16±0.02	10.38±1.81	0.71±0.07	0.36±0.00
IR 82385 H	0	8.00±1.00b	0.49±0.25	2.31±0.70ab	0.79±0.07b	5.76±2.17b	0.49±0.17ab	0.13±0.00a
	150	6.33±0.67	1.01±0.26	4.90±0.90	0.67±0.12	8.17±0.81	0.50±0.05	0.09±0.01
IR73717-46-1-3-3 R	0	3.17±0.17a	1.45±0.11	3.43±0.29ab	0.58±0.06b	2.33±0.52a	0.21±0.04a	0.07±0.01a
	150	5.33±1.20	0.93±0.41	4.19±1.02	0.85±0.21	5.37±2.61	0.39±0.14	0.16±0.01
F-Accession (A)[4]		7.304***	1.339	4.768**	6.587***	7.755***	3.573*	3.059*
F-Nitrogen (F)[4]		2.549	0.054	1.054	0.202	0.086	1.169	0.405
F-A×F[4]		2.611	2.579	3.415*	3.302*	0.700	0.856	3.341*
F-Covariate[4]						21.298***	1.010	3.312
DF Error		40	30	40	40	38	39	39
IR 68897 A	0	5.67±1.23		4.26±0.31	0.38±0.08a	3.28±0.98	0.37±0.09	0.08±0.01
	150	8.50±0.22		2.39±0.05	0.15±0.01	10.64±1.71	0.79±0.03	0.11±0.00
IR 68897 B	0	6.00±0.86	0.89±0.12a	2.36±0.10	0.30±0.03ab	4.13±1.22	0.41±0.09	0.07±0.03
	150	4.33±0.99	1.70±0.55	4.00±0.45	0.56±0.06	4.91±2.86	0.29±0.14	0.05±0.01
IR 82363 H	0	6.00±0.86	0.59±0.20ab	2.60±0.26	0.34±0.05b	3.98±0.59	0.49±0.06	0.07±0.01
	150	7.00±1.26	0.88±0.38	4.76±1.36	0.75±0.24	7.16±2.64	0.48±0.15	0.07±0.01
SRT 3 R	0	4.33±0.42	1.69±0.32b	2.89±0.30	0.43±0.05ab	3.80±0.80	0.34±0.07	0.12±0.01
	150	6.50±1.15	1.87±0.48	3.61±0.55	0.44±0.08	5.98±2.69	0.36±0.14	0.07±0.02
F-Accession (A)[4]		1.841	4.691*	0.038	3.493*	2.317	4.615**	1.694
F-Nitrogen (F)[4]		2.645	0.968>	1.447	1.902	0.978	0.939	9.580***

F-A×F[4]	2.217	0.247	4.703**	4.853**	1.718	3.228*	0.888
F-Covariate[4]					18.891***	11.951***	15.533***
DF Error	40	30	40	40	38	39	39

1: Numbers are means ± standard errors (N = 6); lowercase letters indicate homogenous groups based on Tukey tests (P > 0.05)

2: SES = Standard Evaluation System

3: Biomass loss was estimated for whole plants using grain to shoot conversion calculated as Biomass of A-line – Biomass of B-line/Biomass of B-line grain (see text for further details)

4: Degrees of freedom: accession = 7, nitrogen = 1, interaction = 7, covariate (plant biomass equivalent) = 1, error DF are indicated in the table; numbers are F-values, * = P ≤ 0.05, ** = P ≤ 0.01, *** = P ≤ 0.005

Table S7. Summary of results for BPH fitness on hybrid lines (see Table S1 for full details)

Accession	Added Nitrogen (Kg ha^{-1})[1]	Development Stages (Proportion)[1]						Proportion of Adults that were Female[1]	Proportion of Brachypterous Adults[1]		Total number of BPH per Plant/Cage[1]	Dry Weight of BPH per Plant/Cage (mg)[1]
		1st Instar	2nd Instar	3rd Instar	4th Instar	5th Instar	Adults		Females	Males		
IR 82396 H	0	0.25±0.07	0.21±0.06 [A/B]	0.16±0.04	0.15±0.06a [A/B]	0.11±0.07	0.12±0.08	0.41±0.16	0.97±0.03	0.81±0.15	509.17±100.73 [B]	41.77±7.32ab [A]
	150	0.26±0.04	0.23±0.01	0.20±0.02	0.13±0.01	0.16±0.01	0.03±0.01	0.21±0.08	0.58±0.21	0.94±0.03	609.00±128.57	89.57±13.81
IR 82391 H	0	0.29±0.08	0.24±0.04	0.17±0.02	0.13±0.03ab	0.15±0.07 [A]	0.02±0.01	0.14±0.06	1.00±0.00	0.82±0.16	294.17±49.28	34.51±4.62ab
	150	0.11±0.03	0.20±0.03	0.30±0.02	0.18±0.03	0.16±0.01	0.05±0.02	0.34±0.10	0.82±0.16	0.84±0.08	867.17±77.53	112.39±6.03
IR 84714 H	0	0.28±0.07	0.21±0.02	0.19±0.02	0.15±0.03ab	0.13±0.03	0.05±0.02	0.30±0.09 [B]	1.00±0.00 [B]	0.98±0.02	535.17±101.06	52.62±6.57b [A]
	150	0.18±0.04	0.20±0.03	0.23±0.03	0.18±0.02	0.10±0.02	0.11±0.08	0.25±0.09	0.99±0.01	0.93±0.03	928.00±81.58	139.81±19.96
IR 85471 H	0	0.09±0.04	0.22±0.04	0.28±0.04	0.25±0.03b	0.14±0.02	0.03±0.01	0.12±0.06 [A/B]	0.67±0.33 {B]	0.98±0.02	477.83±108.79	50.08±5.49a
	150	0.13±0.02	0.16±0.01	0.31±0.03	0.23±0.02	0.13±0.01	0.04±0.02	0.26±0.09	1.00±0.00	0.99±0.01	392.17±88.12	45.62±8.39
IR 81954 H	0	0.19±0.04	0.22±0.03	0.33±0.06	0.15±0.02ab	0.10±0.03	0.00±0.00	0.33±0.33		0.96±0.04	720.00±60.99 [Hb A/Ht B]†	47.28±3.16b [B]†
	150	0.10±0.04	0.11±0.02	0.26±0.04	0.23±0.03	0.20±0.05	0.10±0.04	0.43±0.14	1.00±0.00	0.80±0.20	818.50±123.40	127.47±22.98
IR 80637 H	0	0.13±0.05	0.21±0.04	0.28±0.05	0.19±0.04ab	0.16±0.06	0.02±0.00	0.22±0.16†	1.00±0.00 [A]	0.93±0.05	502.67±104.74	48.27±4.54ab
	150	0.13±0.05	0.13±0.04	0.17±0.03	0.19±0.02	0.17±0.03	0.21±0.09	0.33±0.07	1.00±0.00	0.95±0.05	577.67±152.17	107.59±19.84
IR 82385 H	0	0.09±0.03	0.12±0.02	0.31±0.05	0.21±0.02ab	0.19±0.04	0.07±0.01	0.20±0.08	1.00±0.00	1.00±0.00	523.00±154.97 [A/B]†	39.59±2.78ab [A]
	150	0.15±0.05	0.17±0.03	0.20±0.02	0.18±0.02	0.21±0.05	0.09±0.05	0.25±0.09	1.00±0.00	0.60±0.13	793.17±166.86	105.62±15.04
IR 82363 H	0	0.13±0.04	0.17±0.05	0.26±0.05	0.20±0.03ab	0.17±0.04	0.07±0.05	0.39±0.09	1.00±0.00	0.88±0.13	312.67±65.04 [B]†	57.52±9.33b
	150	0.18±0.04	0.21±0.04	0.25±0.01	0.17±0.02	0.08±0.03	0.10±0.08	0.51±0.17	1.00±0.00	0.72±0.20	890.83±118.06	101.19±6.36
F-Accession (A)[2]		2.522*	1.049	2.195*	2.469*	0.885	0.686	1.060	1.781	1.899	1.741	3.041**
F-Nitrogen (F)[2]		1.426	1.824	0.221	0.179	0.116	3.504	0.292	0.615	8.269**	14.054***	94.520***
F-A×F[2]		1.705	1.434	2.670*	1.019	1.038	1.459	0.567	1.886	1.038	2.318*	3.648***
DF Error		80	80	80	80	80	80	80	42	42	80	80

1: Numbers are means ± standard errors (N = 6); lowercase letters indicate homogenous hybrid line groups based on Tukey tests ($P > 0.05$); blue cells indicate heterosis (Ht) based on significantly lower value of parameter compared to parental lines based on A or B-lines as indicated in square brackets; red cell indicated heterobeltiosis for susceptibility based on significantly higher parameter value compared to the parental lines based on the A-line (Ht B indicates heterosis with the B-line for the same parameter); †indicates resistance is associated with the restorer line; for statistical results see Table S1

2: Degrees of freedom: accession = 7, nitrogen = 1, interaction = 7, error DF are indicated in the table; numbers are F-values, * = $P \leq 0.05$, ** = $P \leq 0.01$, *** = $P \leq 0.005$

Table S8. Summary of results for BPH effects on hybrid lines (see Table S2 for full details)

Accession	Added Nitrogen (Kg ha-1)[1]	Plant Condition (SES 1-9)[1,2]	Grain Biomass (Dry g)[1]	Shoot Biomass (Dry g)[1]	Root Biomass (Dry g)[1]	Plant Biomass Loss (Dry g)[1,3]	Plant Biomass Loss (Proportion)[1,3]	Plant Biomass Loss per mg of BPH (g mg-BPH^{-1})[1,3]
IR 82396 H	0	5.00±1.03	0.98±0.17ab	2.82±0.28	0.36±0.07	2.58±0.78 [A/B]	0.29±0.08	0.07±0.02
	150	5.67±0.42	1.05±0.16	4.70±0.50	0.56±0.08	7.88±0.55	0.48±0.03	0.11±0.02
IR 82391 H	0	7.00±0.89	0.26±0.12ab	3.18±0.44	0.48±0.07	5.32±0.65	0.56±0.06	0.16±0.02
	150	7.00±1.03	0.71±0.34	3.91±0.84	0.72±0.11	8.58±1.80	0.58±0.11	0.08±0.01
IR 84714 H	0	8.00±1.00	0.25±0.13a	2.78±0.58	0.44±0.06	5.55±1.82	0.52±0.15	0.11±0.02
	150	7.67±0.67	0.50±0.17	5.36±0.86	1.19±0.20	10.69±1.13	0.58±0.05	0.08±0.01
IR 85471 H	0	7.33±0.61	0.41±0.17b	2.64±0.44	0.42±0.10	7.00±0.78	0.62±0.06	0.15±0.00
	150	6.00±1.13	2.56±0.46	5.92±0.49	0.69±0.10	4.15±1.59	0.24±0.10	0.17±0.01
IR 81954 H	0	8.67±0.33	0.85±0.16ab	2.53±0.29	0.26±0.03	4.28±1.22 [A/B]†	0.43±0.09 [B]†	0.10±0.01
	150	5.33±1.09	1.68±0.50	5.09±0.78	0.74±0.11	5.63±2.17	0.32±0.12	0.04±0.01
IR 80637 H	0	4.83±0.91	1.26±0.33ab	2.40±0.41	0.38±0.07	3.98±1.23 [B]†	0.43±0.12 [B]†	0.08±0.01
	150	6.83±0.83	1.21±0.41	3.56±0.43	0.75±0.13	6.09±0.89	0.52±0.06	0.07±0.01
IR 82385 H	0	8.00±1.00	0.49±0.25ab	2.31±0.70	0.79±0.07	5.76±2.17 [A/B]†	0.49±0.17 [A/B]†	0.13±0.00 [B]
	150	6.33±0.67	1.01±0.26	4.90±0.90	0.67±0.12	8.17±0.81	0.50±0.05	0.09±0.01
IR 82363 H	0	6.00±0.86	0.59±0.20ab	2.60±0.26	0.34±0.05	3.98±0.59	0.49±0.06 [A]†	0.07±0.01
	150	7.00±1.26	0.88±0.38	4.76±1.36	0.75±0.24	7.16±2.64	0.48±0.15	0.07±0.01
F-Accession (A)[4]	7	1.549	3.378***	0.670	2.226*	1.325	1.225	1.276
F-Nitrogen (F)[4]	1	0.704	11.460***	40.763***	33.775***	3.273	3.997*	13.770***
F-A×F[4]	7	1.796	2.028	0.904	2.221*	1.518	1.280	0.854
F-Covariate[4]						26.352***	5.284*	12.083***
DF Error			80	80	80	77	79	79

1: Numbers are means ± standard errors (N = 6); lowercase letters indicate homogenous groups based on Tukey tests ($P > 0.05$); blue cells indicate heterosis (Ht) for plant biomass loss based on significantly lower value of parameter compared to parental lines based on A or B-lines as indicated in square brackets; †indicates resistance is associated with the restorer line; heterosis for grain, shoot and root yields after infestation and biomass loss per mg of BPH were not examined (see text for details); for statistical results see Table S2

2: SES = Standard Evaluation System

3: Biomass loss was estimated for whole plants using grain to shoot conversion calculated as Biomass of A-line – Biomass of B-line/Biomass of B-line grain (see text for further details)

4: Degrees of freedom: accession = 7, nitrogen = 1, interaction = 7, covariate (plant biomass equivalent) = 1, error DF are indicated in the table; numbers are F-values, * = $P \leq 0.05$, *** = $P \leq 0.005$

Table S9. Fitness parameters of WBPH on eight hybrid lines and their associated parental lines

Accession	Added Nitrogen (Kg ha-1)[1]	Development Stages (Proportion)[1]						Proportion of Adults that were Female[1]	Proportion of Brachypterous Adults[1]		Total number of WBPH per Plant/Cage[1]	Dry Weight of WBPH per Plant/Cage (mg)[1]
		1st Instar	2nd Instar	3rd Instar	4th Instar	5th Instar	Adults		Females	Males		
IR 80156 A	0	0.01±0.01	0.11±0.06	0.14±0.05	0.12±0.04	0.23±0.08	0.38±0.14ab	0.66±0.08	0.92±0.04b	0.00±0.00	65.17±15.48b	9.60±0.79
	150	0.00±0.00	0.04±0.02	0.13±0.08	0.05±0.03	0.20±0.07	0.58±0.15	0.65±0.08	0.98±0.02	0.13±0.13	98.83±34.46	26.84±6.29
IR 80156 B	0	0.00±0.00	0.00±0.00	0.02±0.01	0.06±0.03	0.28±0.07	0.63±0.09b	0.67±0.08	0.94±0.03b	0.00±0.00	44.33±10.45ab	11.86±2.70
	150	0.00±0.00	0.00±0.00	0.02±0.02	0.06±0.04	0.18±0.03	0.73±0.07	0.51±0.06	0.90±0.05	0.00±0.00	82.00±19.42	25.96±4.77
IR 82396 H	0	0.09±0.04	0.23±0.05	0.31±0.06	0.18±0.04	0.12±0.04	0.08±0.03a	0.23±0.08	0.56±0.29a	0.30±0.20	82.00±19.35ab	11.49±5.25
	150	0.00±0.00	0.00±0.00	0.00±0.00	0.02±0.02	0.54±0.17	0.44±0.16	0.51±0.18	0.65±0.24	0.00±0.00	33.83±24.23	21.13±2.96
IR46 R	0	0.00±0.00	0.00±0.00	0.00±0.00	0.03±0.02	0.37±0.09	0.59±0.10b	0.63±0.08	0.80±0.16ab	0.33±0.33	24.00±6.10a	7.20±0.94
	150	0.00±0.00	0.00±0.00	0.03±0.03	0.01±0.01	0.05±0.03	0.91±0.05	0.44±0.10	1.00±0.00	0.00±0.00	27.33±6.18	10.91±2.05
F-Accession (A)[2]					2.602	1.566	5.948***	1.163	3.008*		4.015*	2.964*
F-Nitrogen (F)[2]					9.194***	0.168	10.366***	0.613	2.324		0.627	20.541***
F-A×F[2]					2.721	6.361***	0.848	1.072	0.986		5.754***	0.642
DF Error					40	40	40	38	29		40	40
IR 79156 A	0	0.00±0.00	0.00±0.00	0.00±0.00	0.10±0.04ab	0.55±0.13	0.36±0.15	0.47±0.08	0.80±0.20	0.03±0.03	23.50±7.11	9.81±0.16ab
	150	0.00±0.00	0.00±0.00	0.04±0.02	0.07±0.03	0.15±0.06	0.74±0.09	0.64±0.09	0.96±0.02	0.00±0.00	78.67±41.67	20.75±5.96
IR 79156 B	0	0.00±0.00	0.01±0.01	0.13±0.08	0.12±0.04b	0.22±0.09	0.52±0.16	0.59±0.08	1.00±0.00	0.00±0.00	49.67±23.23	11.23±2.76b
	150	0.00±0.00	0.03±0.01	0.12±0.05	0.26±0.07	0.37±0.09	0.21±0.05	0.59±0.05	0.90±0.07	0.00±0.00	105.33±27.14	30.16±4.02
IR 82391 H	0	0.00±0.00	0.13±0.09	0.03±0.03	0.09±0.07a	0.23±0.08	0.53±0.18	0.53±0.08	0.93±0.07	0.00±0.00	17.50±6.95	3.71±0.31a
	150	0.00±0.00	0.00±0.00	0.01±0.01	0.03±0.03	0.10±0.05	0.87±0.08	0.61±0.08	0.98±0.02	0.00±0.00	47.00±15.52	15.26±3.45
IR60819-34-2	0	0.00±0.00	0.07±0.05	0.11±0.07	0.14±0.05ab	0.24±0.05	0.44±0.11	0.58±0.08	0.93±0.04	0.00±0.00	49.00±15.39	9.42±2.16ab
	150	0.00±0.00	0.00±0.00	0.00±0.00	0.02±0.02	0.10±0.06	0.88±0.08	0.53±0.05	0.98±0.02	0.00±0.00	39.67±12.22	14.09±3.25
F-Accession (A)[2]					3.104*	2.887*	2.341	0.145	0.170		1.808	4.458**
F-Nitrogen (F)[2]					0.195	5.064*	4.500*	0.245	0.103		5.593*	22.323***
F-A×F[2]					2.959*	4.139*	3.854*	0.299	2.222		1.255	1.553
DF Error					40	40	40	40	34		40	40
IR 80559 A	0	0.11±0.11	0.04±0.02	0.10±0.07	0.07±0.05	0.24±0.07	0.44±0.12	0.43±0.08	0.99±0.01	0.00±0.00	86.83±27.04	15.47±3.17
	150	0.00±0.00	0.00±0.00	0.01±0.01	0.08±0.04	0.15±0.07	0.76±0.11	0.60±0.04	0.94±0.05	0.00±0.00	81.17±27.35	24.78±4.63
IR 80559 B	0	0.00±0.00	0.03±0.02	0.14±0.06	0.09±0.05	0.19±0.06	0.55±0.15	0.58±0.08	0.94±0.04	0.00±0.00	80.50±31.40	15.87±3.90
	150	0.00±0.00	0.00±0.00	0.00±0.00	0.00±0.00	0.00±0.00	1.00±0.00	0.66±0.05	1.00±0.00	0.00±0.00	44.17±13.57	19.13±5.69
IR 84714 H	0	0.00±0.00	0.00±0.00	0.06±0.04	0.05±0.05	0.19±0.05	0.69±0.11	0.50±0.08	0.93±0.07	0.00±0.00	30.83±14.80	8.04±3.40
	150	0.00±0.00	0.03±0.02	0.10±0.05	0.12±0.03	0.26±0.04	0.50±0.08	0.64±0.10	1.00±0.00	0.00±0.00	92.67±25.56	23.90±6.12

IR60819-34-2 R	0	0.00±0.00	0.00±0.00	0.05±0.03	0.14±0.07	0.27±0.08	0.54±0.14	0.66±0.08	0.68±0.19	0.00±0.00	51.83±19.28	12.03±5.31
	150	0.00±0.00	0.00±0.00	0.03±0.02	0.08±0.04	0.23±0.09	0.65±0.14	0.57±0.05	0.93±0.07	0.00±0.00	57.83±16.62	16.55±3.72
F-Accession (A)²					0.708	2.250	2.635	0.672	1.502		0.976	1.051
F-Nitrogen (F)²					0.292	2.018	6.117**	0.693	2.176		1.016	7.368***
F-A×F²					1.219	1.340	3.839*	1.302	1.187		1.993	0.355
DF Error					40	40	40	40	39		40	40
IR 80564 A	0	0.04±0.02	0.11±0.05	0.11±0.05	0.10±0.05ab	0.16±0.04	0.47±0.19	0.48±0.08	0.65±0.21	0.00±0.00	62.33±20.25	9.84±1.83
	150	0.00±0.00	0.05±0.04	0.07±0.04	0.08±0.04	0.32±0.10	0.49±0.13	0.49±0.11	0.90±0.04	0.00±0.00	64.83±16.03	14.91±3.40
IR 80564 B	0	0.00±0.00	0.05±0.05	0.06±0.06	0.29±0.07b	0.26±0.07	0.34±0.14	0.64±0.08	0.97±0.03	0.00±0.00	43.50±13.32	8.91±0.81
	150	0.00±0.00	0.01±0.01	0.08±0.08	0.13±0.06	0.20±0.06	0.58±0.14	0.61±0.08	0.82±0.09	0.00±0.00	64.50±32.98	13.52±3.19
IR 85471 H	0	0.00±0.00	0.13±0.05	0.20±0.09	0.07±0.03a	0.18±0.07	0.39±0.18	0.37±0.08	1.00±0.00	0.15±0.15	135.50±73.95	8.85±2.04
	150	0.00±0.00	0.01±0.01	0.05±0.03	0.06±0.03	0.15±0.06	0.73±0.10	0.54±0.05	0.99±0.01	0.00±0.00	54.50±23.44	18.64±7.53
IR60819-34-2 R	0	0.00±0.00	0.00±0.00	0.05±0.03	0.14±0.07ab	0.27±0.08	0.54±0.14	0.66±0.08	0.68±0.19	0.00±0.00	51.83±19.28	12.03±5.31
	150	0.00±0.00	0.00±0.00	0.05±0.02	0.08±0.04	0.24±0.09	0.63±0.15	0.55±0.05	0.87±0.08	0.00±0.00	53.33±17.00	16.55±3.72
F-Accession (A)²				0.694	3.286*	0.583	0.271	1.978	2.443		0.403	0.114
F-Nitrogen (F)²				1.273	2.847	0.052	1.414	0.025	0.016		0.005	4.215*
F-A×F²				1.065	0.896	0.945	0.245	1.173	0.965		0.571	0.105
DF Error				40.00	40.00	40.00	40.00	40.00	40.00		40.00	40.00
IR 70369 A	0	0.00±0.00	0.12±0.07	0.15±0.07	0.10±0.05	0.10±0.05a	0.52±0.21	0.57±0.08	0.98±0.02	0.20±0.20	136.50±62.48	10.91±0.26ab
	150	0.00±0.00	0.00±0.00	0.02±0.02	0.06±0.03	0.15±0.05	0.78±0.08	0.53±0.05	0.73±0.17	0.00±0.00	64.33±19.98	20.30±4.46
IR 70369 B	0	0.00±0.00	0.00±0.00	0.03±0.03	0.06±0.04	0.44±0.08b	0.47±0.06	0.62±0.08	0.93±0.07	0.00±0.00	60.00±10.58	15.36±1.09b
	150	0.00±0.00	0.01±0.01	0.03±0.03	0.05±0.02	0.24±0.09	0.67±0.12	0.47±0.04	0.85±0.13	0.00±0.00	86.67±14.32	27.67±4.43
IR 81954 H	0	0.00±0.00	0.02±0.01	0.05±0.03	0.12±0.05	0.46±0.08ab	0.35±0.07	0.44±0.08	0.92±0.06	0.00±0.00	42.50±17.18	8.84±2.12a
	150	0.00±0.00	0.00±0.00	0.05±0.05	0.04±0.04	0.11±0.05	0.80±0.13	0.64±0.04	0.94±0.06	0.00±0.00	51.50±22.27	14.44±3.60
IR72889-46-3-2-1 R	0	0.00±0.00	0.02±0.02	0.05±0.05	0.14±0.05	0.17±0.09a	0.62±0.18	0.47±0.08	1.00±0.00	0.00±0.00	50.50±23.57	9.89±2.20ab
	150	0.01±0.01	0.03±0.03	0.08±0.06	0.05±0.04	0.06±0.04	0.77±0.15	0.64±0.08	0.90±0.04	0.01±0.01	99.00±42.51	21.52±7.22
F-Accession (A)²				0.439	0.389	5.284***	1.074	0.017	0.199		1.214	2.809*
F-Nitrogen (F)²				0.665	3.730	10.074***	6.177*	0.018	3.111		0.406	8.450**
F-A×F²				1.322	0.403	2.935*	0.484	1.536	0.980		0.538	0.013
DF Error				40	40	40	40	39	39		40	40
IR 73328 A	0	0.00±0.00	0.00±0.00	0.00±0.00	0.02±0.02a	0.14±0.07a	0.84±0.07b	0.60±0.08ab	1.00±0.00	0.07±0.07	26.60±8.50	8.71±2.36a
	150	0.00±0.00	0.00±0.00	0.02±0.02	0.02±0.02	0.07±0.06	0.88±0.11	0.63±0.07	0.93±0.06	0.00±0.00	67.00±25.39	21.34±3.79
IR 73328 B	0	0.00±0.00	0.00±0.00	0.01±0.01	0.04±0.03ab	0.13±0.06a	0.82±0.09b	0.62±0.08b	0.92±0.07	0.00±0.00	41.17±8.77	16.82±1.13a
	150	0.00±0.00	0.00±0.00	0.01±0.01	0.02±0.02	0.05±0.04	0.91±0.07	0.68±0.09	0.98±0.02	0.00±0.00	38.67±13.57	14.53±3.22
IR 80637 H	0	0.00±0.00	0.02±0.02	0.07±0.05	0.09±0.04b	0.37±0.11b	0.44±0.13a	0.48±0.08ab	0.85±0.08	0.00±0.00	61.17±28.24	18.48±1.63b

	150	0.00±0.00	0.00±0.00	0.04±0.03	0.14±0.05	0.25±0.08	0.57±0.14	0.60±0.05	0.85±0.13	0.00±0.00	99.17±13.58	34.30±1.65
IR73013-95-1-3-2 R	0	0.00±0.00	0.04±0.04	0.11±0.07	0.07±0.05ab	0.26±0.10ab	0.52±0.18ab	0.39±0.08a	1.00±0.00	0.00±0.00	57.83±33.56	12.64±2.48ab
	150	0.00±0.00	0.00±0.00	0.02±0.02	0.06±0.03	0.19±0.05	0.73±0.08	0.48±0.04	0.97±0.03	0.14±0.14	94.83±23.41	22.13±2.34
F-Accession (A)[2]					2.909*	4.009*	4.516**	2.833*	1.623		1.080	5.784**
F-Nitrogen (F)[2]					0.010	2.777	1.962	1.504	0.046		5.775*	14.619***
F-A×F[2]					0.351	0.100	0.017	0.138	0.678		1.572	4.121*
DF Error					40	40	40	39	38		39	40
IR 79125 A	0	0.00±0.00	0.06±0.06	0.03±0.03	0.00±0.00	0.22±0.14	0.69±0.14ab	0.50±0.08	0.90±0.10	0.00±0.00	7.67±1.12a	3.06±0.06a
	150	0.00±0.00	0.00±0.00	0.12±0.08	0.07±0.05	0.17±0.06	0.63±0.12	0.63±0.15	0.93±0.07	0.00±0.00	18.83±7.91	6.16±1.01
IR 79125 B	0	0.09±0.06	0.15±0.05	0.21±0.07	0.23±0.13	0.17±0.09	0.16±0.15a	0.88±0.08	0.73±0.27	0.00±0.00	37.67±12.49b	6.19±1.10b
	150	0.00±0.00	0.00±0.00	0.06±0.05	0.12±0.04	0.34±0.05	0.47±0.10	0.49±0.05	0.93±0.04	0.00±0.00	99.17±29.09	25.63±3.42
IR 82385 H	0	0.00±0.00	0.02±0.02	0.04±0.04	0.05±0.04	0.21±0.09	0.68±0.15b	0.53±0.08	0.90±0.08	0.00±0.00	49.17±13.72ab	11.15±1.64b
	150	0.00±0.00	0.00±0.00	0.00±0.00	0.01±0.01	0.05±0.05	0.94±0.06	0.64±0.07	0.95±0.04	0.00±0.00	23.17±10.23	7.35±1.69
IR73717-46-1-3-3 R	0	0.00±0.00	0.00±0.00	0.00±0.00	0.24±0.05	0.38±0.11	0.37±0.10ab	0.52±0.08	0.75±0.19	0.00±0.00	29.50±10.78b	8.13±1.56b
	150	0.00±0.00	0.00±0.00	0.04±0.04	0.11±0.07	0.17±0.08	0.67±0.16	0.51±0.10	1.00±0.00	0.00±0.00	58.83±25.47	15.86±4.57
F-Accession (A)[2]					3.355*	1.001	5.632***	1.285	0.260		5.180***	12.137***
F-Nitrogen (F)[2]					1.567	1.221	4.015*	0.789	2.705		1.022	18.150***
F-A×F[2]					1.104	1.807	1.439	2.887*	0.686		2.496	9.784***
DF Error					40	40	40	37	34		40	40
IR 68897 A	0	0.00±0.00	0.00±0.00	0.00±0.00	0.03±0.02	0.48±0.05	0.48±0.04	0.64±0.08	0.91±0.06	0.00±0.00	10.33±1.33a	3.14±0.19a
	150	0.00±0.00	0.00±0.00	0.04±0.04	0.05±0.04	0.17±0.08	0.74±0.13	0.70±0.06	0.88±0.08	0.00±0.00	47.17±16.01	15.00±2.66
IR 68897 B	0	0.00±0.00	0.00±0.00	0.06±0.04	0.04±0.03	0.20±0.05	0.70±0.06	0.63±0.08	1.00±0.00	0.00±0.00	23.00±4.89ab	9.67±0.63ab
	150	0.00±0.00	0.00±0.00	0.08±0.05	0.08±0.05	0.30±0.08	0.51±0.14	0.59±0.12	1.00±0.00	0.00±0.00	86.33±32.73	15.30±3.85
IR 82363 H	0	0.00±0.00	0.00±0.00	0.08±0.05	0.11±0.06	0.49±0.07	0.32±0.08	0.55±0.08	0.74±0.16	0.00±0.00	48.67±27.89ab	25.66±10.21b
	150	0.00±0.00	0.00±0.00	0.04±0.04	0.01±0.01	0.17±0.06	0.78±0.08	0.63±0.07	1.00±0.00	0.11±0.11	59.50±5.73	22.99±6.96
SRT 3 R	0	0.11±0.09	0.11±0.06	0.11±0.06	0.09±0.04	0.16±0.06	0.43±0.18	0.68±0.08	0.89±0.07	0.00±0.00	83.00±33.97b	12.55±2.94ab
	150	0.00±0.00	0.00±0.00	0.05±0.05	0.08±0.05	0.35±0.06	0.51±0.10	0.52±0.05	0.97±0.02	0.00±0.00	70.33±11.44	18.17±2.24
F-Accession (A)[2]					0.382	0.986	0.659	0.663	1.517		4.140*	4.226*
F-Nitrogen (F)[2]					0.187	3.412	4.848*	0.021	2.361		10.694***	9.056***
F-A×F[2]					0.971	8.343***	2.704	0.648	1.584		0.825	1.743
DF Error					40	40	40	40	40		40	40

1: Numbers are means ± standard errors (N = 6); lowercase letters indicate homogenous groups based on Tukey tests (P > 0.05); too few individuals were recorded to include 1st, 2nd and 3rd instars in some analyses and no analyses were conducted on the proportions of brachypterous males because of low variability

2: Degrees of freedom: accession = 7, nitrogen = 1, interaction = 7, error DF are indicated in the table; numbers are F-values, * = P ≤ 0.05, ** = P ≤ 0.01, *** = P ≤ 0.005

Table S10. Plant biomass and estimated weight losses for eight hybrid lines and their associated parental lines after infestation with WBPH (see Table 5)

Accession	Added Nitrogen (Kg ha-1)[1]	Plant Condition (Proportion Alive)[1,2]	Grain Biomass (Dry g)[1]	Shoot Biomass (Dry g)[1]	Root Biomass (Dry g)[1]	Plant Biomass Loss (Dry g)[1,3]	Plant Biomass Loss (Proportion)[1,3]	Plant Biomass Loss per mg of WBPH (g mg-BPH^{-1})[1,3]
IR 80156 A	0	1.00±0.00		4.71±0.59c	0.56±0.09	2.50±0.99	0.29±0.10a	0.29±0.11
	150	0.83±0.17		8.76±1.25	1.01±0.21	3.74±0.69	0.29±0.06	0.18±0.07
IR 80156 B	0	1.00±0.00	1.12±0.16	2.21±0.31a	0.35±0.07	5.66±0.82	0.68±0.05ab	0.78±0.35
	150	1.00±0.00	1.93±0.54	4.18±0.20	0.63±0.11	3.32±1.02	0.23±0.07	0.24±0.14
IR 82396 H	0	0.83±0.17	1.09±0.15	3.20±0.31bc	0.59±0.08	4.59±0.46	0.55±0.04ab	0.71±0.18
	150	1.00±0.00	1.67±0.36	7.14±0.63	0.94±0.14	3.13±1.39	0.19±0.08	0.15±0.08
IR46 R	0	1.00±0.00	1.10±0.10	3.23±0.27b	0.54±0.10	5.90±0.41	0.60±0.04b	0.90±0.14
	150	1.00±0.00	1.43±0.34	5.79±0.85	0.71±0.11	5.05±1.29	0.30±0.07	0.60±0.25
F-Accession (A)[4]		0.667	0.290	11.974***	2.883*	2.502	3.917*	2.812
F-Nitrogen (F)[4]		0.000	3.738>	58.620***	14.207***	3.175	0.342	8.201**
F-A×F[4]		1.333	0.304	0.438	0.350	0.509	2.517	8.751
F-Covariate[4]						9.059***	30.556***	
DF Error		40	30	40	40	39	39	40
IR 79156 A	0	1.00±0.00		6.13±0.54c	0.99±0.16b	1.54±0.46	0.01±0.29	0.16±0.15
	150	1.00±0.00		6.84±0.37	0.60±0.07	2.42±1.54	0.15±0.13	0.41±0.34
IR 79156 B	0	1.00±0.00	1.39±0.24	2.43±0.30a	0.40±0.08a	2.24±0.07	0.23±0.12	0.22±0.16
	150	0.83±0.17	1.24±0.27	2.40±0.60	0.23±0.08	7.64±1.77	0.65±0.10	0.29±0.08
IR 82391 H	0	1.00±0.00	0.97±0.15	3.42±0.55bc	0.60±0.03b	3.19±0.46	0.34±0.05	0.89±0.14
	150	1.00±0.00	0.96±0.16	7.36±0.83	0.88±0.18	3.79±1.63	0.26±0.12	0.42±0.21
IR60819-34-2 R	0	1.00±0.00	1.12±0.20	2.75±0.33b	0.37±0.04b	3.75±0.18	0.33±0.09	0.53±0.17
	150	1.00±0.00	2.19±0.64	4.86±0.53	0.78±0.16	3.52±1.55	0.25±0.11	0.42±0.22
F-Accession (A)[4]		1.000	1.824	24.740***	9.167***	0.776	1.581	1.774
F-Nitrogen (F)[4]		1.000	0.698	16.199***	0.053	4.986*	2.654	0.227
F-A×F[4]		1.000	1.546	5.150***	5.508***	0.418	0.077	1.196
F-Covariate[4]						7.551**	12.654***	
DF Error		40	30	40	40	39	39	40
IR 80559 A	0	0.83±0.17		4.36±0.53b	0.44±0.09	1.56±0.95a	0.19±0.14a	0.10±0.08a
	150	0.83±0.17		8.12±1.29	0.89±0.17	0.93±1.44	0.03±0.13	0.03±0.05
IR 80559 B	0	0.67±0.21	0.00±0.00a	4.66±0.53b	0.60±0.15	0.13±0.83ab	(-)0.07±0.20a	0.11±0.16ab
	150	0.83±0.17	0.00±0.00	6.91±1.35	0.74±0.25	3.61±2.84	0.14±0.33	0.39±0.24
IR 84714 H	0	1.00±0.00	1.09±0.15b	5.17±0.40b	0.53±0.06	0.97±0.58ab	(-)0.01±0.20ab	0.26±0.48ab

	150	1.00±0.00	0.95±0.06	8.22±0.41	1.01±0.17	6.91±1.24	0.36±0.05	0.35±0.07
IR60819-34-2 R	0	1.00±0.00	1.49±0.29c	3.18±0.39a	0.59±0.04	4.31±0.84b	0.32±0.13b	0.59±0.39b
	150	1.00±0.00	1.83±0.25	4.15±0.66	0.50±0.13	7.98±1.49	0.46±0.08	0.66±0.26
F-Accession (A)[4]		1.957	49.077***	7.855***	0.775	14.695***	7.414***	3.539*
F-Nitrogen (F)[4]		0.217	0.207	19.488***	4.552*	40.666***	17.490***	4.225*
F-A×F[4]		0.217	1.088	0.804	1.932	1.859	1.034	0.131
F-Covariate[4]						37.151***	31.056***	9.112***
DF Error		40	30	40	40	39	39	39
IR 80564 A	0	1.00±0.00		4.18±0.37c	0.41±0.02ab	2.80±0.72ab	0.25±0.24ab	0.35±0.21ab
	150	1.00±0.00		6.74±1.13	0.70±0.14	4.98±1.34	0.40±0.11	0.36±0.12
IR 80564 B	0	1.00±0.00	1.80±0.23b	2.13±0.22a	0.38±0.07a	0.59±0.23a	0.01±0.14a	0.08±0.14a
	150	1.00±0.00	2.41±0.41	3.04±0.39	0.43±0.07	4.40±1.88	0.31±0.12	0.16±0.23
IR 85471 H	0	0.83±0.17	0.88±0.15a	4.09±0.27bc	0.61±0.08b	3.91±0.67ab	0.34±0.04ab	0.51±0.08ab
	150	1.00±0.00	1.56±0.27	6.00±0.76	0.74±0.08	6.26±1.01	0.37±0.05	0.67±0.21
IR60819-34-2 R	0	1.00±0.00	1.49±0.29ab	3.18±0.39ab	0.59±0.04ab	4.58±0.18b	0.31±0.14b	0.62±0.45b
	150	1.00±0.00	1.83±0.25	4.15±0.66	0.50±0.13	7.61±1.40	0.46±0.08	0.62±0.24
F-Accession (A)[4]		1.000	5.888**	11.866***	3.150*	10.048***	8.841***	3.991*
F-Nitrogen (F)[4]		1.000	6.421*>	12.983***	1.762	41.883***	29.745***	2.451
F-A×F[4]		1.000	0.350	0.215	1.585	0.378	0.482	0.404
F-Covariate[4]						84.010***	80.117***	15.087***
DF Error		40.00	30.00	40.00	40.00	39	39	39
IR 70369 A	0	1.00±0.00		4.60±0.76b	0.50±0.10	4.43±0.78	0.46±0.09	0.40±0.07
	150	0.83±0.17		5.63±1.03	0.94±0.13	6.32±2.41	0.41±0.15	0.18±0.19
IR 70369 B	0	0.83±0.17	1.64±0.16b	3.45±0.26ab	0.63±0.08	1.39±0.64	0.14±0.07	0.08±0.04
	150	0.83±0.17	1.58±0.20	4.05±0.17	0.71±0.10	5.25±3.01	0.14±0.21	0.35±0.26
IR 81954 H	0	1.00±0.00	1.30±0.27a	3.21±0.51ab	0.40±0.06	2.37±0.52	0.18±0.18	0.24±0.24
	150	1.00±0.00	2.80±0.63	4.59±0.27	0.52±0.06	2.77±2.20	0.14±0.11	0.22±0.16
IR72889-46-3-2-1 R	0	1.00±0.00	1.43±0.22ab	2.49±0.21a	0.48±0.09	2.19±0.65	0.25±0.07	0.28±0.13
	150	1.00±0.00	1.17±0.38	3.94±0.62	0.62±0.10	9.73±3.98	0.41±0.17	1.10±0.55
F-Accession (A)[4]		1.222	3.140	3.190*	3.049*	0.202	0.526	1.389
F-Nitrogen (F)[4]		0.333	2.783	8.753***	8.814*	15.162***	4.748	5.028*
F-A×F[4]		0.330	0.089	0.386	1.358	0.598	0.584	1.448
F-Covariate[4]						12.692***	18.773***	5.621*
DF Error		40	30	40	40	39	39	39
IR 73328 A	0	1.00±0.00		5.39±0.43b	0.52±0.09	3.21±0.14	0.32±0.10	0.38±0.13

	150	1.00±0.00		7.53±0.89	0.66±0.13	3.86±0.86	0.32±0.07	0.20±0.04
IR 73328 B	0	1.00±0.00	1.07±0.25	2.80±0.53a	0.38±0.09	4.48±0.31	0.46±0.13	0.25±0.07
	150	1.00±0.00	1.78±0.58	4.73±0.96	0.53±0.14	4.88±1.58	0.40±0.12	0.29±0.11
IR 80637 H	0	1.00±0.00	1.63±0.42	2.75±0.39a	0.48±0.07	2.94±0.02	0.33±0.12	0.16±0.05
	150	1.00±0.00	2.15±0.71	4.29±0.65	0.55±0.12	4.52±1.37	0.39±0.12	0.14±0.04
IR73013-95-1-3-2 R	0	0.67±0.21	1.42±0.21	3.04±0.39a	0.51±0.05	4.29±0.05	0.41±0.10	0.61±0.37
	150	1.00±0.00	2.41±0.37	5.24±0.32	0.74±0.08	4.35±0.83	0.33±0.06	0.22±0.06
F-Accession (A)[4]		2.500	0.923	8.286***	1.245	1.008	0.908	1.760
F-Nitrogen (F)[4]		2.500	2.850>	18.695***	3.434	18.047***	16.934***	0.022
F-A×F[4]		2.500	0.175	0.215	0.204	0.023	0.104	0.781
F-Covariate[4]						55.597***	121.149***	7.270*
DF Error		40	30	40	40	39	39	39
IR 79125 A	0	1.00±0.00		6.22±0.73b	0.67±0.09	3.97±0.63	0.36±0.05	1.29±0.20
	150	1.00±0.00		10.55±0.96	1.10±0.20	2.59±1.18	0.17±0.08	0.45±0.23
IR 79125 B	0	1.00±0.00	1.84±0.19b	3.07±0.39a	0.44±0.08	1.77±0.91	0.16±0.08	0.26±0.16
	150	0.83±0.17	2.54±0.24	5.52±0.71	0.82±0.13	0.77±7.93	-1.70±1.90	-0.05±0.33
IR 82385 H	0	0.83±0.17	0.75±0.23a	3.20±0.51a	0.49±0.10	4.36±0.97	0.44±0.10	0.38±0.08
	150	1.00±0.00	1.52±0.42	6.80±0.80	1.10±0.25	2.65±4.52	0.01±0.21	-0.01±1.36
IR73717-46-1-3-3 R	0	1.00±0.00	1.47±0.15b	3.18±0.45a	0.70±0.09	2.49±0.08	0.21±0.10	0.29±0.13
	150	1.00±0.00	1.85±0.24	5.70±1.01	0.77±0.13	0.59±2.17	-0.01±0.09	0.00±0.16
F-Accession (A)[4]		0.667	9.782***	10.942***	1.278	1.506	0.412	0.914
F-Nitrogen (F)[4]		0.001	7.278*>	37.373***	14.325***	9.339***	2.383	7.274**
F-A×F[4]		1.333	0.483	0.295	1.349	1.821	0.458	2.743
F-Covariate[4]						16.387***	7.498**	16.839***
DF Error		40	40	40	40	39	39	39
IR 68897 A	0	1.00±0.00		6.83±0.59c	0.82±0.11b	0.27±0.65	(-)0.13±0.28a	0.02±0.58
	150	0.83±0.17		10.33±0.54	0.76±0.05	2.08±1.54	0.10±0.10	0.32±0.25
IR 68897 B	0	1.00±0.00	1.90±0.26	2.53±0.15ab	0.34±0.03a	1.41±0.58	0.06±0.20a	0.12±0.18
	150	1.00±0.00	1.77±0.36	3.67±0.57	0.48±0.16	5.16±1.38	0.36±0.10	0.57±0.28
IR 82363 H	0	1.00±0.00	1.39±0.15	2.92±0.21b	0.45±0.05ab	1.57±0.95	0.14±0.11ab	0.07±0.10
	150	1.00±0.00	2.55±0.15	5.23±0.78	0.71±0.15	2.49±1.82	0.12±0.12	0.20±0.11
SRT 3 R	0	1.00±0.00	0.88±0.18	1.92±0.20a	0.25±0.04a	6.65±0.88	0.58±0.06b	0.85±0.30
	150	1.00±0.00	1.62±0.36	3.25±0.42	0.49±0.09	7.06±1.65	0.46±0.11	0.46±0.15
F-Accession (A)[4]		1.000	5.297*	49.143***	8.899***	1.721	1.300	0.002
F-Nitrogen (F)[4]		1.000	6.900*>	31.909***	4.774*	12.659***	10.000***	7.498**

F-A×F[4]	1.000	3.030	0.390	1.067	0.305	0.848	1.183
F-Covariate[4]					11.578***	14.959***	13.122***
DF Error	40	30	40	40	39	39	39

1: Numbers are means ± standard errors (N = 6); lowercase letters indicate homogenous groups based on Tukey tests (P > 0.05)

2: Condition was not assessed using SES due to poor resolution of the system for WBPH

3: Biomass loss was estimated for whole plants using grain to shoot conversion calculated as Biomass of A-line – Biomass of B-line/Biomass of B-line grain (see text for further details)

4: Degrees of freedom: accession = 7, nitrogen = 1, interaction = 7, covariate (plant biomass equivalent) = 1, error DF are indicated in the table; numbers are F-values, * = P ≤ 0.05, ** = P ≤ 0.01, *** = P ≤ 0.005

Table S11. Summary of results for WBPH fitness on hybrid lines (see Table S5 for full details)

Accession	Added Nitrogen (Kg ha^{-1})[1]	Development Stages (Proportion)[1]						Proportion of Adults that were Female[1]	Proportion of Brachypterous Adults[1]		Total number of WBPH per Plant/Cage[1]	Dry Weight of WBPH per Plant/Cage (mg)[1]
		1st Instar	2nd Instar	3rd Instar	4th Instar	5th Instar	Adults		Females	Males		
IR 82396 H	0	0.09±0.04	0.23±0.05	0.31±0.06	0.18±0.04	0.12±0.04	0.08±0.03a [Hb B/Ht A]	0.23±0.08	0.56±0.29 [Hb A/B]	0.30±0.20	82.00±19.35 [A/B]†	11.49±5.25ab [A/B]†
	150	0.00±0.00	0.00±0.00	0.00±0.00	0.02±0.02	0.54±0.17	0.44±0.16	0.51±0.18	0.65±0.24	0.00±0.00	33.83±24.23	21.13±2.96
IR 82391 H	0	0.00±0.00	0.13±0.09	0.03±0.03	0.09±0.07 [B]	0.23±0.08 [A]	0.53±0.18b	0.53±0.08	0.93±0.07	0.00±0.00	17.50±6.95	3.71±0.31a [B]
	150	0.00±0.00	0.00±0.00	0.01±0.01	0.03±0.03	0.10±0.05	0.87±0.08	0.61±0.08	0.98±0.02	0.00±0.00	47.00±15.52	15.26±3.45
IR 84714 H	0	0.00±0.00	0.00±0.00	0.06±0.04	0.05±0.05	0.19±0.05	0.69±0.11ab	0.50±0.08	0.93±0.07	0.00±0.00	30.83±14.80	8.04±3.40a
	150	0.00±0.00	0.03±0.02	0.10±0.05	0.12±0.03	0.26±0.04	0.50±0.08	0.64±0.10	1.00±0.00	0.00±0.00	92.67±25.56	23.90±6.12
IR 85471 H	0	0.00±0.00	0.13±0.05	0.20±0.09	0.07±0.03 [B]	0.18±0.07	0.39±0.18ab	0.37±0.08	1.00±0.00	0.15±0.15	135.50±73.95	8.85±2.04a
	150	0.00±0.00	0.01±0.01	0.05±0.03	0.06±0.03	0.15±0.06	0.73±0.10	0.54±0.05	0.99±0.01	0.00±0.00	54.50±23.44	18.64±7.53
IR 81954 H	0	0.00±0.00	0.02±0.01	0.05±0.03	0.12±0.05	0.46±0.08 [B]	0.35±0.07ab	0.44±0.08	0.92±0.06	0.00±0.00	42.50±17.18	8.84±2.12a [B]
	150	0.00±0.00	0.00±0.00	0.05±0.05	0.04±0.04	0.11±0.05	0.80±0.13	0.64±0.04	0.94±0.06	0.00±0.00	51.50±22.27	14.44±3.60
IR 80637 H	0	0.00±0.00	0.02±0.02	0.07±0.05	0.09±0.04 [A]	0.37±0.11 [A/B]	0.44±0.13ab [A/B]	0.48±0.08	0.85±0.08	0.00±0.00	61.17±28.24	18.48±1.63b [A/B]
	150	0.00±0.00	0.00±0.00	0.04±0.03	0.14±0.05	0.25±0.08	0.57±0.14	0.60±0.05	0.85±0.13	0.00±0.00	99.17±13.58	34.30±1.65
IR 82385 H	0	0.00±0.00	0.02±0.02	0.04±0.04	0.05±0.04	0.21±0.09	0.68±0.15b [B]	0.53±0.08	0.90±0.08	0.00±0.00	49.17±13.72	11.15±1.64a [A]
	150	0.00±0.00	0.00±0.00	0.00±0.00	0.01±0.01	0.05±0.05	0.94±0.06	0.64±0.07	0.95±0.04	0.00±0.00	23.17±10.23	7.35±1.69
IR 82363 H	0	0.00±0.00	0.00±0.00	0.08±0.05	0.11±0.06	0.49±0.07	0.32±0.08ab	0.55±0.08	0.74±0.16	0.00±0.00	48.67±27.89	25.66±10.21ab
	150	0.00±0.00	0.00±0.00	0.04±0.04	0.01±0.01	0.17±0.06	0.78±0.08	0.63±0.07	1.00±0.00	0.11±0.11	59.50±5.73	22.99±6.96
F-Accession (A)[2]					0.912	2.243*	3.619***	0.718	2.609*		1.197	4.259***
F-Nitrogen (F)[2]					4.208*	1.756	16.355***	5.165*	2.888		0.247	17.544***
F-A×F[2]					1.687	4.998***	1.406	0.056	0.542		4.167***	2.049
DF Error					80	80	80	80	72		80	80

1: Numbers are means ± standard errors (N = 6); lowercase letters indicate homogenous hybrid line groups based on Tukey tests (P > 0.05); blue cells indicate heterosis (Ht) based on significantly lower value of parameter compared to parental lines based on A or B-lines as indicated in square brackets; green cells indicate heterobeltiosis for resistance based on significantly higher parameter values compared to the parental lines based on the A-line (for adults) and A- and B-lines (for brachypterous females) (Ht A indicates heterosis with the A-line for development to adults); †indicates resistance is associated with the restorer line; for statistical results see Table S5

2: Degrees of freedom: accession = 7, nitrogen = 1, interaction = 7, error DF are indicated in the table; numbers are F-values, * = P ≤ 0.05, *** = P ≤ 0.005

Table S12. Summary of results for WBPH effects on hybrid lines (see Table S6 for full details)

Accession	Added Nitrogen (Kg ha-1)[1]	Plant Condition (Proportion Alive)[1,2]	Grain Biomass (Dry g)[1]	Shoot Biomass (Dry g)[1]	Root Biomass (Dry g)[1]	Plant Biomass Loss (Dry g)[1,3]	Plant Biomass Loss (Proportion)[1,3]	Plant Biomass Loss per mg of BPH (g mg-WBPH^{-1})[1,3]
IR 82396 H	0	0.83±0.17	1.09±0.15ab	3.20±0.31abc	0.59±0.08b	4.59±0.46ab	0.55±0.04b	0.71±0.18
	150	1.00±0.00	1.67±0.36	7.14±0.63	0.94±0.14	3.13±150.39	0.19±0.08	0.15±0.08
IR 82391 H	0	1.00±0.00	0.97±0.15a	3.42±0.55bc	0.60±0.03	3.19±0.46ab	0.34±0.05ab	0.89±0.14
	150	1.00±0.00	0.96±0.16	7.36±0.83	0.88±0.18	3.79±150.63	0.26±0.12	0.42±0.21
IR 84714 H	0	1.00±0.00	1.09±0.15ab	5.17±0.40c	0.53±0.06b	0.97±0.58ab	-0.01±0.20ab	0.26±0.48
	150	1.00±0.00	0.95±0.06	8.22±0.41	1.01±0.17	6.91±150.24	0.36±0.05	0.35±0.07
IR 85471 H	0	0.83±0.17	0.88±0.15ab	4.09±0.27abc	0.61±0.08ab	3.91±0.67b	0.34±0.04ab	0.51±0.08
	150	1.00±0.00	1.56±0.27	6.00±0.76	0.74±0.08	6.26±150.01	0.37±0.05	0.67±0.21
IR 81954 H	0	1.00±0.00	1.30±0.27ab	3.21±0.51ab	0.40±0.06a	2.37±0.52ab	0.18±0.18ab	0.24±0.24
	150	1.00±0.00	2.80±0.63	4.59±0.27	0.52±0.06	2.77±150.20	0.14±0.11	0.22±0.16
IR 80637 H	0	1.00±0.00	1.63±0.42ab	2.75±0.39a	0.48±0.07ab	2.94±0.02ab	0.33±0.12ab	0.16±0.05
	150	1.00±0.00	2.15±0.71	4.29±0.65	0.55±0.12	4.52±150.37	0.39±0.12	0.14±0.04
IR 82385 H	0	0.83±0.17	0.75±0.23ab	3.20±0.51abc	0.49±0.10ab	4.36±0.97ab	0.44±0.10ab	0.38±0.08
	150	1.00±0.00	1.52±0.42	6.80±0.80	1.10±0.25	2.65±150.52	0.01±0.21	-0.01±1.36
IR 82363 H	0	1.00±0.00	1.39±0.15b	2.92±0.21ab	0.45±0.05ab	1.57±0.95a	0.14±0.11a	0.07±0.10
	150	1.00±0.00	2.55±0.15	5.23±0.78	0.71±0.15	2.49±150.82	0.12±0.12	0.20±0.11
F-Accession (A)[4]			3.299***	5.929***	2.424*	2.659*	2.628*	1.359
F-Nitrogen (F)[4]			12.924***	88.362***	23.153***	36.309***	20.938***	8.988***
F-A×F[4]			1.154	1.239	1.117	1.625	1.666	1.073
F-Covariate[4]						46.711***	57.837***	24.305***
DF Error			80	80	80	79	79	79

1: Numbers are means ± standard errors (N = 6); lowercase letters indicate homogenous groups based on Tukey tests ($P > 0.05$); there were no cases of heterosis or heterobeltiosis; for statistical results see Table S6

2: Condition was not assessed using SES due to poor resolution of the system for WBPH 3: Biomass loss was estimated for whole plants using grain to shoot conversion calculated as Biomass of A-line – Biomass of B-line/Biomass of B-line grain (see text for further details)

4: Degrees of freedom: accession = 7, nitrogen = 1, interaction = 7, covariate (plant biomass equivalent) = 1, error DF are indicated in the table; numbers are F-values, * = $P \leq 0.05$, *** = $P \leq 0.005$

Table S13. Fitness parameters of YSB on eight hybrid lines and their associated parental lines

Accession	Added Nitrogen ($Kg\ ha^{-1}$)	Number of Replicated Blocks	Number of Female Moths Emerged[1]	Time to Adult Female Emergence (Days)[1]	Adult Female Biomass (Dry mg)[1]	Number of Male Moths Emerged[1]	Time to Adult Male Emergence (Days)[1]	Adult Male Biomass (Dry mg)[1]	Total Number of Emerged Adults[1]	Biomass of Emerged Adults[1] (Dry mg)
IR 80156 A	0.00	2	1.50±0.50	35.25±5.25	7.38±0.83	0.50±0.50	30.00	6.10±	2.00±0.00	13.70±0.60
	150.00	6	1.50±0.76	36.45±2.21	25.51±24.89	0.67±0.21	32.00±0.71	5.85±0.90	2.17±0.65	16.40±5.86
IR 80156 B	0.00	6	1.00±0.00	39.00±1.00	10.25±0.25	0.00±0.00			0.67±0.33	10.80±0.62
	150.00	6	0.60±0.24	32.67±1.20	13.23±2.66	1.80±0.20	33.20±2.49	5.08±0.43	2.40±0.40	17.12±3.91
IR 82396 H	0.00	6	1.00±0.00	37.17±1.96	9.55±1.28	1.00±0.41	38.33±3.48	5.42±0.49	2.00±0.41	14.58±3.20
	150.00	6	1.50±0.62	33.75±1.30	11.74±1.39	2.67±0.67	35.84±2.27	5.06±0.75	4.17±0.48	33.02±5.74
IR46 R	0.00	6	0.50±0.29	40.50±6.50	11.60±4.30	0.75±0.25	35.00±3.46	3.90±0.35	1.25±0.25	8.73±2.81
	150.00	6	1.60±0.40	35.00±1.59	11.63±0.99	0.80±0.37	33.67±1.76	4.08±0.82	2.40±0.51	26.04±6.71
F-Accession (A)[2]				0.278	0.421		1.567	1.290	2.859	1.218
F-Nitrogen (F)[2]				3.626	0.495		0.036	0.029	9.009**>	5.62**>
F-A×F[2]				0.897	0.452		0.071	0.265	1.066	0.816
DF Error				16	16		18	18	26	26
IR 79156 A	0.00	6	0.67±0.33	34.50±0.50a	7.75±1.95	1.00±0.58	31.50±2.50	5.20±0.20	1.67±0.67	10.30±4.70
	150.00	3	0.67±0.33	33.50±3.50	8.40±1.10	2.00±0.58	33.33±2.40	2.64±0.42	2.67±0.67	11.03±3.92
IR 79156 B	0.00	6	1.00	36.00±ab	4.60±		±	±	0.25±0.25	6.40±0.60
	150.00	6	0.80±0.58	35.17±0.83	9.05±1.45	1.20±0.37	35.13±2.33	2.79±0.73	2.00±0.32	13.78±4.76
IR 82391 H	0.00	6	1.33±0.42	35.40±0.60ab	9.17±1.08	2.00±0.68	34.98±2.02	4.12±0.30	3.33±0.80	20.57±5.02
	150.00	6	0.50±0.22	35.33±0.88	10.83±0.78	2.33±0.76	34.18±1.39	6.00±0.35	2.83±0.70	17.93±3.42
IR60819-34-2 R	0.00	6	0.67±0.21	41.75±1.60b	10.25±2.25	0.83±0.48	39.67±5.78	3.91±0.49	1.50±0.34	10.13±1.70
	150.00	6	1.67±0.33	36.50±2.02	11.50±0.62	1.33±0.33	36.50±3.04	3.87±0.52	3.00±0.58	25.07±5.85
F-Accession (A)[2]				3.935*	1.930		1.068	5.537**	1.275	2.048
F-Nitrogen (F)[2]				2.124	2.426		0.032	0.301	2.997>	2.808>
F-A×F[2]				1.165	0.300		0.361	9.907***	1.222	1.022
DF Error				14	13		19	19	25	25
IR 80559 A	0.00	6	0.40±0.24	36.50±2.50	6.25±3.65	1.20±0.37	35.13±2.44	4.74±0.52	1.60±0.24	7.90±2.28ab
	150.00	4	2.50±0.65	35.98±2.07	13.44±0.68	2.00±0.41	33.25±1.27	5.35±0.44	4.50±0.50	43.65±7.59
IR 80559 B	0.00	6	0.40±0.24	33.00±3.00	10.65±1.75	1.00±0.32	37.50±2.33	3.35±0.50	1.40±0.24	7.74±2.08a
	150.00	6	0.33±0.33	39.00±	9.80±	3.00±1.00	35.25±2.32	5.34±1.12	3.33±0.20	17.13±5.11
IR 84714 H	0.00	6	1.40±0.51	37.79±3.33	8.18±1.14	1.40±0.51	38.54±1.51	4.60±0.76	2.80±0.58	17.24±4.14b
	150.00	6	1.00±0.45	34.17±1.71	11.02±0.86	2.00±0.68	38.81±0.61	4.81±0.27	3.00±0.77	20.33±4.58
IR60819-34-2 R	0.00	6	0.67±0.21	41.75±1.60	10.25±2.25	0.83±0.48	39.67±5.78	3.91±0.49	1.50±0.34	14.02±1.82a

	150.00	6	1.67±0.33	36.50±2.02	11.50±0.62	1.33±0.33	36.50±3.04	3.87±0.52	3.00±0.58	4.22±0.14
F-Accession (A)[2]				0.783	0.275		1.493	1.385	0.869	5.059**
F-Nitrogen (F)[2]				0.112	4.176		0.830	2.832	14.132***	8.149*
F-A×F[2]				1.157	1.840		0.149	1.121	2.223	11.548***
DF Error				16	16		21	21	29	29
IR 80564 A	0.00	3	0.33±0.33	41.00±	13.10±b	1.00±0.58	32.00±0.00	4.18±0.63	1.33±0.33	8.33±2.47ab
	150.00	5	0.40±0.24	38.00±1.00	13.60±2.60ab	2.00±0.55	34.00±1.68	4.09±0.65	2.40±0.51	16.50±1.86
IR 80564 B	0.00	6	0.50±0.34	37.25±1.25	9.33±0.53	2.00±0.52	35.60±1.77	4.57±0.47	2.50±0.72	12.83±4.02a
	150.00	6	0.20±0.20	32.00±	5.60±0.00	1.00±0.32	33.25±1.49	4.75±0.61	1.20±0.20	5.54±0.27
IR 85471 H	0.00	6	1.25±0.25	40.38±3.51	7.90±0.89ab	0.75±0.25	35.33±1.67	4.43±0.70	2.00±0.41	13.75±4.20b
	150.00	6	0.83±0.31	38.00±1.00	12.60±1.68	1.33±0.21	32.42±0.95	5.09±0.25	2.17±0.31	19.42±2.27
IR60819-34-2 R	0.00	6	0.75±0.48	41.25±6.75	8.73±0.33a	1.75±0.48	35.83±1.59	5.07±0.32	2.50±0.50	14.03±2.88a
	150.00	3	0.33±0.33	40.00±	4.30±	0.67±0.33	32.50±2.50	4.55±0.35	1.00±0.00	4.47±0.22
F-Accession (A)[2]				0.753	4.070*		0.220	0.491	0.393	4.784**
F-Nitrogen (F)[2]				0.980	0.288		1.693	0.012	1.167	0.519
F-A×F[2]				0.130	3.259		0.805	0.359	2.948*	6.488***
DF Error				17	17		23	23	28	28
IR 70369 A	0.00	6	0.25±0.25	41.00±	20.10±	1.75±0.25	37.63±4.38	4.78±0.37	2.00±0.41ab	13.55±6.17
	150.00	6	1.00±0.00	37.25±0.75	12.65±2.99	0.75±0.48	38.25±2.25	6.53±1.88	1.75±0.48	17.08±5.43
IR 70369 B	0.00	6	0.50±0.50	37.00±	8.80±	0.50±0.50	31.00±	3.60±	1.00±0.00a	6.20±2.60
	150.00	4	0.50±0.29	35.50±4.50	17.20±5.60	0.75±0.25	35.33±2.85	4.13±0.55	1.25±0.25	11.70±4.80
IR 81954 H	0.00	6	1.00±0.45	40.50±2.02	8.65±1.16	1.40±0.68	37.11±1.06	4.23±0.11	2.40±0.40ab	14.16±1.52
	150.00	6	1.00±0.32	34.13±0.83	14.10±1.99	0.40±0.24	36.00±4.00	4.85±0.75	1.40±0.24	16.80±5.21
IR72889-46-3-2-1 R	0.00	4	1.00±0.00	36.50±0.65	8.18±1.71	0.75±0.25	34.67±5.17	4.60±1.01	1.75±0.25b	11.63±2.72
	150.00	5	1.00±0.45	31.67±1.01	8.05±1.64	2.20±0.37	33.43±1.24	4.21±0.12	3.20±0.20	18.78±2.62
F-Accession (A)[2]				3.371*	2.899		0.735	2.205	4.275*	1.435
F-Nitrogen (F)[2]				10.730**	0.509		0.126	1.541	0.081	1.905
F-A×F[2]				0.546	2.128		0.126	0.987	4.232*	0.287
DF Error				14	14		15	15	25	25
IR 73328 A	0.00	6	0.67±0.33	38.50±4.50	9.50±1.70	1.00±0.00	37.00±0.58	2.67±0.37	1.67±0.33	12.27±0.71
	150.00	6	1.00±0.55	36.11±1.98	10.04±2.29	1.40±0.68	33.94±1.02	4.79±0.14	2.40±0.51	16.76±4.48
IR 73328 B	0.00	6	0.20±0.20	42.00±	8.20±	2.00±0.45	34.23±1.63	4.66±0.58	2.20±0.37	10.36±1.83
	150.00	6	1.00±0.32	34.88±2.38	12.25±1.53	1.00±0.32	31.75±0.85	4.81±0.63	2.00±0.55	17.42±5.61
IR 80637 H	0.00	6	1.00±0.32	37.63±2.97	10.25±1.60	0.80±0.49	36.50±0.50	3.78±1.13	1.80±0.58	13.08±4.15
	150.00	6	0.67±0.21	36.50±0.96	14.15±1.59	0.83±0.31	32.50±1.26	4.40±0.38	1.50±0.34	13.27±3.23

IR73013-95-1-3-2 R	0.00	2	0.67±0.33	39.00±6.00	9.20±4.40	0.67±0.33	33.00±0.00	3.80±1.00	1.33±0.33	8.67±2.60
	150.00	6	1.33±0.21	37.92±1.07	10.68±1.55	1.17±0.48	38.29±2.06	25.47±25.05	2.50±0.50	19.35±3.74
F-Accession (A)[2]				0.140	0.660		1.651	0.588	0.512	0.131
F-Nitrogen (F)[2]				1.951	2.319		1.200	0.691	0.777	2.309
F-A×F[2]				0.470	0.301		3.420	0.583	0.982	0.875
DF Error				18	18		20	20	30	30
IR 79125 A	0.00	5	0.80±0.37	34.00±2.00	9.27±0.79	0.60±0.40	29.75±1.25	3.33±0.98	1.40±0.24	9.52±3.15
	150.00	6	0.33±0.21	35.50±0.50	9.00±0.30	1.50±0.72	32.32±1.65	5.15±0.40	1.83±0.65	10.42±3.19
IR 79125 B	0.00	2	0.67±0.33	43.50±6.50	8.40±3.00	0.33±0.33	39.00±	3.30±	1.00±0.00	6.70±2.43
	150.00	4	1.25±0.63	39.33±5.36	8.00±1.32	1.25±0.48	33.33±1.33	3.70±0.21	2.50±0.96	16.00±8.27
IR 82385 H	0.00	6	0.20±0.20	40.00±	7.80±0.00	1.80±0.58	36.30±2.06	3.34±0.56	2.00±0.63	9.75±2.97
	150.00	6	1.00±0.26	35.90±1.57	10.31±1.74	1.67±0.21	36.17±1.05	3.94±0.49	2.67±0.33	16.88±2.91
IR73717-46-1-3-3 R	0.00	6	0.60±0.24	37.67±2.73	9.47±3.11	0.80±0.20	41.25±1.70	3.23±0.71	1.40±0.24	9.83±2.54
	150.00	6	0.80±0.37	36.33±1.33	11.42±1.39	1.00±0.55	34.67±2.67	6.10±0.92	1.80±0.37	14.08±2.62
F-Accession (A)[2]				1.236	0.403		4.509*	1.255	1.224	0.315
F-Nitrogen (F)[2]				0.679	0.357		2.206	7.846*	4.354*	4.094*
F-A×F[2]				0.353	0.217		2.234	1.440	0.446	0.460
DF Error				14	14		20	20	31	31
IR 68897 A	0.00	6	0.33±0.21	32.50±1.50	7.25±3.25	1.50±0.34	35.60±0.78	3.94±0.65	1.83±0.31ab	7.93±0.71a
	150.00	6	1.25±0.48	34.33±1.45	12.52±1.66	1.75±0.25	34.50±1.44	4.43±0.52	2.40±0.68	22.70±3.71
IR 68897 B	0.00	3	0.00±0.00	±	±	1.00±0.00	31.67±1.20	4.30±0.55	1.00±0.00ab	4.30±0.55a
	150.00	6	1.17±0.48	33.88±1.53	9.42±1.22	1.83±0.48	35.83±1.92	3.98±0.30	3.00±0.37	18.90±5.30
IR 82363 H	0.00	6	0.83±0.31	39.38±1.14	10.43±1.74	1.17±0.48	34.92±2.02	3.49±0.49	2.00±0.52a	12.65±3.74a
	150.00	6	1.40±0.40	38.25±1.31	10.31±1.19	0.60±0.40	34.50±2.50	4.40±0.60	2.00±0.32	17.50±3.85
SRT 3 R	0.00	6	1.40±0.51	37.17±1.42	11.39±2.02	1.00±0.32	33.50±1.85	4.23±0.49	2.40±0.24b	20.60±5.05b
	150.00	6	2.20±0.49	37.53±3.63	11.64±0.73	1.80±0.58	35.58±1.93	5.03±0.35	4.00±0.55	35.20±6.79
F-Accession (A)[2]				2.246	0.913		0.246	0.554	4.256*	5.858***
F-Nitrogen (F)[2]				0.017	1.887		0.970	1.544	18.845***	19.794***
F-A×F[2]				0.202	1.429		0.953	0.516	2.309	1.234
DF Error				19	19		23	23	32	32

1: Numbers are means ± standard errors (N ≤ 6, see column 3); lowercase letters indicate homogenous groups based on Tukey tests (P > 0.05); no analyses were conducted on the numbers of females and males

2: Degrees of freedom: accession = 7, nitrogen = 1, interaction = 7, error DF are indicated in the table; numbers are F-values, * = P ≤ 0.05, ** = P ≤ 0.01, *** = P ≤ 0.005

Table S14. Plant biomass and estimated weight losses for eight hybrid lines and their associated parental lines after infestation with YSB (see Table 9)

Accession	Added Nitrogen (Kg ha-1)[1]	Number of Replicated Blocks	Plant Condition (Proportion Alive)[1,2]	Total Number of Tillers at Harvest	Grain Biomass (Dry g)[1]	Shoot Biomass (Dry g)[1]	Root Biomass (Dry g)[1]	Plant Biomass Loss (Dry g)[1,3]	Plant Biomass Loss (Proportion)[1,3]	Plant Biomass Loss per mg of BPH (g mg-WBPH^{-1})[1,3]
IR 80156 A	0.00	2	1.00±0.00	3.00±0.00		2.42±0.39	0.50±0.06	6.46±1.27	0.69±0.01	0.48±0.11
	150.00	6	1.00±0.00	4.83±0.79		5.10±0.94	0.74±0.09	7.67±2.17	0.50±0.14	0.95±0.58
IR 80156 B	0.00	6	1.00±0.00	3.67±0.67	1.28±0.24	1.63±0.30	0.35±0.11	3.36±1.07	0.37±0.11	0.27±0.08
	150.00	6	1.00±0.00	5.40±0.60	1.67±0.57	3.87±1.08	0.47±0.12	4.88±2.42	0.30±0.12	0.64±0.51
IR 82396 H	0.00	6	1.00±0.00	5.00±0.91	1.07±0.14	2.66±0.25	0.47±0.06	2.93±1.06	0.31±0.08	0.24±0.09
	150.00	6	1.00±0.00	7.50±1.18	1.35±0.17	3.17±0.37	0.64±0.12	8.38±0.72	0.51±0.04	0.32±0.08
IR46 R	0.00	6	1.00±0.00	3.75±0.75	1.26±0.07	2.82±0.73	0.47±0.13	3.84±0.63	0.37±0.06	0.58±0.15
	150.00	6	1.00±0.00	4.20±0.58	1.72±0.18	3.17±0.31	0.43±0.04	8.16±1.20	0.47±0.03	1.05±0.82
F-Accession (A)[4]				2.603	0.516	0.387	1.086	0.841	1.058	0.516
F-Nitrogen (F)[4]				4.549*	0.498>	8.327**	1.580	17.552***>	14.965***>	0.984
F-A×F[4]				0.276	0.314	0.774	0.488	0.458	1.500	0.077
F-Covariate[4]								9.395***	38.515***	
DF Error				26	20	26	26	28	28	29
IR 79156 A	0.00	6	1.00±0.00	4.00±0.58		4.40±0.97b	0.55±0.14b	3.27±3.01	0.16±0.45	0.29±0.45
	150.00	3	1.00±0.00	4.33±0.33		4.68±0.28	0.56±0.10	4.27±2.27	0.38±0.15	0.87±0.70
IR 79156 B	0.00	6	1.00±0.00	1.50±0.29	1.26±0.23	1.50±0.19a	0.30±0.08a	5.53±0.18	0.55±0.04	0.89±0.28
	150.00	6	1.00±0.00	3.80±0.80	0.95±0.20	2.02±0.32	0.29±0.05	8.14±2.11	0.69±0.06	0.70±0.11
IR 82391 H	0.00	6	1.00±0.00	5.00±0.63	0.98±0.16	2.97±0.24b	0.54±0.09b	3.66±0.81	0.36±0.06	0.37±0.21
	150.00	6	1.00±0.00	4.83±0.83	0.88±0.29	4.87±1.34	0.60±0.12	6.64±1.83	0.50±0.11	0.37±0.10
IR60819-34-2 R	0.00	6	0.83±0.17	2.67±0.84	0.99±0.25	1.45±0.27a	0.23±0.04a	4.53±1.83	0.40±0.15	0.61±0.30
	150.00	6	1.00±0.00	6.00±1.15	1.64±0.44	2.39±0.28	0.36±0.04	5.54±3.12	0.44±0.25	0.18±0.11
F-Accession (A)[4]				1.324	1.071	8.396***	5.933***	0.891	1.632	0.829
F-Nitrogen (F)[4]				3.544	0.017	3.222	0.991	1.255>	0.941>	0.002
F-A×F[4]				1.829	1.408	0.305	0.214	0.366	0.443	0.927
F-Covariate[4]								11.414***	15.063***	
DF Error				25	21	25	25	24	24	25
IR 80559 A	0.00	6	1.00±0.00	3.20±0.73		2.94±0.17	0.44±0.02	2.73±0.84ab	0.40±0.09ab	0.40±0.15
	150.00	4	1.00±0.00	5.50±0.87		4.50±1.27	0.76±0.28	6.36±1.48	0.55±0.12	0.17±0.07
IR 80559 B	0.00	6	1.00±0.00	3.00±0.45	0.00±0.00	4.03±0.68	0.40±0.06	1.41±1.10a	0.15±0.24a	0.44±0.37
	150.00	6	1.00±0.00	5.33±0.33	0.00±0.00	5.56±0.70	0.72±0.10	4.32±1.92	0.36±0.11	0.43±0.30
IR 84714 H	0.00	6	0.80±0.20	4.40±1.12	0.87±0.23	3.25±0.75	0.43±0.13	3.58±1.43b	0.31±0.09b	0.25±0.12

	150.00	6	1.00±0.00	5.67±0.61	1.72±0.55	6.13±1.01	0.65±0.14	7.42±2.44	0.39±0.12	0.45±0.22
IR60819-34-2 R	0.00	6	0.83±0.17	2.67±0.84	0.61±0.08	1.47±0.19	0.33±0.07	8.54±0.98b	0.72±0.03b	0.79±0.29
	150.00	6	1.00±0.00	6.00±1.15	3.64±0.59	4.30±0.58	0.70±0.25	2.39±3.31	0.10±0.20	0.59±0.78
F-Accession (A)[4]				0.481	26.780***	2.256	0.118	6.022***	3.077*	1.679
F-Nitrogen (F)[4]				14.114***	17.338***	13.385***	7.898**	14.163***	5.231*	1.325
F-A×F[4]				0.555	7.357***	0.932	0.118	0.725	0.441	0.542
F-Covariate[4]								20.026***	16.608***	4.299*
DF Error				29	22	29	29	27	27	27
IR 80564 A	0.00	3	1.00±0.00	4.00±0.00		2.66±0.89b	0.28±0.11b	4.03±1.38a	0.57±0.17ab	0.63±0.26a
	150.00	5	1.00±0.00	4.20±0.97		5.05±1.19	0.68±0.23	6.20±1.03	0.53±0.10	0.40±0.07
IR 80564 B	0.00	6	0.83±0.17	1.83±0.48	0.86±0.22	1.02±0.24a	0.19±0.02a	3.76±0.52ab	0.43±0.05a	0.44±0.13ab
	150.00	6	0.80±0.20	2.60±0.98	1.55±0.48	1.49±0.36	0.21±0.04	7.78±1.51	0.54±0.07	1.39±0.22
IR 85471 H	0.00	6	0.75±0.25	2.75±0.95	0.21±0.08	2.54±0.83b	0.49±0.06b	7.13±1.89ab	0.58±0.09ab	0.70±0.34a
	150.00	6	1.00±0.00	5.00±1.13	1.61±0.24	4.02±0.44	0.44±0.05	8.41±1.36	0.50±0.07	0.50±0.12
IR60819-34-2 R	0.00	6	1.00±0.00	3.50±0.29	0.69±0.07	1.60±0.24ab	0.35±0.11ab	9.91±1.63b	0.70±0.03b	1.01±0.50b
	150.00	3	1.00±0.00	4.33±0.67	1.92±0.58	2.90±0.22	0.27±0.01	9.15±1.32	0.55±0.07	2.03±0.19
F-Accession (A)[4]				2.543	0.976	6.076***	4.347*	9.117***	6.958***	6.221***
F-Nitrogen (F)[4]				1.524	16.345***	8.104**	0.830	16.572***	18.524***	4.669*
F-A×F[4]				0.440	1.491	0.281	1.963	0.656	0.567	3.814*
F-Covariate[4]								15.591***	77.406***	
DF Error				28	28	28	28	23	23	24
IR 70369 A	0.00	6	1.00±0.00	3.50±0.50		3.55±0.72b	0.55±0.20	5.63±0.65	0.56±0.03	0.64±0.22
	150.00	6	1.00±0.00	3.75±0.85		4.32±0.83	0.51±0.12	8.60±1.80	0.58±0.12	0.72±0.34
IR 70369 B	0.00	6	1.00±0.00	3.50±0.50	1.23±0.67	2.05±0.40ab	0.52±0.06	5.49±1.79	0.49±0.13	0.93±0.10
	150.00	4	1.00±0.00	3.75±0.25	1.78±0.31	3.31±0.32	0.45±0.07	4.91±5.24	0.00±0.37	0.69±0.82
IR 81954 H	0.00	6	1.00±0.00	4.40±0.24	1.00±0.06	1.67±0.17a	0.26±0.04	4.92±1.40	0.48±0.08	0.39±0.12
	150.00	6	1.00±0.00	5.00±0.71	3.09±0.42	3.95±0.44	0.59±0.07	3.99±2.78	0.16±0.17	0.49±0.24
IR72889-46-3-2-1 R	0.00	4	1.00±0.00	3.25±0.63	1.09±0.21	1.70±0.35a	0.26±0.07	2.84±0.79	0.34±0.04	0.20±0.05
	150.00	5	1.00±0.00	4.80±1.16	1.77±0.28	3.24±0.25	0.49±0.07	10.17±2.88	0.44±0.14	0.59±0.18
F-Accession (A)[4]				1.156	1.793	3.280*	1.004	0.404	0.525	0.518
F-Nitrogen (F)[4]				1.029	18.493***	20.734***	3.265	5.147*	0.919	0.091
F-A×F[4]				0.292	2.735	1.350	2.159	0.778	1.151	0.202
F-Covariate[4]								4.094	6.739*	
DF Error				25	19	25	25	23	23	24
IR 73328 A	0.00	6	0.67±0.33	3.00±1.73		2.80±1.43	0.40±0.17ab	5.51±1.61	0.53±0.02	0.44±0.09

	150.00	6	1.00±0.00	5.40±0.40		4.90±0.55	0.53±0.06	6.43±1.35	0.51±0.07	0.70±0.38
IR 73328 B	0.00	6	1.00±0.00	3.60±0.60	0.94±0.18	1.72±0.32	0.34±0.09a	5.69±0.75	0.62±0.07	0.65±0.15
	150.00	6	1.00±0.00	3.40±0.75	1.41±0.50	2.50±0.83	0.24±0.06	5.60±0.47	0.54±0.07	0.59±0.25
IR 80637 H	0.00	6	0.80±0.20	3.40±1.03	0.68±0.26	1.73±0.61	0.32±0.08ab	4.48±0.47	0.51±0.07	0.40±0.19
	150.00	6	1.00±0.00	6.00±0.68	2.00±0.22	2.91±0.29	0.34±0.02	6.29±0.78	0.53±0.05	0.89±0.39
IR73013-95-1-3-2 R	0.00	2	1.00±0.00	4.67±1.67	1.95±0.09	2.84±0.34	0.40±0.00b	4.45±0.49	0.41±0.04	0.58±0.10
	150.00	6	1.00±0.00	4.67±0.56	1.52±0.22	3.82±0.27	0.66±0.13	6.83±0.73	0.52±0.04	0.40±0.07
F-Accession (A)[4]				0.847	2.013	2.508	3.154*	0.372	0.352	0.110
F-Nitrogen (F)[4]				4.159*	2.714	7.415*	1.185	5.499*	6.634*	0.368
F-A×F[4]				1.535	3.984	0.848	1.331	0.366	0.343	0.528
F-Covariate[4]								3.221	56.533***	
DF Error				30	24	30	30	25	25	26
IR 79125 A	0.00	5	1.00±0.00	2.20±0.49		3.99±0.78b	0.56±0.14b	6.51±0.67	0.60±0.06	0.88±0.17
	150.00	6	1.00±0.00	3.67±0.42		6.02±1.08	0.57±0.16	7.66±1.66	0.52±0.10	0.98±0.27
IR 79125 B	0.00	2	0.67±0.33	3.33±2.03	0.47±0.37	1.44±0.14a	0.25±0.06a	7.93±1.64	0.71±0.13	1.67±0.67
	150.00	4	0.75±0.25	2.50±1.04	0.80±0.31	1.78±0.66	0.30±0.08	9.69±12.03	-0.91±1.54	0.23±0.13
IR 82385 H	0.00	6	1.00±0.00	2.80±0.86	1.05±0.34	2.43±0.47ab	0.35±0.05ab	3.91±1.37	0.39±0.10	0.53±0.13
	150.00	6	1.00±0.00	5.33±0.92	1.11±0.20	3.64±0.61	0.67±0.10	9.94±3.42	0.36±0.08	0.87±0.48
IR73717-46-1-3-3 R	0.00	6	1.00±0.00	3.60±0.40	1.38±0.25	2.68±0.34ab	0.63±0.08b	2.89±0.83	0.27±0.08	0.54±0.34
	150.00	6	0.80±0.20	3.80±1.11	1.43±0.44	3.05±0.78	0.60±0.04	5.04±3.65	0.25±0.19	0.34±0.36
F-Accession (A)[4]				0.789	2.222	5.920***	3.674*	0.648	1.270	0.131
F-Nitrogen (F)[4]				2.329	0.313	1.528	1.244	10.022***	0.001<	1.033
F-A×F[4]				1.454	0.234	0.456	1.295	1.110	2.080	1.468
F-Covariate[4]								11.530***	5.424*	8.396**
DF Error				31	22	31	31	29	29	29
IR 68897 A	0.00	6	0.67±0.21	2.33±0.76a		3.77±1.10b	0.38±0.11b	2.93±2.07	0.22±0.30	0.39±0.25ab
	150.00	6	1.00±0.00	5.20±0.37		6.54±0.87	0.76±0.16	6.42±2.14	0.42±0.10	0.35±0.14
IR 68897 B	0.00	3	1.00±0.00	2.33±0.33ab	0.76±0.31	1.12±0.33a	0.25±0.10a	5.98±0.59	0.67±0.10	1.41±0.11b
	150.00	6	1.00±0.00	5.00±0.45	0.85±0.11	1.83±0.10	0.21±0.03	9.61±1.89	0.67±0.05	0.65±0.15
IR 82363 H	0.00	6	0.83±0.17	3.00±0.82a	0.80±0.27	1.57±0.31ab	0.28±0.06ab	3.91±0.78	0.45±0.07	0.33±0.10ab
	150.00	6	1.00±0.00	3.60±0.40	1.48±0.35	4.41±0.87	0.75±0.20	6.22±1.95	0.36±0.09	0.71±0.42
SRT 3 R	0.00	6	1.00±0.00	4.20±0.86b	1.09±0.16	1.62±0.16a	0.39±0.09ab	6.57±0.69	0.58±0.04	0.39±0.09a
	150.00	6	1.00±0.00	7.00±0.71	1.81±0.33	3.51±0.39	0.53±0.14	5.86±2.04	0.37±0.11	0.15±0.04
F-Accession (A)[4]				3.385*	2.409	7.579***	3.404*	0.573	1.532	4.722**
F-Nitrogen (F)[4]				19.459***	4.463*>	22.028***	9.665**	10.052***	8.572**	1.211

F-A×F[4]	0.973	0.476	0.594	2.033	0.445	1.136	2.416
F-Covariate[4]					6.950*	21.393***	
DF Error	32	24	32	32	30	30	31

1: Numbers are means ± standard errors (N ≤ 6, see column 3); lowercase letters indicate homogenous groups based on Tukey tests (P > 0.05)
2: Condition was not assessed using SES due to poor resolution of the system for YSB
3: Biomass loss was estimated for whole plants using grain to shoot conversion calculated as Biomass of A-line – Biomass of B-line/Biomass of B-line grain (see text for further details)
4: Degrees of freedom: accession = 7, nitrogen = 1, interaction = 7, covariate (plant biomass equivalent) = 1, error DF are indicated in the table; numbers are F-values, * = P ≤ 0.05, ** = P ≤ 0.01, *** = P ≤ 0.005

Table S15. Summary of results for YSB fitness on hybrid lines (see Table S9 for full details)

Accession	Added Nitrogen (Kg ha-1)[1]	Number of Female Moths Emerged[1]	Time to Adult Female Emergence (Days)[1]	Adult Female Biomass (Dry mg)[1]	Number of Male Moths Emerged[1]	Time to Adult Male Emergence (Days)[1]	Adult Male Biomass (Dry mg)[1]	Total Number of Emerged Adults[1]	Biomass of Emerged Adults[1] (Dry mg)
IR 82396 H	0.00	1.00±0.00	37.17±1.96	9.55±1.28	1.00±0.41	38.33±3.48	5.42±0.49	2.00±0.41	14.58±3.20
	150.00	1.50±0.62	33.75±1.30	11.74±1.39	2.67±0.67	35.84±2.27	5.06±0.75	4.17±0.48	33.02±5.74
IR 82391 H	0.00	1.33±0.42	35.40±0.60ab	9.17±1.08	2.00±0.68	34.98±2.02	4.12±0.30	3.33±0.80	20.57±5.02
	150.00	0.50±0.22	35.33±0.88	10.83±0.78	2.33±0.76	34.18±1.39	6.00±0.35	2.83±0.70	17.93±3.42
IR 84714 H	0.00	1.40±0.51	37.79±3.33	8.18±1.14	1.40±0.51	38.54±1.51	4.60±0.76	2.80±0.58	17.24±4.14b [Hb B; Ht A]†
	150.00	1.00±0.45	34.17±1.71	11.02±0.86	2.00±0.68	38.81±0.61	4.81±0.27	3.00±0.77	20.33±4.58
IR 85471 H	0.00	1.25±0.25	40.38±3.51	7.90±0.89	0.75±0.25	35.33±1.67	4.43±0.70	2.00±0.41	13.75±4.20b [Hb B; Ht A]†
	150.00	0.83±0.31	38.00±1.00	12.60±1.68	1.33±0.21	32.42±0.95	5.09±0.25	2.17±0.31	19.42±2.27
IR 81954 H	0.00	1.00±0.45	40.50±2.02	8.65±1.16	1.40±0.68	37.11±1.06	4.23±0.11	2.40±0.40ab	14.16±1.52
	150.00	1.00±0.32	34.13±0.83	14.10±1.99	0.40±0.24	36.00±4.00	4.85±0.75	1.40±0.24	16.80±5.21
IR 80637 H	0.00	1.00±0.32	37.63±2.97	10.25±1.60	0.80±0.49	36.50±0.50	3.78±1.13	1.80±0.58	13.08±4.15
	150.00	0.67±0.21	36.50±0.96	14.15±1.59	0.83±0.31	32.50±1.26	4.40±0.38	1.50±0.34	13.27±3.23
IR 82385 H	0.00	0.20±0.20	40.00±	7.80±0.00	1.80±0.58	36.30±2.06	3.34±0.56	2.00±0.63	9.75±2.97
	150.00	1.00±0.26	35.90±1.57	10.31±1.74	1.67±0.21	36.17±1.05	3.94±0.49	2.67±0.33	16.88±2.91
IR 82363 H	0.00	0.83±0.31	39.38±1.14	10.43±1.74	1.17±0.48	34.92±2.02	3.49±0.49	2.00±0.52a [A/B]	12.65±3.74a [A/B]
	150.00	1.40±0.40	38.25±1.31	10.31±1.19	0.60±0.40	34.50±2.50	4.40±0.60	2.00±0.32	17.50±3.85
F-Accession (A)[2]			1.151	0.776		1.456	2.507	1.979	1.275
F-Nitrogen (F)[2]			6.730*	13.343**		2.123	6.666*	0.346	5.411*
F-A×F[2]			0.538	0.716		0.331	0.876	1.380	0.758
DF Error			44	43		49	49	70	70

1: Numbers are means + standard errors (N = 6); lowercase letters indicate homogenous hybrid line groups based on Tukey tests (P > 0.05); blue cells indicate heterosis (Ht) based on significantly lower value of parameter compared to parental lines based on A or B-lines as indicated in square brackets; red cells indicate heterobeltiosis for susceptibility based on significantly higher parameter values compared to the parental lines based on the B-line (Ht A indicates heterosis with the A-line); †indicates resistance is associated with the restorer line; for statistical results see Table S9

2: Degrees of freedom: accession = 7, nitrogen = 1, interaction = 7, error DF are indicated in the table; numbers are F-values, * = P ≤ 0.05, ** = P ≤ 0.01

Table S16. Summary of results for YSB effects on hybrid lines (see Table S10 for full details)

Accession	Added Nitrogen (Kg ha-1)[1]	**Plant Condition (Proportion Alive)[1,2]**	**Total Number of Tillers at Harvest**	**Grain Biomass (Dry g)[1]**	**Shoot Biomass (Dry g)[1]**	**Root Biomass (Dry g)[1]**	**Plant Biomass Loss (Dry g)[1,3]**	**Plant Biomass Loss (Proportion)[1,3]**	Plant Biomass Loss per mg of YSB (g mg-YSB^{-1})[1,3]
IR 82396 H	0.00	1.00±0.00	5.00±0.91	1.07±0.14ab	2.66±0.25	0.47±0.06	2.93±1.06ab	0.31±0.08	0.24±0.09
	150.00	1.00±0.00	7.50±1.18	1.35±0.17	3.17±0.37	0.64±0.12	8.38±0.72	0.51±0.04	0.32±0.08
IR 82391 H	0.00	1.00±0.00	5.00±0.63	0.98±0.16a	2.97±0.24b	0.54±0.09b	3.66±0.81ab	0.36±0.06	0.37±0.21
	150.00	1.00±0.00	4.83±0.83	0.88±0.29	4.87±1.34	0.60±0.12	6.64±1.83	0.50±0.11	0.37±0.10
IR 84714 H	0.00	0.80±0.20	4.40±1.12	0.87±0.23ab	3.25±0.75	0.43±0.13	3.58±1.43ab [B][5]	0.31±0.09 [B][5]	0.25±0.12
	150.00	1.00±0.00	5.67±0.61	1.72±0.55	6.13±1.01	0.65±0.14	7.42±2.44	0.39±0.12	0.45±0.22
IR 85471 H	0.00	0.75±0.25	2.75±0.95	0.21±0.08a	2.54±0.83b	0.49±0.06b	7.13±1.89b	0.58±0.09	0.70±0.34
	150.00	1.00±0.00	5.00±1.13	1.61±0.24	4.02±0.44	0.44±0.05	8.41±1.36	0.50±0.07	0.50±0.12
IR 81954 H	0.00	1.00±0.00	4.40±0.24	1.00±0.06b	1.67±0.17a	0.26±0.04	4.92±1.40a	0.48±0.08	0.39±0.12
	150.00	1.00±0.00	5.00±0.71	3.09±0.42	3.95±0.44	0.59±0.07	3.99±2.78	0.16±0.17	0.49±0.24
IR 80637 H	0.00	0.80±0.20	3.40±1.03	0.68±0.26ab	1.73±0.61	0.32±0.08ab	4.48±0.47ab	0.51±0.07	0.40±0.19
	150.00	1.00±0.00	6.00±0.68	2.00±0.22	2.91±0.29	0.34±0.02	6.29±0.78	0.53±0.05	0.89±0.39
IR 82385 H	0.00	1.00±0.00	2.80±0.86	1.05±0.34ab	2.43±0.47	0.35±0.05ab	3.91±1.37ab	0.39±0.10	0.53±0.13
	150.00	1.00±0.00	5.33±0.92	1.11±0.20	3.64±0.61	0.67±0.10	9.94±3.42	0.36±0.08	0.87±0.48
IR 82363 H	0.00	0.83±0.17	3.00±0.82a	0.80±0.27ab	1.57±0.31ab	0.28±0.06ab	3.91±0.78ab	0.45±0.07	0.33±0.10
	150.00	1.00±0.00	3.60±0.40	1.48±0.35	4.41±0.87	0.75±0.20	6.22±1.95	0.36±0.09	0.71±0.42
F-Accession (A)[4]			1.905	2.372*	2.097	1.365	2.477*	1.488	1.038
F-Nitrogen (F)[4]			11.438***	29.567***	32.396***	15.171***	38.792***	26.163***	6.309*
F-A×F[4]			0.841	2.888**	0.835	1.664	2.054	1.614	0.674
F-Covariate[4]							28.720***	64.451***	5.190*
DF Error			64	64	64	64	64	64	64

1: Numbers are means ± standard errors (N = 6); lowercase letters indicate homogenous groups based on Tukey tests (P > 0.05); blue cells indicated heterosis based on comparisons with B-lines
2: Condition was not assessed using SES due to poor resolution of the system for WBPH 3: Biomass loss was estimated for whole plants using grain to shoot conversion calculated as Biomass of A-line – Biomass of B-line/Biomass of B-line grain (see text for further details)
4: Degrees of freedom: accession = 7, nitrogen = 1, interaction = 7, covariate (plant biomass equivalent) = 1, error DF are indicated in the table; numbers are F-values, * = P ≤ 0.05, *** = P ≤ 0.005
5: Heterobeltiosis for susceptibility under high nitrogen.
